# Integrated Phenotypic and Genotypic Analysis Reveals Distinct Patterns of Biofilm Formation and Antimicrobial Resistance in Poultry-Derived *Campylobacter jejuni* and *Campylobacter coli*

**DOI:** 10.3390/foods15213815

**Published:** 2026-10-25

**Authors:** Dimitra Kostoglou, Eleni Mucka, Nikolaos D. Andritsos, Efstathios Giaouris

**Affiliations:** 1 Laboratory of Food Microbiology and Hygiene, Department of Food Science and Nutrition, School of the Environment, University of the Aegean, 81400 Myrina, Lemnos, Greece; 2 Laboratory of Food Safety, Quality and Applied Biotechnology, Department of Food Science and Technology, School of Agricultural Sciences, University of Patras, 30100 Agrinio, Aetolia-Acarnania, Greece

**Keywords:** food safety, *Campylobacter*, biofilm formation, antibiotic resistance, virulence factors, genetic diversity, association analysis, rep-PCR

## Abstract

*Campylobacter* (*C*.) *jejuni* and *C. coli* are major foodborne bacterial pathogens, and multiple mechanisms, including biofilm formation, antimicrobial resistance (AMR), and genetic variability, contribute to their persistence in poultry-associated environments. However, the relationships among these traits remain poorly understood. This study investigated 120 *Campylobacter* isolates (78 *C. jejuni* and 42 *C. coli*) from retail raw chicken meat by integrating assessments of biofilm formation under two different atmospheric conditions, antibiotic susceptibility profiles, AMR and virulence-associated determinants, and repetitive sequence-based polymerase chain reaction (rep-PCR)-based genetic diversity. Biofilm formation was highly heterogeneous, with atmospheric composition being associated with biofilm biomass accumulation rather than the overall occurrence of biofilm-forming isolates. Although comparable proportions of biofilm formers were detected under microaerophilic (47.5%) and aerobic carbon dioxide (CO_2_)-enriched (45.8%) conditions, increased oxygen was associated with isolate-specific shifts toward increased biofilm biomass, with no significant overall difference. High resistance frequencies were observed for tetracycline (TE) and ciprofloxacin (CIP), with TE + CIP representing the predominant combined resistance phenotype; however, biofilm formers did not show broader AMR profiles and were less often resistant to multiple antimicrobial classes. Genotype–phenotype analyses showed strong associations between *gyrA* mutation and CIP resistance and between *tet(O)* detection and TE resistance, whereas the multidrug efflux-associated gene *cmeB* showed only a weak, species-dependent association with antimicrobial response complexity and not with a single resistance phenotype. Overall, biofilm formation was mainly associated with species, with a weaker association with rep-PCR cluster, and was not associated positively with AMR among this collection of poultry-associated *Campylobacter* isolates. These findings support assessing biofilm-forming capacity and AMR separately in these bacteria, rather than relying on one trait to infer the other. They also suggest that *Campylobacter*, inherently susceptible to antimicrobials, may favor biofilm formation as an alternative survival strategy.

## 1. Introduction

Species in the genus *Campylobacter* are among the most important global foodborne bacterial pathogens [1,2,3]. Among them, *C. jejuni* and *C. coli* cause the vast majority of human campylobacteriosis cases and remain a persistent challenge for food safety systems worldwide [4]. Indeed, according to the most recent European Union (EU) One Health Zoonoses Report, campylobacteriosis remained the most frequently reported zoonotic disease in humans in 2024, surpassing salmonellosis and all other monitored bacterial zoonoses, while reported cases have increased significantly over the last five years [5]. Although infections are generally self-limiting, a recent global meta-analysis estimated that 13.8% (95% CI: 9.6–18.6) of reported cases require hospitalization, underscoring the substantial public health burden associated with this pathogen [6]. Consequently, *Campylobacter* spp. remain a major focus of epidemiological surveillance, risk assessment, and food safety interventions [5].

The ecological success of thermotolerant *Campylobacter* species is closely linked to poultry production systems, which serve as their primary reservoir and are major vehicles for transmission to humans [7,8]. In particular, broiler chickens are efficiently colonized without developing clinical disease, facilitating bacterial dissemination throughout the production continuum and increasing opportunities for human exposure through contaminated poultry products [9,10,11]. Environmental reservoirs within poultry production systems, including drinking water, litter, insects, rodents, equipment, and farm personnel, substantially contribute to *Campylobacter* introduction and spread within broiler flocks [8,12]. Consequently, environmental persistence is increasingly recognized as a critical factor in *Campylobacter* epidemiology and transmission dynamics. Nevertheless, *Campylobacter*’s widespread occurrence throughout the food production continuum presents an apparent ecological paradox [13]. This is because, despite its demanding growth requirements and limited tolerance to several environmental stresses under laboratory conditions [14,15], the organism is repeatedly recovered from diverse food-associated environments and remains detectable at multiple stages of the food chain [1,16]. This contradiction has spurred growing interest in biofilm formation as a possible key adaptive mechanism that supports environmental survival and persistence [13,17,18,19].

Biofilms are well-recognized microbial communities embedded in a self-produced extracellular matrix and typically attached to biotic or abiotic surfaces [20]. The transition from planktonic to sessile growth enhances bacterial tolerance to adverse environmental conditions and is associated with increased survival during challenges such as oxygen exposure, nutrient limitation, desiccation, cleaning procedures, and disinfectant treatment encountered in food-processing environments [9,11,21]. Consequently, biofilm-associated populations may serve as persistent reservoirs of contamination within food-processing facilities [9,21]. Nevertheless, *Campylobacter*’s biofilm-forming phenotype remains remarkably heterogeneous [13]. Species identity, strain background, growth conditions, atmospheric composition, interspecies interactions, and methodological differences have all been proposed as factors contributing to the highly variable biofilm phenotypes reported in the literature [18,22,23,24,25]. However, the relative contributions of these factors to biofilm development and persistence remain incompletely understood.

Beyond biofilm entrapment, AMR has also emerged as a major factor in *Campylobacter* ecology and public health significance [26]. Resistance to fluoroquinolones and tetracyclines has increased globally, while resistance to macrolides, although generally less prevalent, remains a particular concern because these compounds are among the preferred therapeutic options for severe human infections [27,28]. Recent EU-wide surveillance data showed high to extremely high levels of CIP resistance in both *C. jejuni* and *C. coli* from human and animal sources, leading the European Food Safety Authority (EFSA) and the European Centre for Disease Prevention and Control (ECDC) to conclude that fluoroquinolones can no longer be recommended for treating *Campylobacter* infections in humans [27]. The genetic architecture underlying AMR in *Campylobacter* encompasses multiple mechanisms, including target-site alterations, acquisition of resistance determinants, and multidrug efflux systems [29]. Among the best-characterized determinants are mutations in the *gyrA* gene, which are strongly associated with fluoroquinolone resistance; the tetracycline resistance gene *tet(O)*; *β*-lactamase genes such as *bla_OXA-61_*; the aminoglycoside-modifying enzyme gene *aphA-3-1*; and the *cmeABC* multidrug efflux operon, which collectively promote resistance and persistence under antimicrobial selection pressures [28,29].

However, *Campylobacter* complexity extends beyond AMR, encompassing a diverse repertoire of virulence determinants involved in adhesion, invasion, toxin production, environmental persistence, and host colonization [30], including the cytolethal distending toxin operon (*cdtABC*). This genus’s remarkable genetic diversity further contributes to substantial phenotypic heterogeneity among isolates [29,30]. Consistent with this diversity, molecular typing approaches have shown that *C. jejuni* and *C. coli* populations comprise distinct genetic lineages that differ in ecological fitness, host adaptation, and epidemiological behavior [31,32]. In this context, genomic investigations indicate that variation in AMR and virulence-associated gene repertoires may also influence isolate-specific biofilm phenotypes and environmental persistence [24,33,34,35]. Collectively, these observations indicate that these phenotypic and genotypic characteristics are embedded within the broader genetic background of *Campylobacter* spp., highlighting the need to investigate them as interconnected rather than as independent biological features [36,37].

Despite the extensive body of literature on biofilm formation, AMR, virulence determinants, and molecular epidemiology, relatively few studies have examined these characteristics within an integrated analytical framework [29,38], leaving the relationships among phenotypic traits, resistance determinants, and population structure incompletely understood [31,36]. Moreover, published findings on the association between biofilm formation and AMR remain inconsistent [13,19,33,38], whereas the contributions of specific resistance determinants and genetic backgrounds to environmental persistence and the co-occurrence of these phenotypic characteristics remain to be fully elucidated [32,36]. Consequently, current evidence indicates that *Campylobacter* ecological behavior arises from interactions among multiple phenotypic and genotypic attributes rather than from individual traits considered in isolation [29,33,34,38].

Addressing these knowledge gaps requires integrated phenotypic and genotypic approaches that can simultaneously evaluate multiple factors relevant to the environmental persistence and population diversity of *Campylobacter* pathogens. Therefore, this study investigated 78 *C. jejuni* and 42 *C. coli* isolates recovered from retail raw chicken meat in Greece, through the combined assessment of biofilm-forming capacity under microaerophilic and aerobic CO_2_-enriched conditions, antibiotic susceptibility phenotypes (EUCAST-interpreted resistance for three antibiotics and complete non-inhibition for other seven, from a panel of ten antibiotics belonging to eight different classes), the presence of six selected AMR- and virulence-associated genes, and rep-PCR-based genetic diversity. Association analyses were then conducted to identify relationships among these phenotypic, genotypic, and ecological characteristics, aiming to provide a more comprehensive understanding of the factors and interactions associated with environmental persistence, AMR, and population heterogeneity in poultry-associated *Campylobacter* populations.

## 2. Materials and Methods

### 2.1. Campylobacter Isolates and Preparation of Their Working Inocula

A total of 120 *Campylobacter* spp. isolates were included in this study, all derived from 54 *Campylobacter*-positive samples among 60 retail raw chicken meat samples (whole carcasses, minced meat, and parts; 30% prepackaged) purchased from three butcher shops and three supermarkets in Myrina (Lemnos Island, Greece) between July and October 2021 [39]. Table S1 lists all isolates belonging to the two most relevant species, i.e., *C. jejuni* (n = 78) and *C. coli* (n = 42), along with their rep-PCR clustering, as previously determined [39]. rep-PCR clusters comprise isolates with fingerprint similarity > 90% and represent groups of similar fingerprints, not confirmed epidemiological lineages. Isolates originating from the same sample are identifiable by the POUL code in Table S1. All isolates, along with the *C. jejuni* ATCC 33291 strain (a human clinical isolate), were stored frozen at −80 °C in Mueller–Hinton (MH) broth (Oxoid Limited, Thermo Fisher Scientific Inc., Waltham, MA, USA) containing 20% *v/v* glycerol (Merck KGaA, Darmstadt, Germany) and 5% *v/v* laked horse blood (HB; Thermo Fisher Scientific Inc.). To recover them, the isolates were subcultured onto *Campylobacter* blood agar (CBA; Biolife Italiana, Milano, Italy) plates and incubated under microaerophilic conditions (6.2–13.2% O_2_, 2.5–9.5% CO_2_; Oxoid *Campy*Gen 2.5L Sachet; Thermo Fisher Scientific Inc.) at 42 °C for 24 ± 1 h (extended to 40–48 h when required to achieve sufficient growth). For each isolate, a loopful of bacterial growth was then inoculated into 2 mL of MH broth and incubated under the same conditions to obtain working cultures. Bacterial cells from the latter cultures were harvested by centrifugation at 5000× *g* for 10 min at 4 °C, using a Frontier 5000 Series Multi Pro centrifuge (FC5718R, OHAUS Europe GmbH, Nänikon, Switzerland), washed twice with sterile quarter-strength Ringer’s solution (Lab M, Heywood, Lancashire, UK), and resuspended to an optical density at 600 nm (OD_600_) of 0.1 (approximately 10^8^ CFU/mL). The inoculum was further diluted in quarter-strength Ringer’s solution to achieve a final concentration of 10^6^ CFU/mL.

### 2.2. Determination of Biofilm-Forming Capacity

Biofilm-forming capacity was assessed using the classical crystal violet (CV) staining assay, as previously described [40]. Τwo growth media were used to support biofilm formation: MH broth supplemented with 5% *v/v* sterile chicken juice (MH-CJ) and MH broth supplemented with 5% *v/v* HB (MH-HB). Chicken juice was prepared as described previously [23] and stored at −80 °C until use. MH-CJ was selected to simulate the poultry-associated environment, whereas MH-HB, commonly used for *Campylobacter* cultivation, was included for comparison. For biofilm formation, 180 μL of the respective growth medium and 20 μL of the respective saline bacterial suspension for each isolate were added to each well of a sterile 96-well polystyrene (PS) microtiter plate (transparent, flat-bottom, Cat. No. 30096, SPL Life Sciences Co., Ltd., Naechon-Myeon, Pocheon-si, Gyeonggi-do, Republic of Korea), yielding a final inoculum concentration of approximately 10^5^ CFU/mL. As a negative control (NC), 20 μL of sterile quarter-strength Ringer’s solution was used instead of the bacterial suspension. The surface of each plate was loosely wrapped in Parafilm, and the plates were incubated at 42 °C for 48 h under either microaerophilic (Oxoid *Campy*Gen™ 2.5L Sachet) or aerobic CO_2_-enriched (15% O_2_, 3.5–9.0% CO_2_; Oxoid CO_2_Gen™ 2.5L Sachet; Thermo Fisher Scientific Inc.) conditions. After incubation, the OD_600_ of the planktonic suspensions in each well was measured using the Tecan Spark^®^ multimode microplate reader (Tecan Group Ltd., Männedorf, Switzerland), and the suspensions were then discarded. Wells were then gently washed twice with sterile quarter-strength Ringer’s solution to remove non-adherent and loosely attached cells. Remaining biofilms in each well were stained with 0.1% (*w/v*) CV solution (1% *w/v*, aqueous solution; Sigma-Aldrich Chemie GmbH, Taufkirchen, Germany), and the bound dye was solubilized with an ethanol/acetone (80:20, *v/v*) mixture. The total biofilm biomass was measured at 590 nm (A_590_). Each isolate was evaluated in at least two independent biological experiments, each with two technical replicates, and the mean A_590_ value was used for subsequent analyses. Cut-off absorbance (Ac) was defined as three standard deviations above the mean A_590_ of the NC and was determined separately for each growth medium and atmospheric condition. All the isolates were finally classified as non-biofilm formers (A_590_ ≤ Ac), weak biofilm formers (Ac < A_590_ ≤ 2× Ac), moderate biofilm formers (2× Ac < A_590_ ≤ 4× Ac) and strong biofilm formers (4× Ac < A_590_), based on a previously described approach [41].

### 2.3. Determination of Antibiotic Susceptibility

In vitro antibiotic susceptibility was evaluated using the European Committee on Antimicrobial Susceptibility Testing (EUCAST)-standardized disk diffusion method for erythromycin (E), tetracycline (TE), and ciprofloxacin (CIP) [42]. Additional antibiotics without established EUCAST clinical breakpoints for *Campylobacter* spp., including gentamicin (GEN), trimethoprim-sulfamethoxazole (SXT), ampicillin (AMP), norfloxacin (NOR), meropenem (MEM), cefotaxime (CTX), and ceftazidime (CAZ), were included to evaluate inhibition profiles and patterns of antibacterial activity. For the disk diffusion method, MH agar (Oxoid Limited, Thermo Fisher Scientific Inc.) plates supplemented with 5% *v/v* defibrinated horse blood (Remel™, Thermo Fisher Scientific Inc.) and 20 mg/L *β*-nicotinamide adenine dinucleotide (NAD; F. Hoffmann-La Roche AG, Basel, Switzerland) were used. For each isolate, its bacterial suspension was adjusted to an OD_600_ of 0.1 (approximately equivalent to a 0.5 McFarland standard) and uniformly swabbed onto the agar surface. Plates were incubated under microaerophilic conditions (Oxoid *Campy*Gen™ 2.5L Sachet) at 42 °C for 24 ± 1 h. Isolates with insufficient growth after 24 h were re-incubated, and inhibition zones were read after a total incubation time of 40–48 h. Inhibition zone diameters (mm) were measured at the point of complete growth inhibition, viewed from the front of the plate under reflected light, according to EUCAST guidelines. *C. jejuni* ATCC 33291 was used as a reference strain, and each isolate was tested once, in accordance with the EUCAST disk diffusion protocol. For antibiotics without EUCAST breakpoints for *Campylobacter* spp. (EUCAST Clinical Breakpoint Tables v. 16.0, valid from 1 January 2026), inhibition zone diameters were recorded and analyzed without clinical categorization. Because no clinical breakpoints or epidemiological cut-off values are available for these antibiotics, isolates were not classified as susceptible or resistant. Instead, the absence of a visible inhibition zone around the disc (recorded as an inhibition zone diameter of 1 mm; disc diameter 6 mm) was used as an objective, reader-independent criterion, and such isolates were classified as “non-inhibition”. Isolates with any measurable zone were considered inhibited. This classification describes in vitro antibacterial activity only and does not represent clinical resistance. Table 1 presents the antibiotics tested, their classes, disc content, and interpretive criteria.

### 2.4. Detection of AMR and Virulence-Associated Genes

#### 2.4.1. DNA Extraction from *Campylobacter* Isolates

Genomic DNA (gDNA) from *Campylobacter* was isolated using a previously described protocol [39] with slight modifications. Briefly, each isolate was streaked onto an MH-HB agar plate and incubated under microaerophilic conditions at 42 °C for 24 h (preculture stage). A loopful of each preculture was transferred to 2 mL of fresh MH broth in an Eppendorf^®^ tube and incubated under the same conditions at 42 °C for 24 h (working cultures; final concentration of approximately 10^8^ CFU/mL). This procedure was repeated twice for each isolate. After incubation, the two working cultures of each isolate were centrifuged at 5000× *g* for 10 min at 4 °C using the Frontier 5000 Series Multi Pro centrifuge. The pellets were combined and washed with sterile ultrapure (Type I) water (Direct-Pure Water System, UP 10 UV, Rephile Bioscience Inc., Shanghai, China) by centrifugation. The gDNA from each washed bacterial pellet was then isolated using the PureLink™ Genomic DNA Mini Kit (Thermo Fisher Scientific Inc.). The concentration of each extracted gDNA sample was determined using the Qubit™ 4 Fluorometer (Thermo Fisher Scientific Inc.). A 5 μL aliquot was also analyzed by agarose gel electrophoresis (1.5% *w/v* Tris-borate-EDTA (TBE) agarose gel stained with ethidium bromide (EtBr); 100 V for 30 min) to verify DNA integrity, using the Mupid-One electrophoresis system (NIPPON Genetics EUROPE GmbH, Dueren, Germany) and FastGene^®^ 100 bp DNA Ladder (NIPPON Genetics EUROPE GmbH) as the molecular weight marker. After electrophoresis, stained gels were visualized under ultraviolet (UV) trans-illumination using the Quantum ST4 gel documentation imaging system (Vilber Lourmat, Marne-la-Vallée, France). The remaining volume of each gDNA sample was stored at −20 °C until use in subsequent PCR reactions. Two isolates (POUL046/CAMP093 and POUL047/CAMP095) were excluded from gene detection and subsequent genotype-based association analyses because the original DNA extracts had been exhausted and the corresponding bacterial stocks, which had been stored at −80 °C for an extended period, failed to recover upon subculture, preventing the extraction of new gDNA. Consequently, PCR amplification to detect AMR-associated and virulence genes was performed on the remaining 118 isolates.

#### 2.4.2. PCR Amplification

The AMR-associated genes *bla_OXA-61_* (confering resistance to beta-lactams, cephalosporins including), *tet(O)* (confering resistance to tetracyclines), *cmeB* (*Campylobacter* multidrug efflux pump gene), *aphA-3-1* (aminoglycoside-modifying enzyme), and the Thr-86-to-Ile point mutation in the *gyrA* gene (conferring resistance to fluoroquinolones), as well as the virulence-associated gene *cdtB* (part of the cytolethal distending toxin gene cluster *cdtABC*) were detected in *Campylobacter* isolates using single and multiplex PCR assays. These targets were selected as well-characterized determinants corresponding to the antimicrobials tested (CIP, TE, AMP and GEN), a marker of the CmeABC multidrug efflux system, and a virulence marker (*cdtB*), for which established PCR assays for *C. jejuni* and *C. coli* were available. The panel was designed to examine selected genotype–phenotype relationships and not to characterize the full resistome. The specific primers and expected amplicon sizes for each target gene are listed in Table S2 [43,44,45,46,47,48]. The Kapa Taq PCR Kit with dNTPs (F. Hoffmann-La Roche AG) was used for the PCR assays. The reaction mixture contained 2.5 μL of 10× Kapa Taq Buffer A (corresponding to a final MgCl_2_ concentration of 1.5 mM); 0.5 μL of 10 mM dNTP mix (final concentration of 0.2 mM); 1 μL of 10 μM forward primer (final concentration of 0.4 μM); 1 μL of 10 μM reverse primer (final concentration of 0.4 μM); 4 μL of DNA template (100 ng total DNA); 0.2 μL (1 U) of Kapa Taq DNA polymerase (5 U/μL); and 15.8 μL of PCR-grade water, resulting in a final reaction volume of 25 μL. To detect *cdtB* in *C. jejuni* and *C. coli*, primer concentrations were adjusted to final concentrations of 0.125 μM and 0.15 μM, respectively. Following optimization of single PCR conditions, an additional multiplex PCR (mPCR) approach was evaluated for the simultaneous detection of the AMR-associated genes *bla_OXA-61_*, *tet(O)*, *cmeB*, and *aphA-3-1*, based on similar primer annealing temperatures. Although simultaneous amplification of multiple targets was achieved, amplification consistency varied among targets, particularly for *aphA-3-1*. Therefore, isolates yielding weak, incomplete, or ambiguous multiplex PCR profiles were subsequently re-evaluated using single PCR assays to confirm gene detection. PCR amplification conditions consisted of an initial denaturation at 94 °C for 5 min, followed by 30 cycles of denaturation at 94 °C for 30 s, annealing at 50 °C for 30 s for *gyrA*-control, *gyrA*-mutation, *bla_OXA-61_*, *tet(O)*, and *cmeB*, targets, as well as for mPCR assays, at 51 °C for 30 s for *cdtB*, and at 54 °C for 30 s for *aphA-3-1*. Extension was performed at 72 °C for 20 s for *gyrA*-control and *gyrA*-mutation targets, at 72 °C for 30 s for *tet(O)*, *cmeB*, *aphA-3-1*, and *cdtB*, and at 72 °C for 60 s for *bla_OXA-61_* and mPCR assays, followed by a final extension step at 72 °C for 7 min. PCR products were visualized by electrophoresis on 2.0% (*w/v*) agarose gels prepared in TBE buffer and stained with EtBr. Electrophoresis was performed at 100 V for 30 and 50 min for single PCR and mPCR products, respectively, and amplified DNA fragments were subsequently visualized under UV transillumination.

### 2.5. Statistical Analyses

Statistical analyses were performed using *jamovi* (Version 2.6) [49], which is built on the R statistical environment (Version 4.4; R Project for Statistical Computing) [50]. Absorbance values (A_590_) under the two atmospheres were compared using the paired Wilcoxon signed-rank test. Associations between categorical variables were evaluated using contingency table analysis. Pearson’s chi-square (χ^2^) test was applied when assumptions were met, whereas Fisher’s exact test was used when expected cell counts did not meet χ^2^ assumptions or when exact testing was more appropriate for binary comparisons. Effect sizes for categorical associations were estimated using Cramér’ s V. Odds ratios (OR) were calculated from binary (2 × 2) contingency tables, and 95% confidence intervals (CI) were obtained by the Wald (logit) method to quantify the strength and direction of associations. For genotype-based analyses, only isolates for which both phenotypic and molecular data were available were included. The number of affected antimicrobial classes was calculated by counting resistance (E, TE, CIP) and complete non-inhibition (GEN, SXT, AMP, NOR, MEM, CTX, CAZ) phenotypes, with the two fluoroquinolones (CIP, NOR) and the two cephalosporins (CTX, CAZ) each counted as one class. Differences in the number of affected antimicrobial classes between groups were assessed using the Mann–Whitney U test. All statistical tests were two-sided, and statistical significance was defined as *p* < 0.05. Genotype-phenotype agreement was additionally expressed as overall concordance, sensitivity, specificity, and positive and negative predictive values (PPV, NPV), taking the phenotype as the reference. For CIP, isolates categorized as I were grouped with susceptible isolates in these analyses. To account for confounding by species, the association between rep-PCR cluster and biofilm formation (yes/no) was additionally evaluated, separately for each atmospheric condition, by Firth penalized logistic regression including species and rep-PCR cluster (F, H, J, and minor profiles; reference: F) as predictors, and likelihood ratio tests were used to assess the contribution of cluster after adjustment for species. Species-adjusted comparisons of the F cluster with the remaining clusters were made using Mantel–Haenszel odds ratios stratified by species. Isolates with unassigned cluster (ND) were excluded from these analyses. The association between biofilm formation (yes/no, per atmosphere) and antimicrobial response (≥2 EUCAST-resistant classes, susceptibility to E, TE and CIP, and high complexity) was evaluated using Fisher’s exact test, species-stratified Mantel–Haenszel odds ratios, and Firth logistic regression including species. All association analyses were exploratory, were not adjusted for multiple comparisons, and describe associations, not causal effects. Because the tests were exploratory and correlated, *p*-values were interpreted alongside effect sizes and confidence intervals; a sensitivity analysis applying Bonferroni, Holm and Benjamini–Hochberg corrections did not change the main conclusions.

## 3. Results

### 3.1. Assessment of the Biofilm-Forming Capacity

**Figure 1:**
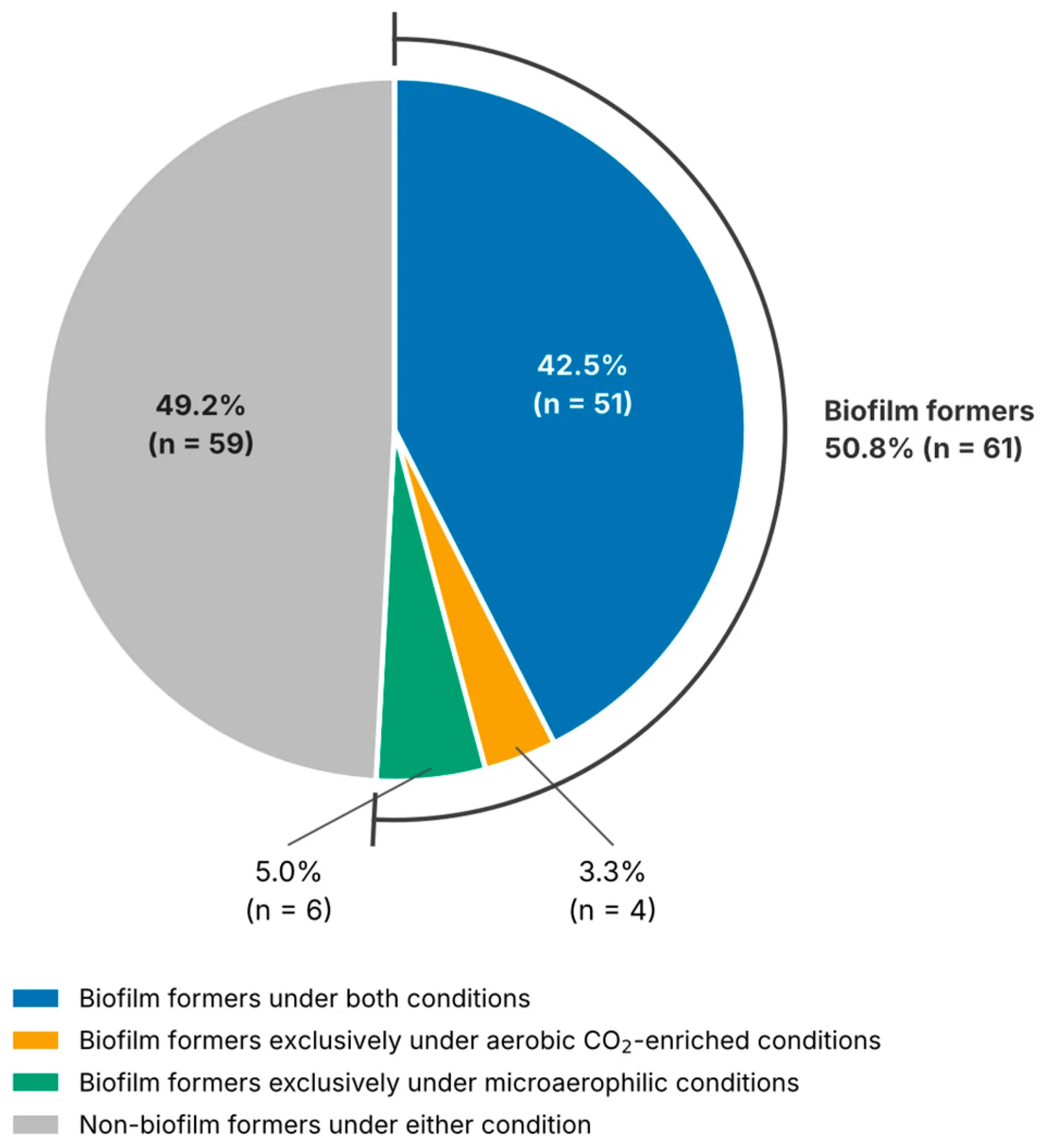
Distribution of *Campylobacter* spp. isolates (n = 120) by biofilm-forming phenotype under either microaerophilic (6.2–13.2% O_2_, 2.5–9.5% CO_2_) or aerobic CO_2_-enriched (15% O_2_, 3.5–9.0% CO_2_) conditions in MH–CJ medium (Mueller–Hinton broth + 5% chicken juice) after 48 h of incubation at 42 °C. Isolates were classified as biofilm formers (weak, moderate, or strong) when A_590_ exceeded the condition-specific cut-off (Ac = 0.78 under microaerophilic and 0.69 under aerobic CO_2_-enriched conditions; Table S3).

Table S3 presents the biofilm biomasses (A_590_) of the 120 *Campylobacter* isolates across the four tested conditions (incubation in MH-CJ or MH-HB broth at 42 °C for 48 h under microaerophilic or aerobic CO_2_-enriched conditions) and classifies each isolate as a non-biofilm former, weak, moderate, or strong biofilm former. No detectable biofilm formation was observed in MH–HB medium under either microaerophilic or aerobic CO_2_-enriched conditions (all A_590_ values were ≤Ac, i.e., ≤0.83 and ≤0.99, respectively). As all isolates remained below the cut-off in MH-HB, these data showed no phenotypic variability and could not support comparative or association analyses. They were therefore excluded from further analysis, and all subsequent biofilm analyses were based on MH-CJ. Biofilm formation was observed in that latter medium after 48 h of incubation under both microaerophilic and aerobic CO_2_-enriched conditions. At first, replicate variability was low to moderate (median coefficient of variation 14.9% and 15.0% in MH-CJ under microaerophilic and aerobic CO_2_-enriched conditions, respectively; Table S3). Atmospheric conditions had only a limited effect on the overall proportion of biofilm-forming isolates, with comparable percentages of biofilm/non-biofilm formers under microaerophilic (47.5%/52.5%) and aerobic CO_2_-enriched conditions (45.8%/54.2%). In other words, most biofilm-positive isolates (51/61; 83.6%) retained their ability to form biofilms under both atmospheric conditions. However, biofilm formation under both conditions did not necessarily correspond to identical biomass accumulation patterns. Figure 1 presents the distribution of aerobiosis-condition-dependent and condition-independent biofilm-forming phenotypes.

**Figure 2:**
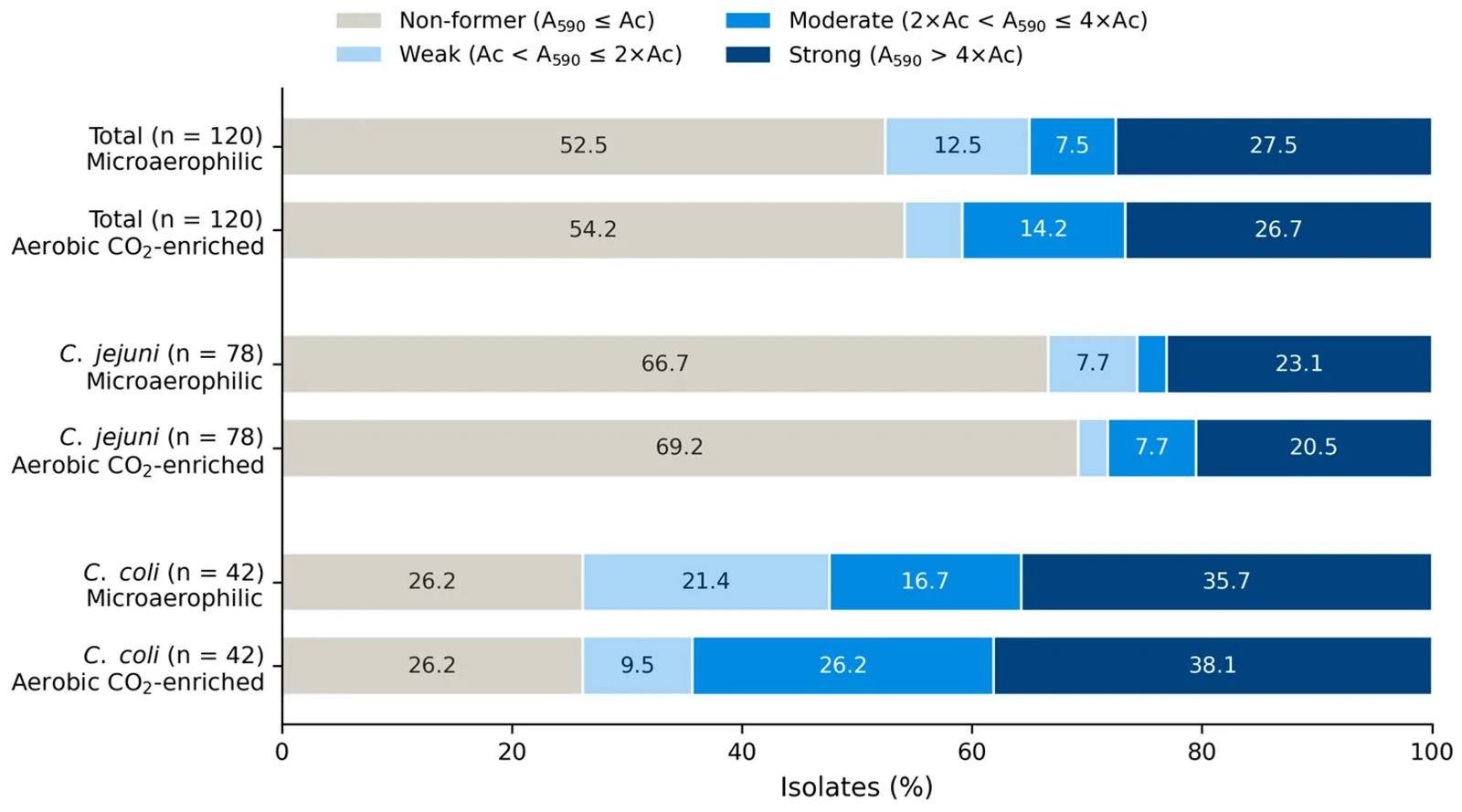
Distribution (%) of biofilm-forming phenotypes among total *Campylobacter* spp., *C. jejuni*, and *C. coli* isolates following incubation in MH-CJ medium (Mueller–Hinton broth + 5% chicken juice) under either microaerophilic (6.2–13.2% O_2_, 2.5–9.5% CO_2_) or aerobic CO_2_-enriched (15% O_2_, 3.5–9.0% CO_2_) conditions for 48 h at 42 °C. Isolates were classified as biofilm formers (weak, moderate, or strong) when A_590_ exceeded the condition-specific cut-off (Ac = 0.78 under microaerophilic and 0.69 under aerobic CO_2_-enriched conditions; Table S3).

Although the overall prevalence of biofilm-forming isolates remained largely unchanged, oxygen concentration appeared to influence the distribution of biofilm biomass accumulation patterns. Specifically, under aerobic CO_2_-enriched conditions, a shift from weak to moderate biofilm-forming capacity was observed (Figure 2), suggesting that increased oxygen is associated with greater biomass accumulation, although overall A_590_ values did not differ significantly (see below).

Figure 2 also showed species-specific differences. Under microaerophilic conditions, biofilm formation was observed in 33.3% of *C. jejuni* isolates and 73.8% of *C. coli* isolates (26/78 and 31/42, respectively). This difference was statistically significant (Fisher’s exact test, *p* < 0.001), with *C. coli* isolates exhibiting 5.64-fold higher odds of biofilm formation than *C. jejuni* isolates (OR = 5.64, 95% CI: 2.45–13.0). Similar proportions were recorded under aerobic CO_2_-enriched conditions, where 30.8% of *C. jejuni* and 73.8% of *C. coli* isolates were classified as biofilm formers (24/78 and 31/42, respectively). Likewise, the difference between species remained statistically significant under aerobic CO_2_-enriched conditions (Fisher’s exact test, *p* < 0.001), with *C. coli* isolates exhibiting 6.34-fold higher odds of biofilm formation than *C. jejuni* isolates (OR = 6.34, 95% CI: 2.74–14.7). In addition, *C. coli* isolates showed a more robust biofilm phenotype under aerobic CO_2_-enriched conditions, with a marked reduction in weak biofilm formers and increased proportions of moderate and strong biofilm formers. In contrast, *C. jejuni* isolates showed a slight reduction in the total number of biofilm-positive isolates under aerobic conditions; however, as with *C. coli*, weak biofilm formers decreased while moderate formers increased. Collectively, atmosphere was associated with isolate-specific shifts between biofilm categories, but A_590_ values did not differ significantly between atmospheres (overall median paired difference = 0.04, 95% CI: 0.00–0.06; *p* = 0.109; *C. jejuni p* = 0.175; *C. coli p* = 0.289; paired Wilcoxon test), and *C. coli* isolates showed higher biofilm formation under both conditions.

Additional differences in biofilm-forming phenotypes were observed among the major rep-PCR clusters under both atmospheric conditions (Table 2). Overall, the F rep-PCR cluster showed the strongest biofilm-associated phenotype, with the lowest proportion of non-biofilm formers (25.6% and 23.1% under microaerophilic and aerobic CO_2_-enriched conditions, respectively) and the highest combined proportion of weak, moderate, and strong biofilm-forming isolates (>74%) under both atmospheric conditions. More specifically, under aerobic CO_2_-enriched conditions, the proportion of strong biofilm formers within the F cluster increased from 33.3% to 41.0%, whereas weak biofilm formers decreased from 20.5% to 12.8%, reflecting a shift between categories. In contrast, the J rep-PCR cluster showed a comparatively weaker biofilm-associated phenotype, with most isolates classified as non-biofilm formers under both microaerophilic and aerobic CO_2_-enriched conditions (62.1% and 69.0%, respectively). Nevertheless, increased oxygen was associated with a redistribution of biofilm-forming phenotypes within this group: weak biofilm formers decreased from 20.7% to 3.4%, while moderate biofilm formers increased from 0.0% to 10.3%, and the overall prevalence of biofilm-positive isolates remained relatively unchanged. The H rep-PCR cluster demonstrated a comparatively stable phenotype distribution under both atmospheric conditions, with only minor shifts between moderate and strong biofilm-forming categories. In addition, isolates lacking rep-PCR classification (ND) exhibited predominantly non-biofilm-forming phenotypes irrespective of atmospheric conditions (with 84.6% of them classified as non-biofilm formers under either condition).

However, further analysis showed that species composition partially influenced the observed association between rep-PCR clustering and biofilm phenotype. In the F rep-PCR cluster, most weak, moderate, and strong biofilm-forming isolates corresponded to *C. coli* strains, representing 79.3% (23/29) and 76.7% (23/30) of all biofilm-positive isolates under microaerophilic and aerobic CO_2_-enriched conditions, respectively. Conversely, within the J rep-PCR cluster, 94% and 95% of non-biofilm-producing isolates under microaerophilic and aerobic CO_2_-enriched conditions, respectively, corresponded to *C. jejuni* strains. Species-specific exploratory analysis (Table S4) showed that isolates of the F rep-PCR cluster had numerically higher proportions of biofilm-positive phenotypes than the remaining clusters in both *C. jejuni* (54.5% and 63.6% vs. 33.9% and 28.8% under microaerophilic and aerobic CO_2_-enriched conditions, respectively) and *C. coli* (82.1% vs. 66.7% under both conditions), whereas the proportion of biofilm formers in the J cluster (37.0% and 29.6%) was similar to that of all 78 *C. jejuni* isolates (33.3% and 30.8%). Furthermore, atmosphere-dependent redistribution of biofilm phenotypes within individual rep-PCR clusters was also observed. For example, under aerobic CO_2_-enriched conditions, weak biofilm formers within the F cluster tended to decrease, whereas moderate and/or strong biofilm-producing phenotypes increased, reflecting a shift between categories. In contrast, the isolates in J cluster showed a shift from weak to moderate biofilm-forming phenotypes without a substantial increase in the overall proportion of biofilm-positive isolates. However, because species and rep-PCR cluster were strongly confounded (F: 28/39 *C. coli*; J: 27/29 *C. jejuni*), the association between cluster and biofilm formation was re-evaluated in species-adjusted analyses of the 107 isolates with an assigned cluster (Table S5). The association with cluster was significant unadjusted (likelihood ratio test, *p* < 0.001 under both atmospheres) but was no longer significant after adjustment for species under microaerophilic conditions (*p* = 0.11) and was weaker under aerobic CO_2_-enriched conditions (*p* = 0.032). Species remained associated with biofilm formation (adjusted OR = 3.70, 95% CI: 1.27–10.8, and 3.49, 95% CI: 1.20–10.2, respectively). Compared with the other clusters, the F cluster had higher species-adjusted odds of biofilm formation under aerobic CO_2_-enriched conditions (Mantel–Haenszel OR = 3.40, 95% CI: 1.20–9.66; *p* = 0.037) but not under microaerophilic conditions (OR = 2.33, 95% CI: 0.83–6.53; *p* = 0.18). The lower biofilm formation of the J cluster was not significant after adjustment (adjusted OR = 0.48, 95% CI: 0.14–1.61, and 0.30, 95% CI: 0.09–1.02, versus the F cluster).

Collectively, these findings indicate that species composition explained a substantial part of the association between rep-PCR cluster and biofilm-forming phenotype, whereas a residual, atmosphere-dependent association of the F cluster remained after adjustment. Given the small number of isolates per cluster, this should be interpreted cautiously.

### 3.2. Assessment of Antibiotic Susceptibility

#### 3.2.1. Impact of EUCAST Breakpoint Revisions on Antimicrobial Susceptibility Classification

Antimicrobial susceptibility classification of the 120 *Campylobacter* isolates was initially evaluated according to different EUCAST clinical breakpoint versions (2013, 2021, and 2026) that included relevant revisions of *C. jejuni* and *C. coli* susceptibility interpretation (EUCAST document archive; https://www.eucast.org/bacteria/document-archive/; accessed on 23 October 2026) in order to assess the impact of breakpoint revisions on susceptibility classification (Table 3). Overall, resistance to CIP remained consistently high (94/120; 78.3%) across all EUCAST versions. However, a substantial interpretative shift was observed following the 2021 EUCAST revision, where isolates previously classified as susceptible (S) were reclassified into the susceptible, increased exposure (I) category despite identical inhibition zone diameter measurements. More specifically, 26 isolates initially categorized as CIP-susceptible according to EUCAST v. 2013 were reclassified as susceptible, increased exposure according to EUCAST v. 2021 and 2026 criteria. Therefore, the observed differences reflected changes in clinical breakpoint interpretation rather than modifications in the experimentally obtained Antimicrobial Susceptibility Testing (AST) data. In contrast, E resistance remained low across all EUCAST versions, ranging between 2.5% and 3.3%, confirming the continued effectiveness of macrolides as first-line therapeutic agents against campylobacteriosis. TE resistance remained consistently high (69/120; 57.5%) irrespective of breakpoint version, indicating widespread resistance to this antimicrobial class among the examined isolates. Collectively, these findings demonstrate that revisions of EUCAST clinical breakpoints may substantially influence susceptibility categorization and epidemiological interpretation without necessarily reflecting actual changes in AMR phenotypes. Based on the most recent EUCAST recommendations, all subsequent antimicrobial susceptibility analyses presented in this study were performed according to EUCAST v. 2026 clinical breakpoints.

#### 3.2.2. EUCAST-Interpretable Resistance Patterns

**Figure 3:**
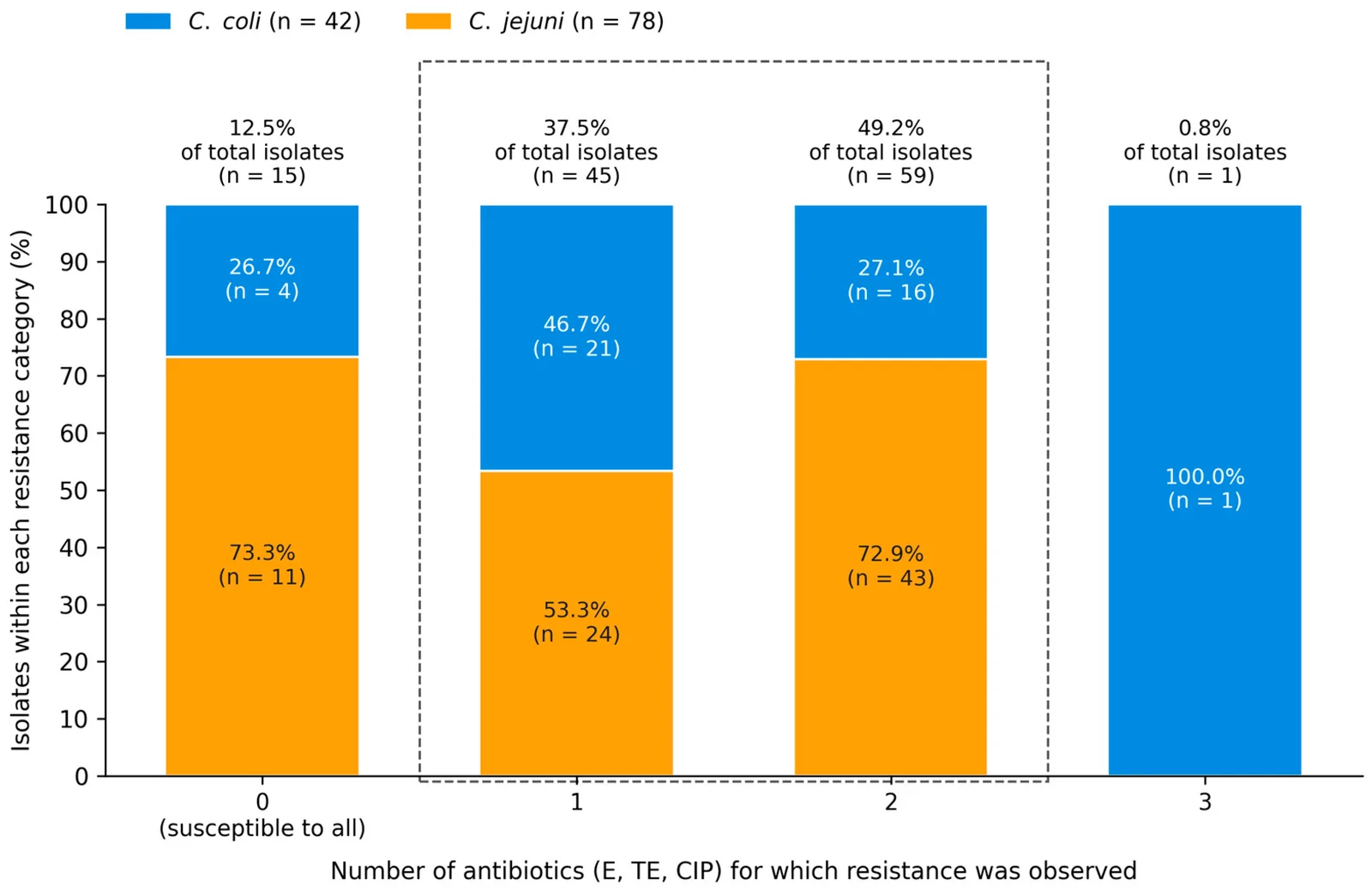
Distribution of *C. jejuni* and *C. coli* isolates by the number of antibiotics (0–3) for which resistance was observed, based on EUCAST v. 2026 clinical breakpoints for erythromycin (E), tetracycline (TE), and ciprofloxacin (CIP). Percentages above bars: share of the total collection (n = 120). Segments: relative contribution of each species within the category (n = isolate count). Dashed box: predominance of isolates resistant to one or two antibiotics.

The distribution of antibiotic response phenotypes among the examined *C. jejuni* and *C. coli* isolates was initially evaluated based on the three antibiotics for which EUCAST clinical breakpoints were available (i.e., E, TE, CIP). Overall, most isolates (104/120; 86.7%) were resistant to one or two antibiotics from different antibiotic classes, whereas only one *C. coli* isolate (POUL047/CAMP097; 0.8%) was resistant to all three antibiotics (Figure 3). In contrast, 15 isolates (12.5%) remained susceptible to all three EUCAST-interpretable antibiotics included in the analysis. Resistance to two antibiotics represented the most prevalent response category among the examined isolates (59/120; 49.2%), followed by resistance to one antibiotic (45/120; 37.5%). Among isolates resistant to two antibiotics, *C. jejuni* accounted for most cases (43/59; 72.9%), whereas *C. coli* represented a smaller proportion (16/59; 27.1%). Notably, resistance to two antibiotics was proportionally more prevalent among *C. jejuni* isolates (43/78; 55.1%) than among *C. coli* isolates (16/42; 38.1%), indicating a higher prevalence of combined resistance phenotypes among *C. jejuni* strains. Conversely, complete susceptibility to all three EUCAST-interpretable antibiotics was proportionally more frequent among *C. coli* isolates. The predominance of isolates resistant to two antibiotics was almost entirely attributable to the combined TE + CIP resistance phenotype, which accounted for 58 of 59 cases (98.3%). Therefore, further characterization of EUCAST-interpretable resistance profiles was performed to determine the specific antibiotic combinations contributing to the observed phenotypic distribution and their potential association with rep-PCR cluster distribution.

#### 3.2.3. Inhibition-Response Patterns Among Non-EUCAST Antibiotics

**Figure 4:**
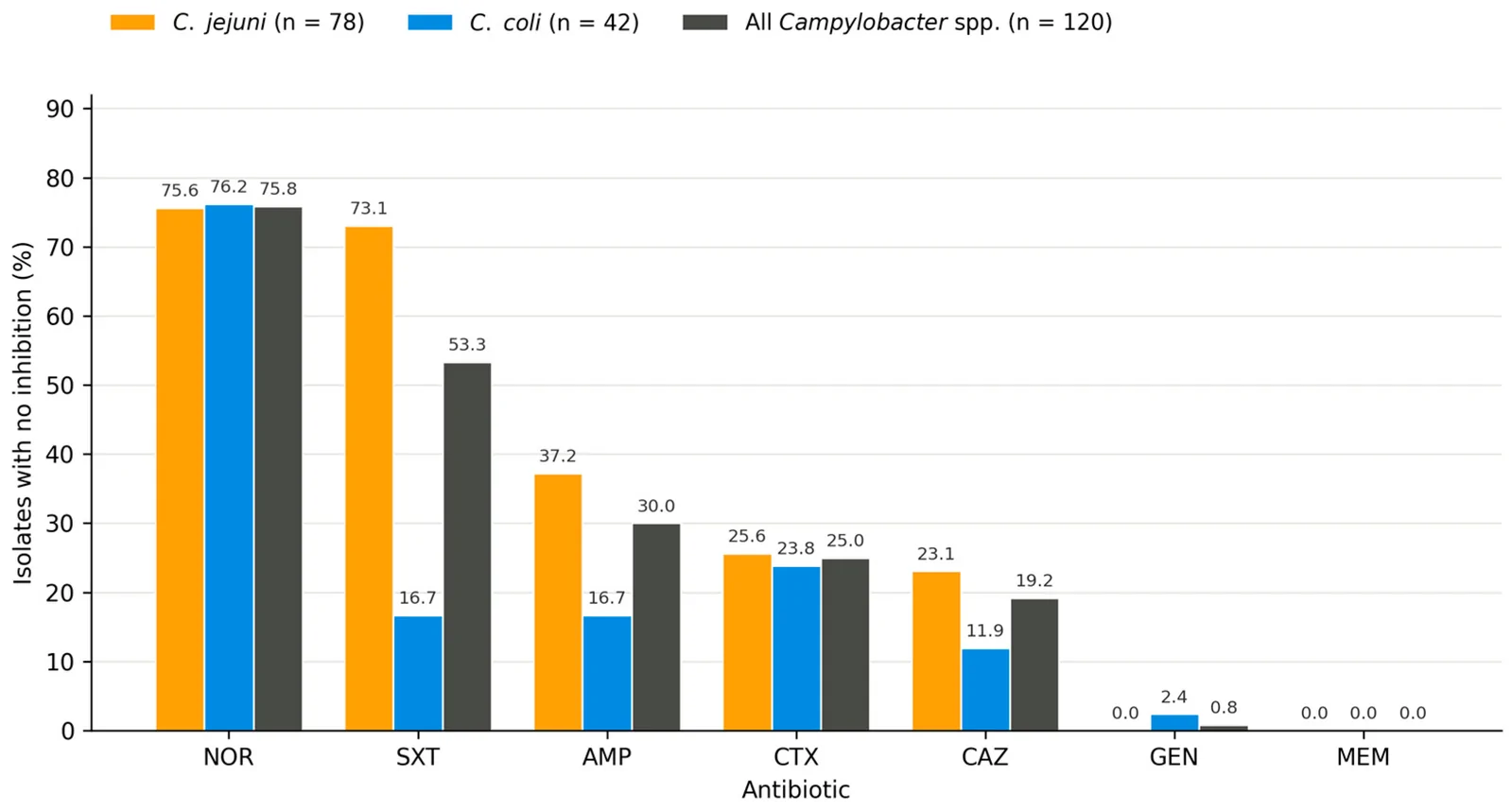
Frequency (%) of isolates exhibiting complete absence of visible inhibition by antibiotics lacking established EUCAST clinical breakpoints for *Campylobacter* spp. (NOR: norfloxacin; SXT: trimethoprim-sulfamethoxazole; AMP: ampicillin; CTX: cefotaxime; CAZ: ceftazidime; GEN: gentamicin; MEM: meropenem; disk contents in Table 1). No inhibition does not represent clinical resistance. Percentages are relative to the number of isolates for each species (n = 78 and n = 42 for *C. jejuni* and *C. coli*, respectively) or to the entire collection (n = 120). Absence of visible inhibition was recorded as an inhibition zone diameter of 1 mm.

In parallel, inhibition zone patterns were evaluated for antibiotics lacking established EUCAST clinical breakpoints for *Campylobacter* spp. (GEN, AMP, SXT, NOR, MEM, CAZ, and CTX) to further characterize additional inhibition–response phenotypes among the examined isolates. Because no breakpoints exist for these antibiotics, the data are not interpreted as resistance: “non-inhibition” denotes the complete absence of a visible inhibition zone, whereas “resistance” is used only for E, TE, and CIP interpreted by EUCAST v. 2026. Among the evaluated antibiotics, NOR showed the highest proportion of isolates with no visible inhibition, with comparable frequencies in both *C. jejuni* and *C. coli* populations (Figure 4). Elevated frequencies of absence of visible inhibition were also observed for SXT, whereas intermediate proportions were recorded for AMP, CTX, and CAZ. In contrast, nearly all isolates were visibly inhibited by GEN and MEM, for which absence of visible inhibition was rare (1/120) and not observed (0/120), respectively. Zone diameters of the inhibited isolates were continuously distributed and clearly separated from the non-inhibition entry, since the smallest measured zone for any antibiotic was 9 mm (Table S6). Overall, *C. jejuni* isolates showed higher frequencies of no inhibition by SXT, AMP, and CAZ than *C. coli* isolates, whereas NOR and CTX showed similar distributions between the two species. Notably, this species-related difference was particularly pronounced for SXT, with absence of visible inhibition in 73.1% (57/78) of *C. jejuni* isolates compared with only 16.7% (7/42) of *C. coli* isolates, suggesting reduced inhibitory effectiveness of SXT against *C. jejuni* isolates. This difference was statistically significant (Fisher’s exact test, *p* < 0.001; OR = 13.6, 95% CI: 5.23–35.2), indicating a strong association (Cramér’s V = 0.552).

**Figure 5:**
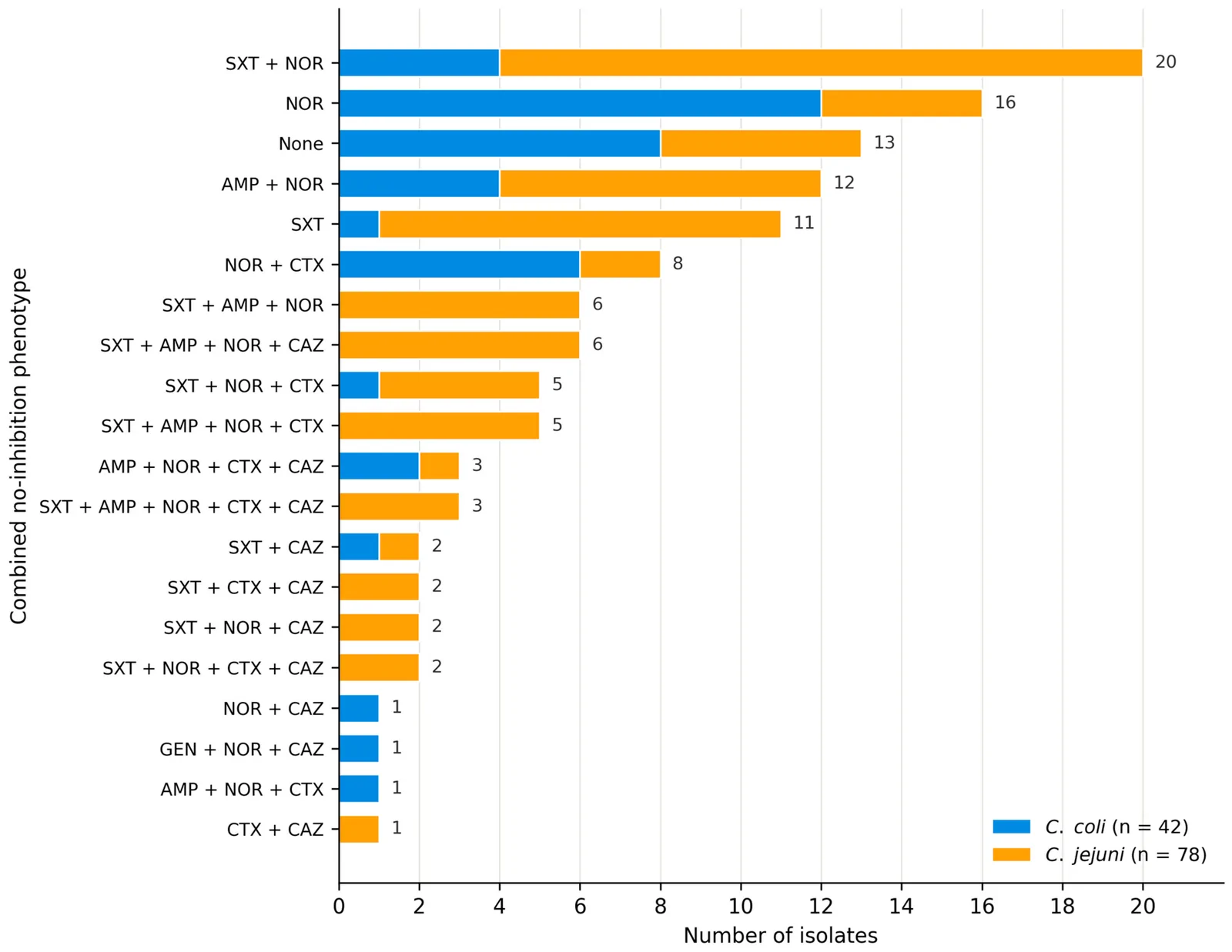
Distribution of combined no-inhibition phenotypes among *Campylobacter* isolates for antibiotics lacking established EUCAST clinical breakpoints (SXT: trimethoprim-sulfamethoxazole; NOR: norfloxacin; AMP: ampicillin; CTX: cefotaxime; CAZ: ceftazidime; GEN: gentamicin; disk contents in Table 1). “A + B” = no inhibition by both antibiotics simultaneously. No inhibition does not represent clinical resistance. Bars represent isolate counts for each phenotype, separated by *C. jejuni* and *C. coli* isolates (number at bar ends = total isolates per phenotype). “None” refers to isolates that did not exhibit a no-inhibition phenotype for any of the antibiotics included in this analysis. Absence of visible inhibition by a given antibiotic was recorded as an inhibition zone diameter of 1 mm.

To further investigate the distribution of additional inhibition-response phenotypes, combined no-inhibition patterns were analyzed across the examined isolates (Figure 5). The most prevalent phenotype was the combined SXT + NOR no-inhibition pattern, followed by the absence of visible inhibition by NOR alone and the AMP + NOR combination. Notably, NOR was included in most predominant combined phenotypes, indicating that lack of inhibition by NOR was a widespread characteristic among the examined isolates. Species-associated distribution trends were also observed among the combined no-inhibition phenotypes. In particular, *C. jejuni* isolates more frequently showed no visible inhibition by multiple antibiotics simultaneously, whereas *C. coli* isolates more commonly showed no visible inhibition by NOR alone. More specifically, 37.5% (12/32) of *C. coli* isolates showing absence of visible inhibition by NOR exhibited this phenotype exclusively for NOR, whereas the corresponding proportion among *C. jejuni* isolates was substantially lower (4/59; 6.8%). Conversely, combined no-inhibition phenotypes involving SXT together with NOR and/or additional antibiotics were predominantly associated with *C. jejuni* isolates, suggesting a tendency toward broader no-inhibition profiles within this species. Heatmap visualization of phenotype distribution patterns further highlighted this species-associated segregation, with SXT-containing multi-antibiotic no-inhibition phenotypes clustering predominantly among *C. jejuni* isolates, whereas simpler phenotypes involving NOR alone or NOR-associated combinations were more frequently associated with *C. coli* isolates (Table S7). More complex phenotypes involving three or more antibiotics were observed less frequently and were distributed across both *C. jejuni* and *C. coli* isolates. Although certain multi-antibiotic no-inhibition combinations, such as AMP + NOR + CTX + CAZ, showed a higher proportion of *C. coli* isolates (2/3; 66.7%), the overall frequency of this phenotype was low (n = 3), so it did not substantially alter the overall species-associated distribution trend. Collectively, these findings suggest that *C. jejuni* isolates tended to exhibit broader and more complex no-inhibition phenotypes involving multiple antibiotics, whereas *C. coli* isolates were more frequently associated with narrower phenotypes, particularly absence of visible inhibition by NOR alone.

#### 3.2.4. Association of Antibiotic Response Phenotypes with Rep-PCR Clusters

Analysis of EUCAST-interpretable resistance phenotypes according to rep-PCR clustering revealed distinct distribution trends (Table S8). At the total *Campylobacter* spp. level, the TE phenotype was mainly associated with the F cluster, which accounted for 77.8% of TE-resistant isolates. The TE + CIP phenotype, which represented the predominant combined resistance profile, was more broadly distributed among F, J, and minor rep-PCR profiles, while the J cluster accounted for the highest share of fully susceptible isolates (7/15; 46.7%). Species-specific analysis further showed that, among *C. jejuni*, the J cluster contributed the highest proportions of susceptible isolates, whereas the H cluster was mainly associated with TE-only resistance. In contrast, among *C. coli*, resistance phenotypes were predominantly concentrated in the F cluster, which included all TE-resistant isolates, most CIP-resistant isolates, and most TE + CIP-resistant isolates. These findings suggest that EUCAST-interpretable resistance phenotypes were not evenly distributed across rep-PCR clusters, with a more concentrated distribution among *C. coli* isolates and a broader distribution among *C. jejuni* isolates.

Extending the analysis to no-inhibition phenotypes for antibiotics lacking established EUCAST clinical breakpoints further revealed distinct rep-PCR-associated distribution trends (Table S9). At the total *Campylobacter* spp. level, the predominant SXT + NOR-associated phenotype was mainly linked to the J cluster, which accounted for 45.0% of isolates exhibiting this phenotype. Similarly, the SXT phenotype was strongly associated with the J cluster (63.6%). In contrast, the NOR and NOR + CTX phenotypes were predominantly associated with the F cluster, representing 50.0% and 75.0% of isolates within these phenotypes, respectively. The phenotype categorization presented in Table S7 indicated that SXT-associated phenotypes were predominantly linked to *C. jejuni* isolates, whereas NOR-associated phenotypes were more commonly associated with *C. coli* isolates. Consistent with these observations, Table S9 further demonstrated that SXT-associated phenotypes were mainly distributed within the J cluster, while NOR-associated phenotypes were more frequently associated with the F cluster. Species-specific analysis highlighted additional differences between *C. jejuni* and *C. coli*. Among *C. jejuni* isolates, the SXT + NOR phenotype, representing the dominant phenotype within the SXT + NOR-associated category, was primarily distributed within the J cluster (56.3%), followed by minor rep-PCR and ND profiles (31.3%). Similarly, the SXT phenotype was strongly associated with the J cluster (60.0%). In contrast, the NOR phenotype was mainly associated with the F cluster (50.0%), while the SXT + NOR + CTX phenotype was predominantly linked to the J cluster (75.0%). Expanded SXT + AMP + NOR-associated phenotypes showed broader genetic heterogeneity, with several phenotypes mainly distributed among minor rep-PCR and ND profiles, including SXT + AMP + NOR + CTX (80.0%) and SXT + AMP + NOR + CTX + CAZ (66.7%). These findings indicate that the more complex no-inhibition phenotypes in *C. jejuni* were not restricted to a single dominant rep-PCR cluster. A different distribution profile was observed among *C. coli* isolates. The F cluster represented the dominant genetic background for the most frequently detected phenotypes, including NOR (50.0%), NOR + CTX (100%), AMP + NOR (75.0%), SXT + NOR (75.0%), and AMP + NOR + CTX + CAZ (100%). In contrast, several low-frequency phenotypes, including GEN + NOR + CAZ, NOR + CAZ, SXT + CAZ, and SXT + NOR + CTX, were exclusively detected among minor rep-PCR and ND profiles. Overall, these findings further supported species- and genotype-associated clustering patterns among no-inhibition phenotypes, with SXT-associated phenotypes preferentially distributed among the J and minor rep-PCR clusters, whereas simpler NOR-associated phenotypes remained predominantly concentrated within the F cluster.

### 3.3. Distribution of AMR and Virulence-Associated Genes Among Campylobacter Isolates

**Figure 6:**
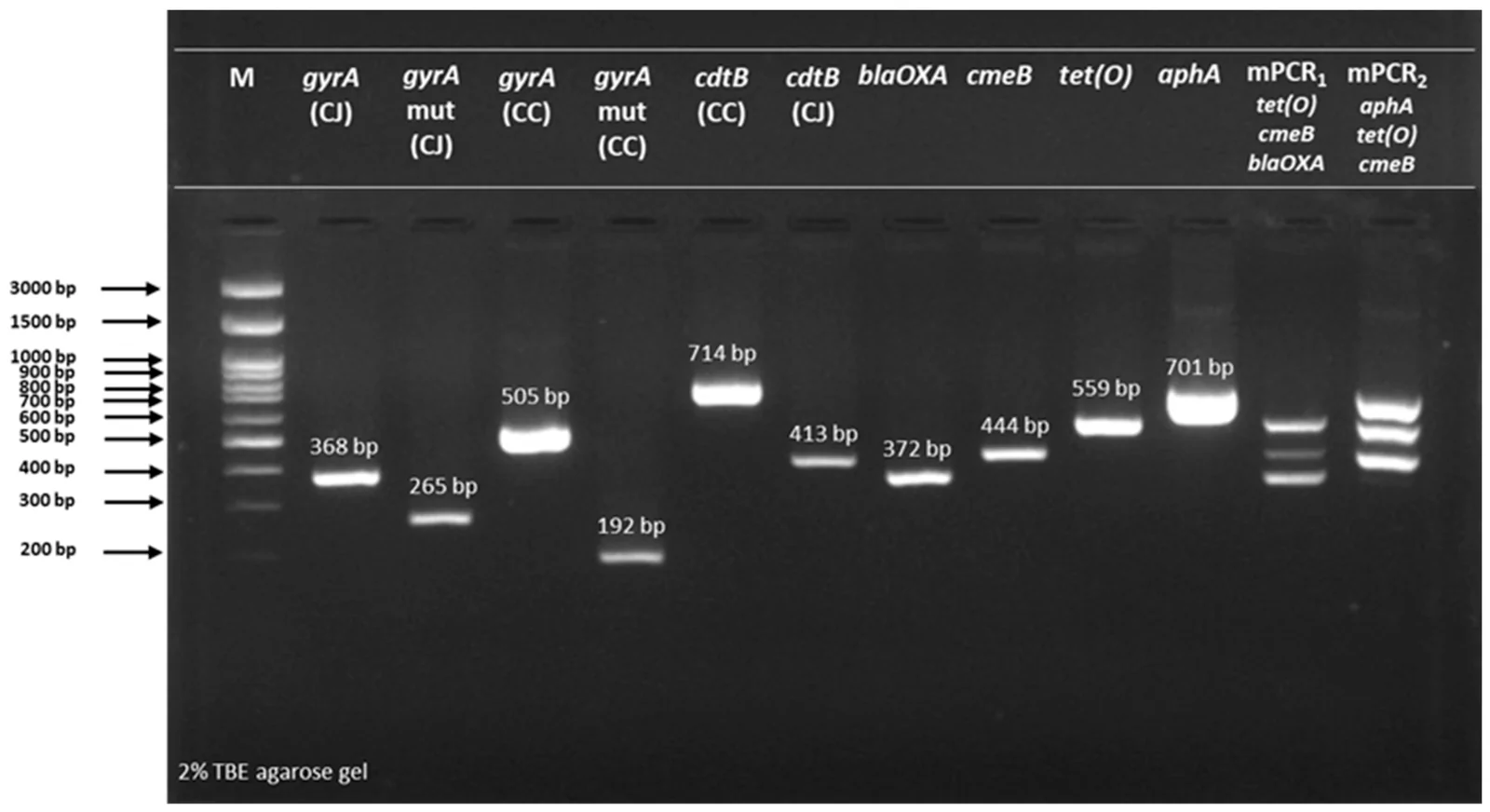
Representative agarose gel electrophoresis of PCR amplicons corresponding to AMR and virulence-associated genes detected among *Campylobacter* isolates. Expected amplicon sizes (bp) are indicated above the corresponding bands. M: DNA molecular weight marker; CJ: *C. jejuni*; CC: *C. coli*; mPCR: multiplex PCR. Lane labels give the target gene, and the species-specific primer set is in parentheses where it applies.

Figure 6 presents representative agarose gel electrophoresis of PCR amplicons corresponding to AMR and virulence-associated genes detected among *Campylobacter* isolates. Expected amplicon sizes for each target gene are indicated above the corresponding bands.

**Figure 7:**
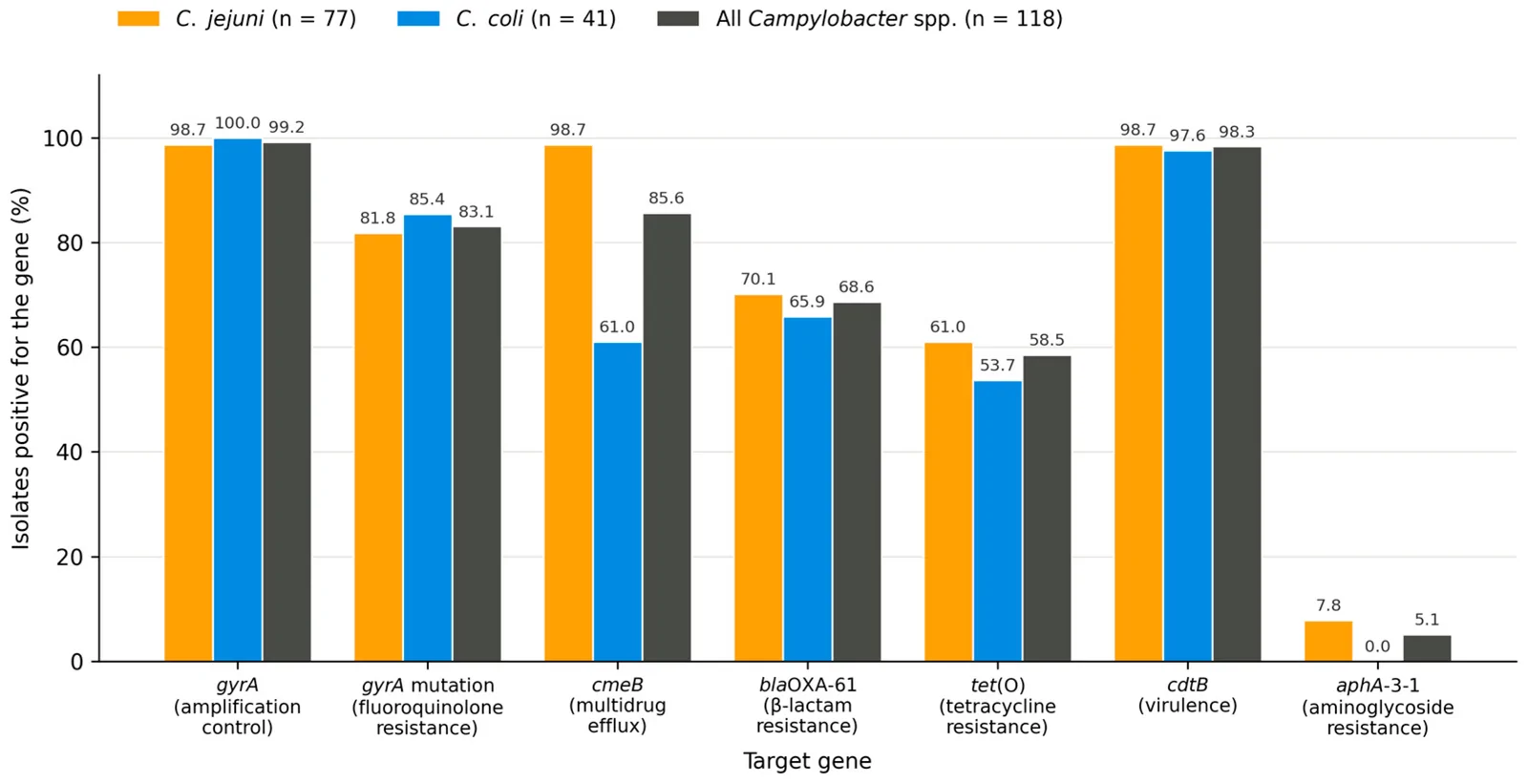
Prevalence of AMR-associated genes (*gyrA* Thr-86-Ile mutation, *bla_OXA-61_*, *tet(O)*, *aphA-3-1*), the multidrug efflux gene *cmeB* and the virulence gene *cdtB* among the 118 isolates with molecular data (two isolates were excluded). AMR: antimicrobial resistance; *gyrA* (wild-type fragment) serves as an internal amplification control. Percentages are relative to the number of isolates of each species (n = 77 and n = 41) or to all genotyped isolates (n = 118).

PCR-based screening showed widespread distribution of AMR and virulence-associated genes among the examined *Campylobacter* isolates (Figure 7). Among the investigated targets, *gyrA* (used as an internal amplification control) and *cdtB* showed the highest prevalence (99.2% and 98.3%, respectively), detected in almost all isolates regardless of species. Similarly, the multidrug efflux-associated gene *cmeB* was highly prevalent in both *C. jejuni* (98.7%) and *C. coli* (61.0%), with a markedly higher prevalence observed among *C. jejuni* isolates. In contrast, the aminoglycoside-associated gene *aphA-3-1* was rarely detected within the collection (detected in only six *C. jejuni* isolates). High prevalence was additionally observed for *gyrA* mutation (83.1%), whereas *bla_OXA-61_* (68.6%) and *tet(O)* (58.5%) showed more variable distribution among the examined isolates. The high prevalence of *gyrA* mutation was consistent with the elevated frequency of CIP-resistant phenotypes previously observed, whereas the presence of *tet(O)* corresponded with the distribution of TE-resistant phenotypes. Overall, despite the pronounced difference observed for *cmeB* prevalence, no major species-dependent differences were observed in the prevalence patterns of the investigated genes, as both *C. jejuni* and *C. coli* exhibited broadly comparable distribution profiles across most targets.

#### Distribution of AMR Determinant Profiles According to Rep-PCR Clustering

Further analysis of AMR determinant profiles by rep-PCR clustering revealed distinct distribution patterns among total *Campylobacter* isolates and between species-specific populations (Table S10). At the total *Campylobacter* spp. level, the predominant resistance determinant profile, consisting of the combined presence of *gyrA* mutation, *bla_OXA-61_*, and *tet(O)*, was broadly distributed across several rep-PCR clusters, with the highest proportions detected within the F (25.0%), J (20.0%), and minor rep-PCR profiles (32.5%). In contrast, simpler determinant profiles showed stronger cluster-specific associations. The *bla_OXA-61_* + *tet(O)* profile was exclusively associated with the F cluster (100%), whereas the *bla_OXA-61_*-only profile was most frequently detected within the J cluster (50.0%). Similarly, the *gyrA* mutation + *tet(O)* profile was most frequently detected in the J cluster (34.8%). Isolates lacking all investigated resistance determinants (“none” profile) were mainly distributed within the J cluster (37.5%). Species-specific analysis demonstrated additional differences between *C. jejuni* and *C. coli*. Among *C. jejuni* isolates, determinant profiles containing *tet(O)* were preferentially distributed within the J cluster, including the *gyrA* mutation + *tet(O)* profile (46.7%) and the *gyrA* mutation + *bla_OXA-61_* + *tet(O)* profile (27.6%). In contrast, the *gyrA* mutation + *bla_OXA-61_* profile was strongly associated with the minor and ND rep-PCR profiles, which accounted for 62.5% of isolates exhibiting this determinant combination. The *bla_OXA-61_*-only profile was predominantly linked to the J cluster (57.1%), while isolates lacking resistance determinants were also mainly distributed within the J cluster (60.0%). A more concentrated distribution pattern was observed among *C. coli* isolates. The F cluster represented the predominant genetic background for nearly all major resistance determinant profiles, including *gyrA* mutation + *bla_OXA-61_* (76.9%), *gyrA* mutation + *bla_OXA-61_* + *tet(O)* (63.6%), and *gyrA* mutation + *tet(O)* (62.5%). Moreover, the *bla_OXA-61_* + *tet(O)* and *gyrA* mutation-only profiles were exclusively detected within the F cluster. In contrast, minor rep-PCR and ND profiles contributed only limited proportions across most determinant combinations. Additional analysis of the multidrug efflux-associated gene *cmeB* by rep-PCR clustering showed cluster-associated distribution patterns among *Campylobacter* isolates. Although *cmeB* was detected in most isolates overall (101/118; 85.6%), *cmeB*-negative isolates were predominantly distributed within the F cluster (10/17; 58.8%) and minor rep-PCR profiles (5/17; 29.4%). Species-specific analysis further showed that *cmeB* was almost universally detected among C. *jejuni* isolates, with only a single *cmeB*-negative isolate identified within the J cluster. In contrast, *cmeB*-negative *C. coli* isolates were mainly concentrated within the F cluster (10/16; 62.5%). These findings further supported species- and cluster-associated distribution patterns previously observed among AMR determinant profiles.

Overall, these findings demonstrated that AMR determinant profiles exhibited cluster-associated distribution patterns. While determinant profiles in *C. jejuni* were generally distributed across multiple rep-PCR backgrounds, indicating greater genetic heterogeneity, the corresponding profiles in *C. coli* were predominantly concentrated within the F cluster, suggesting a more clonal distribution of resistance-associated determinants.

### 3.4. Integrated Association Analyses Among Phenotypic, Genotypic and Ecological Characteristics of Campylobacter Isolates

To better understand relationships among the investigated characteristics of the *Campylobacter* collection, a series of association analyses was performed using the integrated dataset summarized in Table S11. The dataset combines species identification, biofilm-forming capacity under either microaerophilic or aerobic CO_2_-enriched conditions, AMR phenotypes according to EUCAST v.2026 criteria, additional non-inhibition profiles for selected antimicrobials lacking *Campylobacter*-specific interpretive criteria, the presence or absence of AMR and virulence-associated genes, and rep-PCR cluster assignment. This integrated approach enabled the exploration of potential relationships among phenotypic traits (biofilm-related behaviors and AMR profiles) and molecular characteristics, providing further insight into the ecology and adaptive strategies of the studied *Campylobacter* isolates. The aminoglycoside resistance gene *aphA-3-1* was detected in only six of the 118 isolates with available genotypic data (5.1%). Among these isolates, GEN showed measurable inhibition in all cases, and no complete non-inhibition phenotype was detected. Consequently, further genotype-phenotype association analysis was not feasible.

#### 3.4.1. Association Between *gyrA* Mutation Carriage and CIP Resistance Phenotype

The association between *gyrA* mutation carriage and CIP phenotype was evaluated in 118 *Campylobacter* isolates for which both phenotypic and molecular data were available. Among the 98 isolates carrying the *gyrA* mutation, 89 (90.8%) exhibited a CIP-resistant phenotype, whereas 9 (9.2%) were categorized as susceptible, increased exposure (I). In contrast, among the 20 isolates lacking the *gyrA* mutation, 17 (85.0%) were classified as I and only 3 (15.0%) were resistant to CIP. Overall genotype-phenotype concordance reached 89.8% (106/118 isolates). A highly significant association was observed between *gyrA* mutation carriage and CIP phenotype (χ^2^ = 55.6, df = 1, *p* < 0.001; Fisher’s exact test, *p* < 0.001; OR = 56.0, 95% CI: 13.7–229). The 12 discordant isolates comprised 9 *gyrA* mutation-positive isolates categorized as I and 3 *gyrA* mutation-negative isolates that were CIP-resistant. Relative to the CIP-resistant phenotype, the *gyrA* mutation assay showed a sensitivity of 96.7% (89/92), a specificity of 65.4% (17/26), a PPV of 90.8% (89/98) and an NPV of 85.0% (17/20). Notably, no isolate was classified as fully susceptible (S) to CIP under EUCAST v. 2026, so wild-type isolates were categorized as I.

#### 3.4.2. Association Between *tet(O)* Carriage and TE Resistance Phenotype

The association between *tet(O)* carriage and TE resistance phenotype was evaluated in 118 *Campylobacter* isolates for which both phenotypic and molecular data were available. Among the 69 isolates carrying *tet(O)*, 62 (89.9%) exhibited a TE-resistant phenotype, whereas only 7 (10.1%) were categorized as susceptible. In contrast, among the 49 isolates lacking *tet(O)*, 44 (89.8%) were susceptible to TE and only 5 (10.2%) exhibited a resistant phenotype. Overall genotype-phenotype concordance reached 89.8% (106/118). A highly significant association was observed between *tet(O)* carriage and TE resistance phenotype (χ^2^ = 74.1, df = 1, *p* < 0.001; Fisher’s exact test, *p* < 0.001). Furthermore, *tet(O)*-positive isolates had substantially higher odds of expressing TE resistance than *tet(O)*-negative isolates (OR = 77.9, 95% CI: 23.2–262). The 12 discordant isolates comprised 7 *tet(O)*-positive isolates that were TE-susceptible and 5 *tet(O)*-negative isolates that were TE-resistant. Relative to the TE-resistant phenotype, *tet(O)* detection showed a sensitivity of 92.5% (62/67), a specificity of 86.3% (44/51), a PPV of 89.9% (62/69) and an NPV of 89.8% (44/49).

#### 3.4.3. Association Between *bla_OXA-61_* and Absence of Inhibition by AMP

The potential association between the presence of the *bla_OXA-61_* gene and the complete absence of inhibition by AMP was investigated using contingency table analysis. Among the 81 isolates carrying *bla_OXA-61_*, 27 (33.3%) showed no inhibition against AMP, whereas 54 (66.7%) showed measurable inhibition zones. In contrast, among the 37 isolates lacking the gene, only 7 (18.9%) exhibited complete absence of inhibition, while 30 (81.1%) displayed detectable inhibition. Although isolates harboring *bla_OXA-61_* exhibited a higher frequency of complete absence of inhibition than gene-negative isolates (33.3% vs. 18.9%), the observed association was not statistically significant (χ^2^ = 2.57, df = 1, *p* = 0.109). Fisher’s exact test yielded a similar result (*p* = 0.129). The estimated odds ratio was 2.14 (95% CI: 0.834–5.51); however, the confidence interval included unity, indicating that no statistically significant association could be demonstrated in the present dataset. Overall, the results suggest a non-significant tendency towards a higher prevalence of complete absence of inhibition by AMP among *bla_OXA-61_*-positive isolates. However, *bla_OXA-61_* carriage alone did not reliably predict the complete non-inhibition phenotype under the conditions of the present study. It should be noted that this analysis was based on the presence or absence of inhibition zones rather than on established *Campylobacter*-specific clinical susceptibility breakpoints for AMP and therefore represents an exploratory assessment of the relationship between *bla_OXA-61_* carriage and lack of antibacterial activity.

#### 3.4.4. Association Between *cmeB* Carriage and Antimicrobial Response Complexity

Given that *cmeB* is part of an operon encoding a multidrug efflux pump implicated in decreased susceptibility to multiple antimicrobial agents, its association with overall antimicrobial response complexity (resistance to E, TE, or CIP according to EUCAST, plus complete non-inhibition for the other seven antibiotics) was investigated rather than with individual antimicrobial phenotypes alone. For this purpose, isolates were classified according to the number of affected antimicrobial classes. Profiles involving 0–2 antimicrobial classes were categorized as low-complexity, whereas profiles involving 3–5 antimicrobial classes were categorized as high-complexity. Among the 101 *cmeB*-positive isolates, 53 (52.5%) exhibited high-complexity antimicrobial response profiles, compared with only 4 of 17 (23.5%) *cmeB*-negative isolates. A nominal association between *cmeB* carriage and high-complexity profiles was observed (Fisher’s exact test, *p* = 0.035). The odds of exhibiting a high-complexity profile were 3.59 times higher among *cmeB*-positive isolates than among *cmeB*-negative isolates (OR = 3.59, 95% CI: 1.1–11.8). To further explore this relationship, the number of affected antimicrobial classes was also evaluated as a continuous variable. *cmeB*-positive isolates displayed a higher median number of affected classes than *cmeB*-negative isolates (median: 3 vs. 2 classes). However, this difference did not reach statistical significance when assessed using the Mann–Whitney U test (*p* = 0.074). As species was strongly associated with profile complexity (high complexity in 46/77, 59.7%, of *C. jejuni* vs. 11/41, 26.8%, of *C. coli* isolates; Fisher’s exact test, *p* < 0.001; OR = 4.05, 95% CI: 1.77–9.26 for *C. jejuni* vs. *C. coli*) and 16 of the 17 *cmeB*-negative isolates were *C. coli*, the association was also examined within *C. coli*, the only species with a meaningful number of *cmeB*-negative isolates. In *C. coli*, high-complexity profiles were found in 8/25 (32.0%) *cmeB*-positive and 3/16 (18.8%) *cmeB*-negative isolates (OR = 2.04, 95% CI: 0.45–9.2; Fisher’s exact test, *p* = 0.48), with the same median of two affected classes in both groups (*p* = 0.80). The association could not be assessed within *C. jejuni*, as only one isolate lacked *cmeB*. Therefore, the crude association largely reflects species composition, was not consistent across analyses (*p* = 0.035 for the binary comparison vs. *p* = 0.074 for the number of classes), and should be regarded as exploratory. Because this measure combines clinical resistance and non-inhibition, the two components were also analyzed separately. The number of EUCAST-resistant classes (E, TE, CIP) did not differ between *cmeB*-positive and *cmeB*-negative isolates (median 1 vs. 1; Mann–Whitney U test, *p* = 0.73; ≥2 resistant classes in 50/101, 49.5%, vs. 8/17, 47.1%; OR = 1.10; Fisher’s exact test, *p* = 1.00). The number of classes with complete non-inhibition was higher in *cmeB*-positive isolates but not significantly (median 2 vs. 1; *p* = 0.13; ≥2 classes in 69/101, 68.3%, vs. 8/17, 47.1%; OR = 2.43; *p* = 0.10). Thus, the association with the combined measure was driven by non-inhibition phenotypes and not by EUCAST-defined resistance.

### 3.5. Association Between Biofilm Formation and Antimicrobial Response Phenotypes

To test directly whether greater biofilm formation accompanied broader AMR, biofilm formers and non-formers were compared (Table S12). Resistance to at least two EUCAST classes was less frequent in biofilm formers than in non-formers under microaerophilic (18/57, 31.6% vs. 42/63, 66.7%) and aerobic CO_2_-enriched conditions (17/55, 30.9% vs. 43/65, 66.2%), and this persisted after adjustment for species (Mantel–Haenszel OR = 0.23, 95% CI: 0.10–0.53, under both conditions). Formers were also more often susceptible to E, TE and CIP (adjusted OR = 4.22, 95% CI: 1.23–14.5, and 4.64, 95% CI: 1.33–16.1) and less often showed high-complexity profiles (adjusted OR = 0.28, 95% CI: 0.12–0.63, and 0.23, 95% CI: 0.10–0.54). Within *C. jejuni*, A_590_ correlated negatively with the number of resistance classes (Spearman rho = −0.37 and −0.36), whereas no correlation was seen in *C. coli* (rho = −0.02 and −0.05), although the categorical comparisons pointed in the same direction. Thus, greater biofilm formation was not accompanied by broader AMR; if anything, biofilm formers showed less frequent multi-class resistance.

## 4. Discussion

This study used an integrated phenotypic and genotypic approach to investigate in vitro biofilm formation, AMR, resistance- and virulence-associated determinants, and genetic diversity in poultry-derived *C. jejuni* and *C. coli* isolates. Biofilm formation and AMR followed partially distinct patterns: in this collection, they were not positively associated, and their distribution was linked to species identity, genetic background, and, for AMR, specific resistance determinants. However, as this was a cross-sectional correlational study, all relationships described below are associative and do not establish causality.

Biofilm formation was the most heterogeneous phenotype examined. The proportion of biofilm formers was similar under both atmospheres, but atmosphere was associated with isolate-specific shifts toward increased biofilm biomass, without a significant overall difference in A_590_. Thus, atmospheric composition was associated with differences in biofilm biomass rather than with an isolate’s ability to initiate biofilm formation. Previous reports have shown that aerobic exposure can enhance *C. jejuni* biofilm development [51,52], and biofilm development is shaped by both intrinsic properties and environmental stimuli [9]. Because isolates grown under identical conditions differed widely in biofilm-forming capacity, the atmosphere-dependent shifts probably reflect strain-specific physiological responses rather than a universal stimulatory effect of oxygen [11], in line with transcriptomic evidence of coordinated, stage-dependent regulation of oxidative stress and metabolic genes during *C. jejuni* biofilm development [25]. It should be noted that biofilm categories (weak, moderate, or strong) were assigned using condition-specific Ac thresholds (0.78 vs. 0.69), and isolates with A_590_ values close to a category boundary may be reclassified depending on the cut-off applied, a limitation inherent to categorical crystal violet-based schemes. Shifts between weak and moderate categories should therefore be interpreted together with the continuous A_590_ values in Table S3.

Biofilm formation was detected only in MH-CJ; no isolate formed detectable biofilm in MH-HB under either atmosphere. As the same isolates, inocula, plates, and procedures were used, and MH-CJ yielded a wide range of phenotypes, this difference is most likely attributable to the medium rather than the assay. Chicken juice has been reported to promote *Campylobacter* attachment and biofilm formation, possibly through its nutrient content and the conditioning of abiotic surfaces with proteins that favour adhesion [53,54,55], and it better represents the poultry-associated environment from which the isolates originated. In contrast, HB components may favour planktonic growth or coat polystyrene in a way that hinders attachment [56]. In addition, the higher cut-off for MH-HB that was recorded under aerobic conditions (0.99 vs. 0.69) indicates greater background staining that may have reduced sensitivity to weak biofilms. However, these explanations were not tested directly, and the biofilm phenotypes reported here should therefore be interpreted in the context of the MH-CJ model.

The biofilm heterogeneity was not randomly distributed. *C. coli* isolates showed significantly higher biofilm formation than *C. jejuni* under both atmospheres (approximately six-fold higher odds). However, species and rep-PCR cluster were strongly confounded (28/39 F-cluster isolates were *C. coli*, whereas 27/29 J-cluster isolates were *C. jejuni*), so cluster effects were examined with species-adjusted analyses. After adjustment, species remained associated with biofilm formation (adjusted OR ≈ 3.5–3.7), whereas the association with cluster was attenuated: it was not significant under microaerophilic conditions (*p* = 0.11) and weaker under aerobic CO_2_-enriched conditions (*p* = 0.032), where the F cluster still had higher odds of biofilm formation than the other clusters (adjusted OR = 3.40, 95% CI: 1.20–9.66). The weaker biofilm phenotype of the J cluster is largely explained by its C. *jejuni* composition: within *C. jejuni*, the proportion of biofilm formers in the J cluster (37.0% and 29.6%) was close to that of all 78 *C. jejuni* isolates (33.3% and 30.8%; Section 3.1; Table S4). These patterns are consistent with evidence that biofilm-forming capacity differs among genetically distinct strains of the same species [57,58] and that biofilm differences in *C. jejuni* may involve combinations of adhesion-, motility-, and biofilm-related genes rather than a single determinant [24]. However, because some clusters were small and no whole-genome analysis was performed, the adjusted estimates are imprecise, and the association with rep-PCR profiles is compatible with, but does not demonstrate, a link between broader genetic background and biofilm-forming potential.

Although biofilm formation was associated with species and rep-PCR clusters, AMR followed a different pattern. Despite their greater biofilm-forming capacity, *C. coli* isolates did not show a higher AMR burden: the combined TE + CIP phenotype, the most prevalent resistance profile, was more frequent among *C. jejuni* (42/78; 53.8%) than *C. coli* (16/42; 38.1%) isolates, in line with the frequent co-occurrence of TE and fluoroquinolone resistance in poultry-associated *Campylobacter* [59,60]. Our findings therefore do not support a direct relationship between greater biofilm-forming capacity and increased AMR. Indeed, in species-adjusted comparisons, biofilm formers were, if anything, less often resistant to multiple EUCAST classes than non-formers (Table S12). This descriptive inverse association is rather interesting and might suggest that *Campylobacter* isolates inherently susceptible to antimicrobials may favor biofilm formation as an alternative survival mechanism. This contrasts with Zhang et al. [38], who reported higher resistance frequencies in biofilm-producing poultry isolates but also found that biofilm-forming capacity varied among genetic branches, suggesting that this relationship depends on population structure and ecological context [13,24,61]. The absence of a direct positive association does not contradict the recognized role of biofilms in tolerance to antimicrobials; it just indicates that intrinsic biofilm-forming capacity alone does not reliably predict the AMR profile of individual isolates.

AMR was nevertheless not randomly distributed [62,63]. In particular, 77.8% of TE-resistant isolates belonged to the F cluster, which was also the dominant cluster among *C. coli*. This is compatible with a partial association of this phenotype with genetic background in addition to species, in line with genomic evidence that resistance determinants are often associated with particular lineages [64,65] and with the uneven distribution of AMR and biofilm phenotypes among genetic groups reported by Zhang et al. [38]. In contrast, the TE + CIP phenotype was broadly distributed among the F cluster (24.1%), J cluster (24.1%), and minor profiles (25.9%), which may reflect wide dissemination or independent emergence of common mechanisms (*tet(O)* acquisition and *gyrA* mutation [66]) across genetic backgrounds rather than expansion of a single lineage [64,67,68]. These alternatives, however, cannot be distinguished without sequencing-based lineage analysis.

Collectively, in this collection, biofilm formation and AMR were not positively associated and showed different distribution patterns across species and rep-PCR clusters. This does not contradict the role of biofilms in antimicrobial survival, while biofilm-associated responses still depend on strain, species, antimicrobial agent, developmental stage, and environmental conditions [69]. In addition, *Campylobacter* survival may further depend on interactions within microbial communities [18,70]. However, the present data alone cannot determine whether biofilm formation and AMR play complementary roles in persistence. Biofilm capacity was primarily associated with species, with a smaller residual association with the rep-PCR cluster after adjustment, whereas AMR reflected specific resistance mechanisms distributed across genetically diverse backgrounds. This is illustrated by the J cluster, which showed weaker biofilm formation and accounted for 46.7% (7/15) of the fully susceptible isolates (despite representing 24.2% of the collection), but also contributed to the TE + CIP phenotype (14/58 isolates), particularly in *C. jejuni*. As 93.1% of J-cluster isolates were *C. jejuni*, these combinations largely reflect species composition, and species and cluster effects can be only partly separated in the present data (Table S5). Isolates from different backgrounds thus differed in the combinations of traits displayed in vitro. However, these differences are descriptive; persistence was not measured, and they should therefore not be interpreted as evidence of distinct survival strategies or of a reduced capacity for persistence in isolates with lower biofilm formation.

Complete non-inhibition profiles provided an additional perspective on antimicrobial response diversity. NOR non-inhibition was widely distributed in both species, resembling the distribution of CIP resistance, which may reflect shared mechanisms within the fluoroquinolone class, particularly mutations in the *gyrA* quinolone resistance-determining region [66], although non-inhibition is not equivalent to resistance. In contrast, SXT non-inhibition was one of the strongest species-associated phenotypes, being significantly more frequent in *C. jejuni* (73.1%) than in *C. coli* (16.7%) [29,71]. Trimethoprim is generally considered poorly active against *Campylobacter*, so SXT non-inhibition may reflect intrinsic properties and not acquired resistance [72]. The association of SXT-containing phenotypes with the J cluster (e.g., 63.6% of SXT and 45.0% of SXT + NOR isolates) is therefore partly explained by its *C. jejuni* composition; within *C. jejuni*, however, J isolates were still overrepresented among the SXT (60.0%) and SXT + NOR (56.3%) phenotypes relative to their 34.6% share of the species (Table S9). *C. jejuni* isolates were also more broadly distributed across the J cluster and minor rep-PCR profiles, with a wider range of combined non-inhibition patterns, whereas *C. coli* isolates were concentrated in the F cluster, consistent with the greater genetic diversity of *C. jejuni* and the possible clonal expansion of specific *C. coli* lineages [67]. However, it should be noted that the non-inhibition criterion identifies only complete absence of visible antimicrobial activity, should not be equated with resistance, and does not capture partial reductions in susceptibility among isolates with measurable zones (Table S6).

As expected, the *gyrA* mutation and *tet(O)* were strongly associated with CIP and TE resistance, respectively, consistent with their established resistance mechanisms [66,73,74]. Nevertheless, approximately 10% of isolates showed genotype-phenotype discordance for both *gyrA*/CIP and *tet(O)*/TE (12/118 each). Among resistant isolates lacking the marker (3 for CIP; 5 for TE), resistance may be due to mechanisms not targeted here, such as other *gyrA* substitutions (our assay detected only Thr-86-Ile) [45,46,66], other tetracycline resistance determinants or *tet(O)* variants not amplified by the primers used, or enhanced CmeABC efflux activity [68,75]. Among marker-positive susceptible isolates (9 for CIP; 7 for TE), possible explanations include non-functional or poorly expressed alleles, false-positive amplification, or mixed populations. Experimental variability may also have contributed, since disc diffusion zones close to a breakpoint are sensitive to small differences in inoculum, incubation, and reading, and grouping category I with susceptible isolates for CIP is a simplification. As discordant isolates were not confirmed by sequencing or MIC determination, these associations represent predictive concordance between targeted PCR markers and disc diffusion categories, not evidence of the causal mechanism in individual isolates.

In contrast to *gyrA* and *tet(O)*, *cmeB* was not associated with a single phenotype. In the whole collection, *cmeB*-positive isolates showed a nominally higher proportion of complex antimicrobial response profiles, but this association was not independent of species: nearly all *cmeB*-negative isolates were *C. coli* (16/17), a species with less complex profiles overall, and no significant association was found within *C. coli* (OR = 2.04, 95% CI: 0.45–9.2). A role for *cmeB* can nevertheless be considered, since CmeABC is a multidrug efflux pump with a broad substrate range that includes fluoroquinolones, macrolides, tetracyclines, and *β*-lactams [68,75]; however, this study assessed only its presence, not its expression or sequence variants. It is also known that a single pump can reduce susceptibility to structurally unrelated agents, and it may act alongside target-based mechanisms such as *gyrA* mutations or *tet(O)*-mediated ribosomal protection to push isolates beyond inhibition thresholds for more agents. Because *cmeB* was almost universal in *C. jejuni* (98.7%), pump expression and sequence variants [68] probably determine the phenotype, and these were not assessed. The complexity measure combines EUCAST-defined resistance and non-inhibition, and the association was driven by non-inhibition: no association was found with the number of EUCAST-resistant classes. The presence of *cmeB* should therefore not be interpreted as a predictor of resistance to a specific antibiotic or as evidence of an efflux-mediated mechanism, which would require expression analysis or susceptibility testing with an efflux pump inhibitor.

Before concluding, this study’s limitations should be highlighted. As previously stated, its cross-sectional, correlational design allows associations to be described but not causal relationships to be established. All isolates came from retail raw broiler chicken meat purchased in a single town (Myrina, Lemnos Island, Greece) between July and October 2021, and several isolates originated from the same sample. The findings therefore cannot be confidently extrapolated to other regions, seasons, production stages, or food sources. Biofilm formation was assessed in vitro in a single medium (MH-CJ) and does not reflect persistence in food-processing environments. For antibiotics without EUCAST breakpoints, only complete absence of inhibition was recorded, which is not a clinical resistance category. Only the presence of six selected genes was investigated, a limited panel that cannot fully explain the observed phenotypes (for example, no macrolide resistance determinants were tested), and not their expression or sequence variants. Discordant genotype-phenotype results were not confirmed by sequencing or MIC determination. Species and rep-PCR clusters were partly confounded; this was addressed for biofilm formation by species-adjusted analyses whose precision was limited by the small size of some clusters, whereas the other cluster-based associations were univariate. All analyses were exploratory and not adjusted for multiple comparisons. In addition, rep-PCR has lower discriminatory power and reproducibility than MLST or whole-genome sequencing. Its clusters group isolates by fingerprint similarity and cannot be equated with sequence types, clonal complexes, or phylogenetic lineages; the method also cannot resolve recombination or horizontal gene transfer or identify the genetic determinants underlying the observed phenotypes. Higher-resolution genomic approaches are therefore needed to define the population structure behind the observed associations. The conclusions should thus be read as associations specific to this collection, to be tested in larger and broader collections using genomic and functional approaches.

Overall, biofilm formation was mainly associated with species, with a weaker, atmosphere-dependent association with rep-PCR cluster after adjustment for species, and was not positively associated with AMR (biofilm formers were, if anything, less often multi-resistant), whereas AMR profiles reflected specific resistance determinants distributed across genetically diverse backgrounds. The lack of a direct relationship between greater biofilm formation and increased AMR suggests that, in this collection, these traits did not behave as components of a single coordinated phenotype. From a practical standpoint, these findings indicate that, in poultry-derived *Campylobacter*, AMR (notably the high TE and CIP resistance) and in vitro biofilm-forming capacity should be assessed separately, and that species identity should be considered when interpreting both, so that surveillance and control strategies in the poultry food chain do not rely on one trait to infer the other.

## Figures and Tables

**Table 1:** Antibiotics tested and EUCAST interpretive criteria used for *C. jejuni* and *C. coli* isolates.

Antibiotic	Class	Disk	Zone Diameter		Reference
Agent ^1^		Content	Breakpoint (mm)		
			S≥	R<	
E (*C. jejuni*)	Macrolides	15 μg	20	20	EUCAST ^2^
E (*C. coli*)	Macrolides	15 μg	18	18	EUCAST
TE	Tetracyclines	30 μg	30	30	EUCAST
GEN	Aminoglycosides	10 μg			No EUCAST breakpoint
SXT	Miscellaneous agents	25 μg			No EUCAST breakpoint
AMP	Penicillins	10 μg			No EUCAST breakpoint
CIP	Fluoroquinolones	5 μg	50	26	EUCAST
NOR	Fluoroquinolones	10 μg			No EUCAST breakpoint
MEM	Carbapenems	10 μg			No EUCAST breakpoint
CTX	Cephalosporins	5 μg			No EUCAST breakpoint
CAZ	Cephalosporins	10 μg			No EUCAST breakpoint

^1^ E: Erythromycin; TE: Tetracycline; GEN: Gentamicin; SXT: Trimethoprim-sulfamethoxazole 1:19; AMP: Ampicillin; CIP: Ciprofloxacin; NOR: Norfloxacin; MEM: Meropenem; CTX: Cefotaxime; CAZ: Ceftazidime.^2^ EUCAST Clinical Breakpoint Tables v. 16.0, valid from 1 January 2026 [42].

**Table 2:** Heatmap representation of the distribution (%) of non- (N), weak (W), moderate (M), and strong (S) biofilm-forming phenotypes across the major rep-PCR clusters of *Campylobacter* isolates (n = 120) under either microaerophilic (6.2–13.2% O_2_, 2.5–9.5% CO_2_) or aerobic CO_2_-enriched conditions (15% O_2_, 3.5–9.0% CO_2_) after 48 h incubation in MH–CJ medium at 42 °C. Minor profiles include rep-PCR clusters represented by fewer than ten isolates. Biofilm categories were assigned using condition-specific cut-offs (Ac = 0.78 and 0.69 for microaerophilic and aerobic CO_2_-enriched conditions, respectively; weak: Ac < A_590_ ≤ 2 × Ac; moderate: 2 × Ac < A590 ≤ 4 × Ac; strong: A_590_ > 4 × Ac; see Table S3).

Rep-PCR		Microaerophilic Conditions				Aerobic CO_2_-Enriched Conditions			
Cluster		N	W	M	S	N	W	M	S
F	n = 39	25.6%	20.5%	20.5%	33.3%	23.1%	12.8%	23.1%	41.0%
H	n = 15	60.0%	0.0%	6.7%	33.3%	60.0%	0.0%	13.3%	26.7%
J	n = 29	62.1%	20.7%	0.0%	17.2%	69.0%	3.4%	10.3%	17.2%
Minor profiles	n = 24	62.5%	0.0%	0.0%	37.5%	66.7%	0.0%	8.3%	25.0%
ND	n = 13	84.6%	7.7%	0.0%	7.7%	84.6%	0.0%	7.7%	7.7%

ND: rep-PCR profile not determined.

**Table 3:** Classification of the 120 *Campylobacter* spp. isolates using different EUCAST clinical breakpoint versions (2013, 2021, and 2026).

Antibiotic	Number of Isolates *,**								
	EUCAST 2013			EUCAST 2021			EUCAST 2026		
	S	I	R	S	I	R	S	I	R
E	116	0	4	116	0	4	117	0	3
TE	51	0	69	51	0	69	51	0	69
CIP	26	0	94	0	26	94	0	26	94

S: Susceptible, I: Susceptible, increased exposure, R: Resistant. * The same dataset (n = 120 isolates) was interpreted using different versions of EUCAST clinical breakpoints. ** Differences in susceptibility classification reflect breakpoint revisions rather than changes in inhibition zone measurements.

## Data Availability

The data presented in this study are contained within the article or Supplementary Materials.

## Supplementary Materials

The following supporting information can be downloaded at https://www.mdpi.com/article/10.3390/foods15213815/s1, Table S1: Distribution of *C. jejuni* and *C. coli* chicken meat isolates and their rep-PCR clusters, as previously determined [39]. ND indicates isolates for which rep-PCR clustering could not be determined due to insufficient DNA quantity (n = 13). The reference strain *C. jejuni* ATCC 33291 was included as a control; Table S2: Primers and PCR targets used for the detection of AMR and virulence-associated genes in *C. jejuni* and *C. coli* isolates [45,46,47,48,49,50]; Table S3: Biofilm biomass (A_590_) for the 120 *Campylobacter* isolates and classification of each isolate as a non-biofilm former (Z), weak (W), moderate (M), or strong biofilm former (S). Each isolate was incubated in MH-CJ or MH-HB broth at 42 °C for 48 h under either microaerophilic or aerobic CO_2_-enriched conditions to allow biofilm formation. Biofilm absorbance data are presented as mean ± standard deviation. Cut-off absorbance (Ac) values and the corresponding thresholds used for biofilm classification under each growth condition are also provided; Table S4: Heatmap representation of species-specific biofilm-forming phenotypes among the major rep-PCR clusters of *C. jejuni* (up) and *C. coli* (down) isolates under either microaerophilic or aerobic CO_2_-enriched conditions after 48 h incubation in MH-CJ medium at 42 °C. Percentages indicate the distribution of non- (N), weak (W), moderate (M), and strong (S) biofilm-forming phenotypes within each rep-PCR group. Minor profiles include rep-PCR clusters represented by fewer than ten isolates; Table S5: Species-adjusted association between rep-PCR cluster and biofilm formation (Firth logistic regression; n = 107 isolates with assigned cluster). Biofilm formation (yes/no; A_590_ > Ac) in MH-CJ medium was modelled separately for each atmospheric condition, with species (*C. coli* vs. *C. jejuni*) and rep-PCR cluster (H, J and minor profiles vs. F) as predictors. The 13 isolates without an assigned rep-PCR cluster (ND) were excluded; Table S6. Inhibition zone diameters (mm) for the 10 antibiotics (E, TE, GEN, SXT, AMP, CIP, NOR, MEM, CTX, CAZ) against the 120 *Campylobacter* isolates, as determined by the disk diffusion method. The inhibition zone diameters against the reference strain *C. jejuni* ATCC 33291, along with the summary of inhibition zone diameters per antibiotic and species, are also presented; Table S7: Heatmap-based visualization of combined no-inhibition phenotypes among *C. jejuni* and *C. coli* isolates for antibiotics lacking established EUCAST clinical breakpoints (GEN, AMP, SXT, NOR, MEM, CAZ, and CTX). No-inhibition phenotypes describe the absence of a visible inhibition zone and are not resistance categories. Phenotypes were grouped by shared antibiotic combinations and overall distribution trends. The left heatmap (Prevalences within species) shows the frequency (%) of each no-inhibition phenotype relative to the total number of isolates within each species (*C. jejuni*, n = 78; *C. coli*, n = 42) and within the total isolate collection (n = 120). The right heatmap (Species contribution within phenotype) shows the proportional contribution (%) of each species within a given no-inhibition phenotype. The column “n” indicates the total number of isolates assigned to each phenotype. Absence of visible inhibition was recorded as an inhibition zone diameter of 1 mm; Table S8: Heatmap representation of AMR phenotypes across the major rep-PCR clusters of the total *Campylobacter* collection (top), *C. jejuni* (middle), and *C. coli* (bottom). Heatmap values represent the distribution (%) of rep-PCR clusters within each AMR phenotype. The rightmost column indicates the overall prevalence (%) of each rep-PCR cluster in the corresponding isolate collection. Minor profiles include rep-PCR clusters represented by fewer than ten isolates; Table S9: Heatmap representation of combined no-inhibition phenotypes across the major rep-PCR clusters of the total *Campylobacter* collection (top), *C. jejuni* (middle), and *C. coli* (bottom). No-inhibition phenotypes describe the absence of a visible inhibition zone and are not resistance categories. Heatmap values represent the distribution (%) of each no-inhibition phenotype within the corresponding rep-PCR cluster. The rightmost column indicates the overall prevalence (%) of each phenotype in the respective isolate collection. Minor profiles include rep-PCR clusters represented by fewer than ten isolates. Absence of visible inhibition was recorded as an inhibition zone diameter of 1 mm; Table S10: Distribution of AMR determinant profiles across rep-PCR clusters among total *Campylobacter* isolates, *C. jejuni*, and *C. coli* populations. Heatmap values represent the row-normalized distribution (%) of each rep-PCR cluster within the corresponding AMR determinant profile. Determinant profiles were defined according to the presence/absence combinations of *gyrA* mutation, *bla_OXA-61_*, and *tet(O)* resistance determinants. Values in the “Total isolates (n)” column indicate the number of isolates exhibiting each determinant profile. “None” refers to isolates lacking all AMR determinants investigated in this analysis. Minor profiles include rep-PCR clusters represented by fewer than 10 isolates and therefore grouped together for visualization purposes. ND indicates isolates for which rep-PCR clustering was not determined due to insufficient DNA quantity; Table S11: Phenotypic and genotypic characteristics of the 120 *Campylobacter* isolates and the reference strain ATCC 33291 included in this study. The table summarizes species assignment, biofilm formation under either microaerophilic or aerobic CO_2_-enriched conditions, biofilm condition profile, antibiotic resistance profile according to EUCAST 2026 breakpoints for *C. jejuni* and *C. coli*, additional non-inhibition profiles (no visible inhibition zone) for antibiotics lacking *Campylobacter*-specific EUCAST interpretation, and the presence/absence of selected AMR and virulence-associated genes; Table S12. Association between biofilm formation and antimicrobial response phenotypes among the 120 *Campylobacter* isolates. Biofilm formation (yes/no; A_590_ > Ac) in MH-CJ medium was analysed separately for each atmospheric condition and compared with the number of EUCAST-resistant classes (E, TE, CIP) and with antimicrobial response complexity, overall and within each species.

## Abbreviations

The following abbreviations are used in this manuscript:

A_590_	Absorbance at 590 nm
Ac	Cut-off absorbance
AMR	Antimicrobial resistance
AMP	Ampicillin
AST	Antimicrobial susceptibility testing
ATCC	American Type Culture Collection
CAZ	Ceftazidime
CBA	Campylobacter blood agar
CFU	Colony-forming units
CI	Confidence interval
CIP	Ciprofloxacin
CTX	Cefotaxime
CV	Crystal violet
DNA	Deoxyribonucleic acid
E	Erythromycin
ECDC	European Centre for Disease Prevention and Control
EFSA	European Food Safety Authority
ERDF	European Regional Development Fund
EtBr EU	Ethidium bromide European Union
EUCAST	European Committee on Antimicrobial Susceptibility Testing
gDNA	Genomic DNA
GEN	Gentamicin
HB	Horse blood
HGT	Horizontal gene transfer
MEM	Meropenem
MH	Mueller–Hinton
MH-CJ	Mueller–Hinton broth supplemented with chicken juice
MH-HB	Mueller–Hinton broth supplemented with horse blood
MLST	Multilocus sequence typing
mPCR	Multiplex polymerase chain reaction
NAD	*β*-Nicotinamide adenine dinucleotide
NC	Negative control
ND	Not determined
NOR	Norfloxacin
OD_600_	Optical density at 600 nm
OR	Odds ratio
PCR	Polymerase chain reaction
PS	Polystyrene
rep-PCR	Repetitive sequence-based polymerase chain reaction
SXT	Trimethoprim-sulfamethoxazole
TBE	Tris-borate-EDTA
TE	Tetracycline
UV	Ultraviolet
