# Supplementary material for: Integrated Phenotypic and Genotypic Analysis Reveals Distinct Patterns of Biofilm Formation and Antimicrobial Resistance in Poultry-Derived Campylobacter jejuni and Campylobacter coli
Source: Foods. 2026 Oct 25;15(21):3815. doi: 10.3390/foods15213815 (PMC13763043; doi:10.3390/foods15213815)
Supplement: Supplementary file 1 [file foods-15-03815-s001.zip › Supplementary Table S1.pdf]

Table S1. Distribution of *C. jejuni* and *C. coli* chicken meat isolates and their rep-PCR clusters, as previously determined [39]. ND indicates isolates for which rep-PCR clustering could not be determined due to insufficient DNA quantity (n = 13). The reference strain *C. jejuni* ATCC 33291 was included as a control.

s/n	Isolation ID	Species	Rep-PCR cluster
	ATCC 33291	*C. jejuni*	J
1	POUL007/CAMP001	*C. jejuni*	D
2	POUL010/CAMP003	*C. jejuni*	J
3	POUL007/CAMP004	*C. coli*	F
4	POUL007/CAMP005	*C. coli*	F
5	POUL008/CAMP006	*C. jejuni*	E
6	POUL009/CAMP007	*C. jejuni*	E
7	POUL009/CAMP008	*C. jejuni*	D
8	POUL010/CAMP009	*C. jejuni*	J
9	POUL010/CAMP010	*C. coli*	F
10	POUL012/CAMP011	*C. jejuni*	ND
11	POUL012/CAMP012	*C. jejuni*	J
12	POUL012/CAMP013	*C. jejuni*	J
13	POUL015/CAMP014	*C. coli*	F
14	POUL015/CAMP015	*C. coli*	F
15	POUL015/CAMP016	*C. coli*	F
16	POUL015/CAMP017	*C. coli*	F
17	POUL011/CAMP018	*C. jejuni*	H
18	POUL012/CAMP019	*C. jejuni*	F
19	POUL014/CAMP020	*C. jejuni*	F
20	POUL015/CAMP021	*C. coli*	F
21	POUL011/CAMP022	*C. jejuni*	H
22	POUL012/CAMP023	*C. jejuni*	G
23	POUL015/CAMP024	*C. jejuni*	F
24	POUL016/CAMP025	*C. coli*	F
25	POUL016/CAMP026	*C. coli*	F
26	POUL016/CAMP027	*C. jejuni*	G
27	POUL016/CAMP028	*C. jejuni*	I
28	POUL016/CAMP029	*C. jejuni*	H
29	POUL019/CAMP030	*C. coli*	H
30	POUL016/CAMP031	*C. coli*	F
31	POUL017/CAMP032	*C. coli*	I
32	POUL017/CAMP033	*C. coli*	F
33	POUL017/CAMP034	*C. coli*	F
34	POUL019/CAMP035	*C. jejuni*	C
35	POUL019/CAMP036	*C. jejuni*	G
36	POUL025/CAMP037	*C. jejuni*	C
37	POUL021/CAMP038	*C. coli*	I
38	POUL022/CAMP039	*C. coli*	F
39	POUL023/CAMP040	*C. jejuni*	E
40	POUL024/CAMP041	*C. jejuni*	A

41	POUL025/CAMP042	*C. coli*	F
42	POUL026/CAMP043	*C. jejuni*	H
43	POUL025/CAMP044	*C. coli*	F
44	POUL026/CAMP045	*C. jejuni*	H
45	POUL028/CAMP046	*C. jejuni*	I
46	POUL027/CAMP048	*C. jejuni*	H
47	POUL028/CAMP049	*C. jejuni*	I
48	POUL029/CAMP050	*C. jejuni*	J
49	POUL030/CAMP051	*C. jejuni*	J
50	POUL028/CAMP052	*C. jejuni*	I
51	POUL029/CAMP053	*C. jejuni*	J
52	POUL031/CAMP054	*C. jejuni*	I
53	POUL032/CAMP056	*C. coli*	F
54	POUL032/CAMP057	*C. coli*	F
55	POUL032/CAMP058	*C. jejuni*	D
56	POUL033/CAMP059	*C. jejuni*	J
57	POUL033/CAMP062	*C. jejuni*	J
58	POUL036/CAMP063	*C. jejuni*	ND
59	POUL037/CAMP064	*C. jejuni*	J
60	POUL037/CAMP065	*C. jejuni*	F
61	POUL038/CAMP066	*C. jejuni*	J
62	POUL038/CAMP067	*C. jejuni*	J
63	POUL041/CAMP068	*C. jejuni*	F
64	POUL037/CAMP069	*C. jejuni*	H
65	POUL040/CAMP070	*C. jejuni*	F
66	POUL041/CAMP071	*C. coli*	F
67	POUL037/CAMP072	*C. coli*	H
68	POUL037/CAMP073	*C. jejuni*	H
69	POUL038/CAMP074	*C. jejuni*	J
70	POUL038/CAMP075	*C. jejuni*	J
71	POUL040/CAMP076	*C. jejuni*	F
72	POUL041/CAMP077	*C. jejuni*	J
73	POUL041/CAMP078	*C. jejuni*	F
74	POUL045/CAMP080	*C. coli*	F
75	POUL045/CAMP081	*C. coli*	F
76	POUL044/CAMP082	*C. coli*	F
77	POUL044/CAMP083	*C. coli*	J
78	POUL042/CAMP084	*C. jejuni*	ND
79	POUL042/CAMP085	*C. jejuni*	ND
80	POUL043/CAMP086	*C. jejuni*	F
81	POUL043/CAMP087	*C. jejuni*	J
82	POUL044/CAMP088	*C. jejuni*	ND
83	POUL044/CAMP089	*C. coli*	ND
84	POUL044/CAMP090	*C. coli*	ND
85	POUL045/CAMP091	*C. jejuni*	J
86	POUL045/CAMP092	*C. coli*	F
87	POUL046/CAMP093	*C. jejuni*	ND
88	POUL046/CAMP094	*C. jejuni*	I

89	POUL047/CAMP095	*C. coli*	ND
90	POUL048/CAMP096	*C. coli*	F
91	POUL047/CAMP097	*C. coli*	F
92	POUL048/CAMP098	*C. coli*	B
93	POUL048/CAMP099	*C. coli*	J
94	POUL049/CAMP100	*C. jejuni*	J
95	POUL048/CAMP102	*C. coli*	F
96	POUL048/CAMP103	*C. jejuni*	J
97	POUL048/CAMP104	*C. jejuni*	ND
98	POUL048/CAMP105	*C. jejuni*	H
99	POUL048/CAMP106	*C. jejuni*	F
100	POUL053/CAMP107	*C. coli*	D
101	POUL055/CAMP108	*C. jejuni*	J
102	POUL056/CAMP109	*C. jejuni*	J
103	POUL053/CAMP110	*C. coli*	I
104	POUL055/CAMP111	*C. jejuni*	H
105	POUL056/CAMP112	*C. jejuni*	J
106	POUL056/CAMP113	*C. jejuni*	H
107	POUL055/CAMP114	*C. jejuni*	J
108	POUL053/CAMP117	*C. jejuni*	ND
109	POUL057/CAMP119	*C. coli*	ND
110	POUL057/CAMP121	*C. coli*	F
111	POUL058/CAMP122	*C. coli*	ND
112	POUL060/CAMP123	*C. jejuni*	J
113	POUL060/CAMP124	*C. jejuni*	H
114	POUL061/CAMP125	*C. jejuni*	J
115	POUL062/CAMP126	*C. jejuni*	J
116	POUL063/CAMP127	*C. coli*	F
117	POUL062/CAMP129	*C. jejuni*	D
118	POUL062/CAMP132	*C. jejuni*	J
119	POUL064/CAMP130	*C. jejuni*	F
120	POUL061/CAMP131	*C. jejuni*	H
