# Supplementary material for: Integrated Phenotypic and Genotypic Analysis Reveals Distinct Patterns of Biofilm Formation and Antimicrobial Resistance in Poultry-Derived Campylobacter jejuni and Campylobacter coli
Source: Foods. 2026 Oct 25;15(21):3815. doi: 10.3390/foods15213815 (PMC13763043; doi:10.3390/foods15213815)
Supplement: Supplementary file 1 [file foods-15-03815-s001.zip › Supplementary Table S10.pdf]

Table S10. Distribution of AMR determinant profiles across rep-PCR clusters among total *Campylobacter* isolates, *C. jejuni*, and *C. coli* populations. Heatmap values represent the row-normalized distribution (%) of each rep-PCR cluster within the corresponding AMR determinant profile. Determinant profiles were defined according to the presence/absence combinations of *gyrA* mutation, *bla*$_{OXA-61}$, and *tet(O)* resistance determinants. Values in the "Total isolates (n)" column indicate the number of isolates exhibiting each determinant profile. "None" refers to isolates lacking all AMR determinants investigated in this analysis. Minor profiles include rep-PCR clusters represented by fewer than 10 isolates and therefore grouped together for visualization purposes. ND indicates isolates for which rep-PCR clustering was not determined due to insufficient DNA quantity.

**Resistant determinant profile**	**Total isolates (n)**	**rep-PCR cluster distribution within determinant profile (%)**				
**(Total *Campylobacter* isolates)**		**F (n = 39)**	**H (n = 15)**	**J (n = 29)**	**Minor profiles (n = 24)**	**ND (n = 11)**
none	8	25.0%	25.0%	**37.5%**	12.5%	0.0%
bla$_{OXA-61}$ only	8	25.0%	12.5%	**50.0%**	0.0%	12.5%
gyrA mutation only	6	**50.0%**	0.0%	33.3%	16.7%	0.0%
bla$_{OXA-61}$ + *tet(O)*	4	**100.0%**	0.0%	0.0%	0.0%	0.0%
gyrA mutation + *bla*$_{OXA-61}$	29	**41.4%**	3.4%	13.8%	20.7%	20.7%
gyrA mutation + *tet(O)*	23	26.1%	17.4%	**34.8%**	13.0%	8.7%
gyrA mutation + *bla*$_{OXA-61}$ + *tet(O)*	40	25.0%	17.5%	20.0%	**32.5%**	5.0%

**(*C. jejuni* isolates)**		**F (n = 11)**	**H (n = 13)**	**J (n = 27)**	**Minor & ND profiles (n = 26)**
none	5	20.0%	20.0%	**60.0%**	0.0%
bla$_{OXA-61}$ only	7	28.6%	14.3%	**57.1%**	0.0%
gyrA mutation only	3	0.0%	0.0%	**66.7%**	33.3%
bla$_{OXA-61}$ + *tet(O)*	2	**100.0%**	0.0%	0.0%	0.0%
gyrA mutation + *bla*$_{OXA-61}$	16	12.5%	6.3%	18.8%	**62.5%**
gyrA mutation + *tet(O)*	15	6.7%	26.7%	**46.7%**	20.0%
gyrA mutation + *bla*$_{OXA-61}$ + *tet(O)*	29	10.3%	20.7%	27.6%	**41.4%**

**(*C. coli* isolates)**		**F (n = 28)**	**Minor & ND profiles (n = 13)**

none	3	33.3%	33.3%
bla$_{OXA-61}$ only	1	0.0%	0.0%
gyrA mutation only	3	**100.0%**	0.0%
bla$_{OXA-61}$ + *tet(O)*	2	**100.0%**	0.0%
gyrA mutation + *bla*$_{OXA-61}$	13	**76.9%**	0.0%
gyrA mutation + *tet(O)*	8	**62.5%**	12.5%
gyrA mutation + *bla*$_{OXA-61}$ + *tet(O)*	11	**63.6%**	27.3%
