# Supplementary material for: Integrated Phenotypic and Genotypic Analysis Reveals Distinct Patterns of Biofilm Formation and Antimicrobial Resistance in Poultry-Derived Campylobacter jejuni and Campylobacter coli
Source: Foods. 2026 Oct 25;15(21):3815. doi: 10.3390/foods15213815 (PMC13763043; doi:10.3390/foods15213815)
Supplement: Supplementary file 1 [file foods-15-03815-s001.zip › Supplementary Table S11.pdf]

Table S11. Phenotypic and genotypic characteristics of the 120 *Campylobacter* isolates and the reference strain ATCC 33291 included in this study. The table summarizes species assignment, biofilm formation under either microaerophilic or aerobic CO_2-enriched conditions, biofilm condition profile, antibiotic resistance profile according to EUCAST 2026 breakpoints for *C. jejuni* and *C. coli*, additional non-inhibition profiles (no visible inhibition zone) for antibiotics lacking *Campylobacter*-specific EUCAST interpretation, and the presence/absence of selected AMR and virulence-associated genes.

s/n	Isolation ID	Species	Biofilm formation: Strong (S); Moderate (M); Weak (W); Non-producer (N)		Biofilm formation: Condition profile	Antibiotic resistance profile (EUCAST 2026)*	Additional non-inhibition profile (no visible inhibition zone)**	Virulence- and resistance-associated genes							Rep-PCR clusters
			Microaerophilic (M)	Aerobic (A)				*gyrA*	*gyrA* mutation	*bla* $_{OXA-61}$	*tet(O)*	*cmeB*	*aphA-3-1*	*cdtB*	
	ATCC 33291	*C. jejuni*	N	N	none	none	none	+	-	+	-	+	-	+	**J**
1	POUL007/CAMP001	*C. jejuni*	N	N	none	TE, CIP	AMP, NOR	+	+	+	+	+	-	+	D
2	POUL010/CAMP003	*C. jejuni*	N	N	none	CIP	SXT, NOR, CTX	+	+	-	-	+	-	+	**J**
3	POUL007/CAMP004	*C. coli*	**W**	**M**	**AM**	TE, CIP	NOR, CTX	+	+	-	+	+	-	+	F
4	POUL007/CAMP005	*C. coli*	**S**	**S**	**AM**	TE, CIP	NOR, CTX	+	+	-	+	+	-	+	F
5	POUL008/CAMP006	*C. jejuni*	N	N	none	TE, CIP	AMP, NOR	+	+	+	+	+	-	+	E
6	POUL009/CAMP007	*C. jejuni*	N	N	none	TE, CIP	SXT, AMP, NOR, CTX	+	+	+	+	+	-	+	E
7	POUL009/CAMP008	*C. jejuni*	N	N	none	TE, CIP	SXT, AMP, NOR, CAZ	+	+	+	+	+	-	+	D
8	POUL010/CAMP009	*C. jejuni*	N	N	none	CIP	SXT, NOR	+	+	-	-	+	-	+	**J**
9	POUL010/CAMP010	*C. coli*	N	**S**	A	TE	none	+	+	-	-	+	-	+	F
10	POUL012/CAMP011	*C. jejuni*	N	N	none	TE, CIP	AMP, NOR	+	+	+	+	+	-	+	ND
11	POUL012/CAMP012	*C. jejuni*	N	N	none	TE, CIP	AMP, NOR	+	+	+	+	+	-	+	**J**
12	POUL012/CAMP013	*C. jejuni*	N	N	none	TE, CIP	AMP, NOR	+	+	+	+	+	-	+	**J**
13	POUL015/CAMP014	*C. coli*	**M**	**M**	**AM**	TE	none	+	-	+	+	+	-	+	F
14	POUL015/CAMP015	*C. coli*	**M**	**W**	**AM**	TE	none	+	+	+	+	+	-	+	F
15	POUL015/CAMP016	*C. coli*	**W**	**M**	**AM**	TE	none	+	+	+	+	+	-	+	F
16	POUL015/CAMP017	*C. coli*	**M**	**W**	**AM**	TE	none	+	+	+	+	+	-	+	F
17	POUL011/CAMP018	*C. jejuni*	**S**	**S**	**AM**	TE, CIP	SXT, NOR, CTX, CAZ	+	+	+	+	+	-	+	H
18	POUL012/CAMP019	*C. jejuni*	N	N	none	TE, CIP	AMP, NOR	+	+	+	+	+	-	+	F
19	POUL014/CAMP020	*C. jejuni*	N	N	none	TE, CIP	NOR	+	+	+	+	+	-	+	F
20	POUL015/CAMP021	*C. coli*	**W**	**W**	**AM**	TE	none	+	-	+	+	+	-	+	F
21	POUL011/CAMP022	*C. jejuni*	N	**S**	A	TE, CIP	SXT, NOR, CTX, CAZ	+	+	+	+	+	-	+	H
22	POUL012/CAMP023	*C. jejuni*	**S**	N	M	TE, CIP	SXT, AMP, NOR, CTX	+	+	+	+	+	-	+	G
23	POUL015/CAMP024	*C. jejuni*	**M**	**M**	**AM**	TE, CIP	SXT, AMP, NOR	+	+	+	+	+	-	+	F
24	POUL016/CAMP025	*C. coli*	**M**	**M**	**AM**	CIP	AMP, NOR, CTX, CAZ	+	+	+	+	+	-	+	F
25	POUL016/CAMP026	*C. coli*	**M**	**M**	**AM**	CIP	AMP, NOR	+	+	+	-	+	-	+	F
26	POUL016/CAMP027	*C. jejuni*	N	N	none	TE, CIP	SXT, NOR, CAZ	+	+	-	+	+	-	+	G
27	POUL016/CAMP028	*C. jejuni*	N	N	none	TE, CIP	NOR	+	+	-	+	+	+	+	I
28	POUL016/CAMP029	*C. jejuni*	N	N	none	TE, CIP	SXT, AMP, NOR	+	+	+	+	+	-	+	H
29	POUL019/CAMP030	*C. coli*	N	N	none	TE, CIP	SXT, NOR	+	+	+	+	+	-	-	H
30	POUL016/CAMP031	*C. coli*	**W**	**M**	**AM**	CIP	AMP, NOR, CTX, CAZ	+	+	+	-	+	-	+	F
31	POUL017/CAMP032	*C. coli*	**S**	**S**	**AM**	CIP	NOR	+	+	-	+	-	-	+	I
32	POUL017/CAMP033	*C. coli*	**W**	**M**	**AM**	CIP	NOR, CTX	+	+	-	-	+	-	+	F
33	POUL017/CAMP034	*C. coli*	**S**	**S**	**AM**	TE, CIP	NOR	+	+	-	+	-	-	+	F
34	POUL019/CAMP035	*C. jejuni*	N	N	none	TE, CIP	NOR	+	+	+	+	+	-	+	C
35	POUL019/CAMP036	*C. jejuni*	N	N	none	TE, CIP	SXT, NOR	+	+	+	+	+	-	+	G
36	POUL025/CAMP037	*C. jejuni*	N	N	none	TE, CIP	NOR, CTX	+	+	+	+	+	-	+	C
37	POUL021/CAMP038	*C. coli*	**S**	**S**	**AM**	TE, CIP	NOR	+	+	+	+	-	-	+	I
38	POUL022/CAMP039	*C. coli*	**M**	**S**	**AM**	CIP	NOR	+	+	-	-	+	-	+	F
39	POUL023/CAMP040	*C. jejuni*	N	N	none	CIP	SXT, AMP, NOR, CTX, CAZ	+	+	+	-	+	-	+	E
40	POUL024/CAMP041	*C. jejuni*	N	N	none	CIP	SXT, AMP, NOR, CTX, CAZ	+	+	+	-	+	-	+	A
41	POUL025/CAMP042	*C. coli*	**S**	**S**	**AM**	TE, CIP	NOR, CTX	+	+	-	+	+	-	+	F
42	POUL026/CAMP043	*C. jejuni*	**S**	**S**	**AM**	TE	none	+	+	+	+	+	-	+	H
43	POUL025/CAMP044	*C. coli*	**S**	**S**	**AM**	TE, CIP	AMP, NOR	+	+	+	+	-	-	+	F
44	POUL026/CAMP045	*C. jejuni*	**S**	**M**	**AM**	TE	none	+	+	+	+	+	-	+	H
45	POUL028/CAMP046	*C. jejuni*	**S**	**S**	**AM**	CIP	AMP, NOR	+	+	+	-	+	-	+	I
46	POUL027/CAMP048	*C. jejuni*	**S**	**M**	**AM**	none	SXT	+	-	-	-	+	-	+	H
47	POUL028/CAMP049	*C. jejuni*	**S**	**S**	**AM**	CIP	SXT, AMP, NOR, CTX	+	+	+	-	+	-	+	I
48	POUL029/CAMP050	*C. jejuni*	N	N	none	none	SXT	+	-	-	-	+	-	+	**J**
49	POUL030/CAMP051	*C. jejuni*	N	N	none	CIP	SXT, NOR	+	+	+	-	+	-	+	**J**
50	POUL028/CAMP052	*C. jejuni*	**S**	**S**	**AM**	CIP	SXT, AMP, NOR	+	+	+	-	+	-	+	I
51	POUL029/CAMP053	*C. jejuni*	N	N	none	none	SXT	+	-	-	-	+	-	+	**J**
52	POUL031/CAMP054	*C. jejuni*	N	N	none	CIP	AMP, NOR, CTX, CAZ	+	+	-	-	+	-	+	I
53	POUL032/CAMP056	*C. coli*	**S**	**S**	**AM**	CIP	SXT, NOR	+	+	+	-	-	-	+	F
54	POUL032/CAMP057	*C. coli*	**S**	**S**	**AM**	CIP	SXT, NOR	+	+	+	-	-	-	+	F
55	POUL032/CAMP058	*C. jejuni*	N	N	none	TE, CIP	SXT, NOR	+	+	+	+	+	-	+	D
56	POUL033/CAMP059	*C. jejuni*	N	N	none	TE, CIP	SXT, AMP, NOR, CAZ	+	+	+	+	+	+	+	**J**
57	POUL033/CAMP062	*C. jejuni*	N	N	none	TE, CIP	SXT, AMP, NOR, CAZ	+	+	+	+	+	+	+	**J**
58	POUL036/CAMP063	*C. jejuni*	N	N	none	TE, CIP	SXT, AMP, NOR, CAZ	+	+	-	+	+	-	+	ND
59	POUL037/CAMP064	*C. jejuni*	N	N	none	TE, CIP	SXT, NOR, CTX	+	+	+	+	+	-	+	**J**
60	POUL037/CAMP065	*C. jejuni*	N	**W**	A	TE, CIP	NOR	+	+	-	+	+	-	+	F
61	POUL038/CAMP066	*C. jejuni*	N	N	none	TE, CIP	SXT, NOR	+	+	-	+	+	-	+	**J**
62	POUL038/CAMP067	*C. jejuni*	N	N	none	TE, CIP	SXT, NOR	+	+	-	+	+	-	+	**J**
63	POUL041/CAMP068	*C. jejuni*	N	N	none	CIP	SXT, NOR	+	+	+	-	+	-	+	F
64	POUL037/CAMP069	*C. jejuni*	N	N	none	TE, CIP	SXT, NOR, CTX	+	+	-	+	+	-	+	H
65	POUL040/CAMP070	*C. jejuni*	**S**	**S**	**AM**	none	SXT, CAZ	+	-	+	-	+	-	+	F
66	POUL041/CAMP071	*C. coli*	N	N	none	none	none	+	-	-	-	+	-	+	F
67	POUL037/CAMP072	*C. coli*	N	N	none	TE, CIP	SXT, NOR, CTX	+	-	-	-	+	-	+	H
68	POUL037/CAMP073	*C. jejuni*	**M**	N	M	TE, CIP	SXT	+	+	-	+	+	-	+	H
69	POUL038/CAMP074	*C. jejuni*	N	N	none	TE, CIP	SXT, NOR	+	+	-	+	+	-	+	**J**
70	POUL038/CAMP075	*C. jejuni*	N	N	none	TE, CIP	SXT, NOR	+	+	-	+	+	-	+	**J**
71	POUL040/CAMP076	*C. jejuni*	**S**	**S**	**AM**	none	SXT	+	-	+	+	+	-	+	F
72	POUL041/CAMP077	*C. jejuni*	N	N	none	CIP	SXT, NOR	+	+	+	+	+	-	+	**J**
73	POUL041/CAMP078	*C. jejuni*	N	N	none	none	none	+	-	+	+	+	-	+	F
74	POUL045/CAMP080	*C. coli*	**S**	**S**	**AM**	TE, CIP	AMP, NOR	+	+	+	+	-	-	+	F
75	POUL045/CAMP081	*C. coli*	**W**	**W**	**AM**	CIP	NOR	+	+	+	-	-	-	+	F
76	POUL044/CAMP082	*C. coli*	**S**	**S**	**AM**	CIP	SXT, NOR	+	+	+	-	-	-	+	F
77	POUL044/CAMP083	*C. coli*	**W**	**M**	**AM**	none	SXT	+	+	+	-	+	-	+	**J**
78	POUL042/CAMP084	*C. jejuni*	N	N	none	CIP	SXT, NOR	+	+	+	-	+	-	+	ND
79	POUL042/CAMP085	*C. jejuni*	N	N	none	CIP	SXT, NOR, CAZ	+	+	+	-	+	-	+	ND
80	POUL043/CAMP086	*C. jejuni*	**W**	**M**	**AM**	TE	SXT	+	-	-	-	+	-	+	F
81	POUL043/CAMP087	*C. jejuni*	**S**	**S**	**AM**	TE, CIP	SXT, AMP, NOR, CTX, CAZ	+	+	-	+	-	-	+	**J**
82	POUL044/CAMP088	*C. jejuni*	N	N	none	TE, CIP	SXT, NOR	+	+	+	+	+	-	+	ND
83	POUL044/CAMP089	*C. coli*	**S**	**S**	**AM**	CIP	NOR	+	+	+	-	-	-	+	ND
84	POUL044/CAMP090	*C. coli*	**W**	**M**	**AM**	none	SXT, CAZ	+	+	+	-	+	-	+	ND
85	POUL045/CAMP091	*C. jejuni*	**W**	**W**	**AM**	E, TE	none	-	-	-	-	+	-	-	**J**
86	POUL045/CAMP092	*C. coli*	**W**	**S**	**AM**	CIP	NOR, CTX	+	+	+	-	-	-	+	F
87	POUL046/CAMP093	*C. jejuni*	N	N	none	TE, CIP	SXT, AMP, NOR, CAZ	ND	ND	ND	ND	ND	ND	ND	ND
88	POUL046/CAMP094	*C. jejuni*	N	N	none	TE, CIP	SXT, AMP, NOR, CTX	+	+	+	+	+	-	+	I
89	POUL047/CAMP095	*C. coli*	N	N	none	TE, CIP	GEN, NOR, CAZ	ND	ND	ND	ND	ND	ND	ND	ND
90	POUL048/CAMP096	*C. coli*	**S**	**S**	**AM**	TE, CIP	NOR	+	+	+	+	-	-	+	F
91	POUL047/CAMP097	*C. coli*	N	N	none	**E, TE, CIP**	AMP, NOR, CTX	+	+	+	-	-	-	+	F
92	POUL048/CAMP098	*C. coli*	**S**	**M**	**AM**	none	none	+	-	-	-	-	-	+	B
93	POUL048/CAMP099	*C. coli*	N	N	none	TE, CIP	NOR	+	+	-	+	+	-	+	**J**
94	POUL049/CAMP100	*C. jejuni*	**S**	**S**	**AM**	none	SXT	+	-	+	-	+	-	+	**J**
95	POUL048/CAMP102	*C. coli*	N	N	none	TE, CIP	NOR	+	+	-	+	+	-	+	F
96	POUL048/CAMP103	*C. jejuni*	**S**	**S**	**AM**	none	SXT	+	-	+	-	+	-	+	**J**
97	POUL048/CAMP104	*C. jejuni*	N	N	none	CIP	SXT, AMP, NOR	+	+	+	-	+	-	+	ND
98	POUL048/CAMP105	*C. jejuni*	N	N	none	TE, CIP	SXT, AMP, NOR, CTX	+	+	-	+	+	-	+	H
99	POUL048/CAMP106	*C. jejuni*	**S**	**S**	**AM**	CIP	SXT, AMP, NOR, CAZ	+	+	+	-	+	-	+	F
100	POUL053/CAMP107	*C. coli*	**S**	**S**	**AM**	TE, CIP	NOR	+	+	+	+	-	-	+	D
101	POUL055/CAMP108	*C. jejuni*	**W**	N	M	TE, CIP	SXT, AMP, NOR	+	+	-	+	+	-	+	**J**
102	POUL056/CAMP109	*C. jejuni*	**W**	N	M	CIP	SXT, NOR, CTX	+	+	+	-	+	-	+	**J**
103	POUL053/CAMP110	*C. coli*	**S**	**M**	**AM**	CIP	NOR	+	+	+	+	-	-	+	I
104	POUL055/CAMP111	*C. jejuni*	N	N	none	TE, CIP	SXT, AMP, NOR	+	+	-	+	+	-	+	H
105	POUL056/CAMP112	*C. jejuni*	**W**	N	M	CIP	SXT, NOR	+	+	+	-	+	-	+	**J**
106	POUL056/CAMP113	*C. jejuni*	N	N	none	CIP	SXT, NOR	+	+	+	-	+	-	+	H
107	POUL055/CAMP114	*C. jejuni*	N	**M**	A	TE, CIP	AMP, NOR	+	+	-	+	+	-	+	**J**
108	POUL053/CAMP117	*C. jejuni*	N	N	none	CIP	NOR, CTX	+	+	+	-	+	-	+	ND
109	POUL057/CAMP119	*C. coli*	N	N	none	CIP	NOR, CAZ	+	-	+	-	+	-	+	ND
110	POUL057/CAMP121	*C. coli*	**M**	N	M	TE, CIP	NOR	+	+	+	+	+	-	+	F
111	POUL058/CAMP122	*C. coli*	N	N	none	TE, CIP	AMP, NOR	+	+	-	+	+	-	+	ND
112	POUL060/CAMP123	*C. jejuni*	**S**	**S**	**AM**	none	SXT	+	-	+	-	+	-	+	**J**
113	POUL060/CAMP124	*C. jejuni*	**S**	**S**	**AM**	none	SXT, CTX, CAZ	+	-	+	-	+	-	+	H
114	POUL061/CAMP125	*C. jejuni*	**S**	**S**	**AM**	none	SXT, CTX, CAZ	+	-	+	-	+	-	+	**J**
115	POUL062/CAMP126	*C. jejuni*	N	N	none	TE, CIP	SXT, NOR	+	+	+	+	+	+	+	**J**
116	POUL063/CAMP127	*C. coli*	N	N	none	CIP	NOR, CTX	+	+	+	-	+	-	+	F
117	POUL062/CAMP129	*C. jejuni*	N	N	none	TE, CIP	SXT, NOR	+	+	+	+	+	+	+	D
118	POUL064/CAMP130	*C. jejuni*	**W**	**M**	**AM**	TE, CIP	none	+	+	+	+	+	-	+	**J**
119	POUL061/CAMP131	*C. jejuni*	**S**	**S**	**AM**	CIP	CTX, CAZ	+	-	+	-	+	-	+	F
120	POUL062/CAMP132	*C. jejuni*	N	N	none	E	SXT	+	+	+	+	+	+	+	H

A: biofilm formation under aerobic CO_2-enriched conditions; **M**: biofilm formation under microaerophilic conditions; **AM**: biofilm formation under both conditions; **ND**: not determined due to insufficient DNA quantity

*Antibiotics listed under Antibiotic resistance profile correspond to isolates classified as resistant according to EUCAST 2026 breakpoints for *Campylobacter* spp.

**Additional non-inhibition profile includes antimicrobials with absence of inhibition zone for which no *Campylobacter*-specific EUCAST breakpoints were available and interpretation was based on descriptive reporting rather than standardized *Campylobacter* breakpoints.

ATCC 33291 was included as reference *C. jejuni* strain.
