# Supplementary material for: Integrated Phenotypic and Genotypic Analysis Reveals Distinct Patterns of Biofilm Formation and Antimicrobial Resistance in Poultry-Derived Campylobacter jejuni and Campylobacter coli
Source: Foods. 2026 Oct 25;15(21):3815. doi: 10.3390/foods15213815 (PMC13763043; doi:10.3390/foods15213815)
Supplement: Supplementary file 1 [file foods-15-03815-s001.zip › Supplementary Table S12.pdf]

Table S12. Association between biofilm formation and antimicrobial response phenotypes among the 120 *Campylobacter* isolates. Biofilm formation (yes/no; A_{590} > Ac) in MH-CJ medium was analysed separately for each atmospheric condition and compared with the number of EUCAST-resistant classes (E, TE, CIP) and with antimicrobial response complexity, overall and within each species.

A. Antimicrobial response phenotypes in biofilm formers and non-formers

Group	Phenotype	Microaerophilic			Aerobic CO_2-enriched		
		Formers, n/N (%)	Non-formers, n/N (%)	*p*	Formers, n/N (%)	Non-formers, n/N (%)	*p*
All isolates	Resistant to ≥2 EUCAST classes (E, TE, CIP)	18/57 (31.6)	42/63 (66.7)	<0.001	17/55 (30.9)	43/65 (66.2)	<0.001
	Susceptible to E, TE and CIP	11/57 (19.3)	4/63 (6.3)	0.051	11/55 (20.0)	4/65 (6.2)	0.028
	High complexity (≥3 affected classes)	17/57 (29.8)	42/63 (66.7)	<0.001	15/55 (27.3)	44/65 (67.7)	<0.001
C. jejuni	Resistant to ≥2 EUCAST classes (E, TE, CIP)	8/26 (30.8)	35/52 (67.3)	0.003	8/24 (33.3)	35/54 (64.8)	0.014
	Susceptible to E, TE and CIP	8/26 (30.8)	3/52 (5.8)	0.005	8/24 (33.3)	3/54 (5.6)	0.003
	High complexity (≥3 affected classes)	10/26 (38.5)	37/52 (71.2)	0.007	8/24 (33.3)	39/54 (72.2)	0.002
C. coli	Resistant to ≥2 EUCAST classes (E, TE, CIP)	10/31 (32.3)	7/11 (63.6)	0.086	9/31 (29.0)	8/11 (72.7)	0.029
	Susceptible to E, TE and CIP	3/31 (9.7)	1/11 (9.1)	1.000	3/31 (9.7)	1/11 (9.1)	1.000
	High complexity (≥3 affected classes)	7/31 (22.6)	5/11 (45.5)	0.243	7/31 (22.6)	5/11 (45.5)	0.243

B. Species-adjusted association between biofilm formation and antimicrobial response phenotypes

Outcome	Method	Microaerophilic		Aerobic CO_2-enriched	
		OR (95% CI)	*p*	OR (95% CI)	*p*
Resistant to ≥2 EUCAST classes (E, TE, CIP)	Crude (all isolates; Fisher's exact test; sample OR)	0.23	<0.001	0.23	<0.001
	Species-adjusted (Mantel-Haenszel)	0.23 (0.10–0.53)	<0.001	0.23 (0.10–0.53)	<0.001
	Species-adjusted (Firth logistic regression)	0.24 (0.11–0.55)	<0.001	0.24 (0.10–0.55)	<0.001
Susceptible to E, TE and CIP	Crude (all isolates; Fisher's exact test; sample OR)	3.53	0.051	3.81	0.028
	Species-adjusted (Mantel-Haenszel)	4.22 (1.23–14.55)	0.021	4.64 (1.33–16.14)	0.012

	Species-adjusted (Firth logistic regression)	4.65 (1.36–15.97)	0.015	5.33 (1.53–18.52)	0.009
High complexity (≥3 affected classes)	Crude (all isolates; Fisher's exact test; sample OR)	0.21	<0.001	0.18	<0.001
	Species-adjusted (Mantel-Haenszel)	0.28 (0.12–0.63)	0.004	0.23 (0.10–0.54)	0.001
	Species-adjusted (Firth logistic regression)	0.29 (0.13–0.65)	0.003	0.24 (0.11–0.56)	<0.001

C. Number of EUCAST-resistant classes (0–3) and biofilm biomass (A_{590})

**Group**	**Comparison**	**Microaerophilic**		**Aerobic CO_2-enriched**	
		**Result**	***p***	**Result**	***p***
All isolates	Median (IQR) classes: formers vs non-formers; Mann-Whitney U test	1 (1–2) vs 2 (1–2)	<0.001	1 (1–2) vs 2 (1–2)	<0.001
C. jejuni	Median (IQR) classes: formers vs non-formers; Mann-Whitney U test	1 (0–2) vs 2 (1–2)	<0.001	1 (0–2) vs 2 (1–2)	0.002
C. coli	Median (IQR) classes: formers vs non-formers; Mann-Whitney U test	1 (1–2) vs 2 (1–2)	0.091	1 (1–2) vs 2 (2–2)	0.023
C. jejuni	Spearman rank correlation between A590 and number of resistant classes (rho)	-0.37	<0.001	-0.36	0.001
C. coli	Spearman rank correlation between A590 and number of resistant classes (rho)	-0.02	0.902	-0.05	0.757

OR, odds ratio; CI, confidence interval; IQR, interquartile range. Biofilm formers: weak, moderate or strong biofilm formers (A_{590} > Ac) in MH-CJ medium (Ac = 0.78 microaerophilic; 0.69 aerobic CO_2-enriched; Table S3). n/N: number of isolates with the phenotype / number of formers or non-formers in the group. Percentages are within the formers or non-formers.

Resistance was defined with EUCAST v. 2026 breakpoints for E, TE and CIP only (three classes). Complexity was defined as in Section 2.5 (number of affected antimicrobial classes, counting EUCAST resistance for E, TE and CIP and complete non-inhibition for GEN, SXT, AMP, NOR, MEM, CTX and CAZ; CIP and NOR, and CTX and CAZ, each counted as one class); high complexity ≥ 3 classes. Complete non-inhibition is not resistance.

ORs < 1 indicate lower odds of the phenotype in biofilm formers; for susceptibility, ORs > 1 indicate higher odds of being susceptible in formers. The Mantel-Haenszel OR combines the species-specific 2×2 tables (95% CI: Robins-Breslow-Greenland variance; *p*: continuity-corrected Mantel-Haenszel χ^2 test). Firth penalized-likelihood logistic regression included biofilm formation and species (*C. coli vs C. jejuni*) as predictors; 95% CIs and *p*-values are Wald-type.

The association between biofilm formation and resistance to ≥ 2 classes remained significant after Bonferroni, Holm and Benjamini–Hochberg correction across all 32 tests reported in the study.

All analyses are exploratory and were not adjusted for multiple comparisons. Isolates from the same sample were treated as independent. Analyses include all 120 isolates (no genotypic data were required).
