# Supplementary material for: Integrated Phenotypic and Genotypic Analysis Reveals Distinct Patterns of Biofilm Formation and Antimicrobial Resistance in Poultry-Derived Campylobacter jejuni and Campylobacter coli
Source: Foods. 2026 Oct 25;15(21):3815. doi: 10.3390/foods15213815 (PMC13763043; doi:10.3390/foods15213815)
Supplement: Supplementary file 1 [file foods-15-03815-s001.zip › Supplementary Table S2.pdf]

Table S2. Primers and PCR targets used for the detection of AMR and virulence-associated genes in *C. jejuni* and *C. coli* isolates.

s/n	Target	Primer name	Sequence (5'→3')	Length (nt)	GC(%)	Tm (°C)	Amplicon size (bp)	Reference
1	*gyrA* Thr-86-to-Ile mutation (*C. jejuni*)	CampyMAMAgyrA1	TTTTTAGCAAAGATTCTGAT	20	25	47.1	265	[45]
2	*gyrA* Thr-86-to-Ile mutation (*C. jejuni*)	CampyMAMAgyrA5	CAAAGCATCATAAACTGCAA	20	35	51.1		
3	*gyrA* internal control (*C. jejuni*)	GZgyrA4	CAGTATAACGCATCGCAGCG	20	55	59.3	368	
4	*gyrA* Thr-86-to-Ile mutation (*C. coli*)	GZgyrACcoli3F	TATGAGCGTTATTATCGGTC	20	40	53.2	192	[46]
5	*gyrA* Thr-86-to-Ile mutation (*C. coli*)	CampyMAMAgyrA8	TAAGGCATCGTAAACAGCCA	20	45	55.3		
6	*gyrA* internal control (*C. coli*)	GZgyrACcoli4R	GTCCATCTACAAGCTCGTTA	20	45	55.3	505	
7	*bla*$_{OXA-61}$	blaOXA-61_F	AGAGTATAATACAAGCG	17	35.3	45.5	372	[47]
8	*bla*$_{OXA-61}$	blaOXA-61_R	TAGTGAGTTGTCAAGCC	17	47.1	50.4		
9	*tet(O)*	tet(O)_F	GGCGTTTTGTTTATGTGCG	19	47.4	54.5	559	[48]
10	*tet(O)*	tet(O)_R	ATGGACAACCCGACAGAAGC	20	55	59.3		
11	*cmeB*	cmeB_F	AGGCGGTTTTGAAATGTATGTT	22	36.4	54.7	444	[49]
12	*cmeB*	cmeB_R	TGTGCCGCTGGGAAAAG	17	58.8	55.2		
13	*aphA-3-1*	aphA-3-1_F	TGCGTAAAAGATACGGAAG	19	42.1	52.4	701	[47]
14	*aphA-3-1*	aphA-3-1_R	CAATCAGGCTTGATCCCC	18	55.6	56		
15	*cdtB* (*C. jejuni*)	Cjeju_cdtB_F	ATCTTTTAACCTTGCTTTTGC	21	33.3	52	714	[50]
16	*cdtB* (*C. jejuni*)	Cjeju_cdtB_R	GCAAGCATTAAAATCGCAGC	20	45	55.3		
17	*cdtB* (*C. coli*)	Ccoli_cdtB_F	TTTAATGTATTATTTGCCGC	20	30	49.1	413	[50]
18	*cdtB* (*C. coli*)	Ccoli_cdtB_R	TCATTGCCTATGCGTATG	18	44.4	51.4		

F: forward primer; R: reverse primer. Tm: melting temperature; nt: nucleotide; bp: base pair.
