# Supplementary material for: Integrated Phenotypic and Genotypic Analysis Reveals Distinct Patterns of Biofilm Formation and Antimicrobial Resistance in Poultry-Derived Campylobacter jejuni and Campylobacter coli
Source: Foods. 2026 Oct 25;15(21):3815. doi: 10.3390/foods15213815 (PMC13763043; doi:10.3390/foods15213815)
Supplement: Supplementary file 1 [file foods-15-03815-s001.zip › Supplementary Table S3.pdf]

Table S3. Biofilm biomass (A_{590}) for the 120 *Campylobacter* isolates and classification of each isolate as a non-biofilm former (Z), weak (W), moderate (M), or strong biofilm former (S). Each isolate was incubated in MH-CJ or MH-HB broth at 42 °C for 48 h under either microaerophilic or aerobic CO_2-enriched conditions to allow biofilm formation. Biofilm absorbance data are presented as mean ± standard deviation. Cut-off absorbance (Ac) values and the corresponding thresholds used for biofilm classification under each growth condition are also provided.

			**MH-CJ**				**MH-HB**			
			**Microaerophilic conditions**		**Aerobic CO_2-enriched conditions**		**Microaerophilic conditions**		**Aerobic CO_2-enriched conditions**	
**s/n**	**Isolate code**	**Species**	**A_{590}**	**Characterization**	**A_{590}**	**Characterization**	**A_{590}**	**Characterization**	**A_{590}**	**Characterization**
	ATCC 33291	*C. jejuni*	0.62 ± 0.11	Z	0.46 ± 0.10	Z	0.43 ± 0.03	Z	0.53 ± 0.21	Z
1	CAMP001	*C. jejuni*	0.35 ± 0.15	Z	0.33 ± 0.12	Z	0.25 ± 0.03	Z	0.53 ± 0.15	Z
2	CAMP003	*C. jejuni*	0.50 ± 0.12	Z	0.66 ± 0.07	Z	0.23 ± 0.06	Z	0.30 ± 0.05	Z
3	CAMP004	*C. coli*	1.44 ± 0.22	W	1.87 ± 0.36	M	0.36 ± 0.13	Z	0.40 ± 0.07	Z
4	CAMP005	*C. coli*	3.83 ± 0.35	S	3.85 ± 0.29	S	0.66 ± 0.12	Z	0.48 ± 0.17	Z
5	CAMP006	*C. jejuni*	0.28 ± 0.10	Z	0.44 ± 0.12	Z	0.31 ± 0.08	Z	0.31 ± 0.02	Z
6	CAMP007	*C. jejuni*	0.35 ± 0.13	Z	0.41 ± 0.06	Z	0.48 ± 0.15	Z	0.72 ± 0.13	Z
7	CAMP008	*C. jejuni*	0.35 ± 0.11	Z	0.40 ± 0.06	Z	0.55 ± 0.14	Z	0.72 ± 0.05	Z
8	CAMP009	*C. jejuni*	0.50 ± 0.15	Z	0.46 ± 0.08	Z	0.25 ± 0.05	Z	0.34 ± 0.04	Z
9	CAMP010	*C. coli*	0.66 ± 0.11	Z	3.31 ± 0.59	S	0.31 ± 0.11	Z	0.34 ± 0.02	Z
10	CAMP011	*C. jejuni*	0.32 ± 0.12	Z	0.41 ± 0.10	Z	0.47 ± 0.17	Z	0.92 ± 0.13	Z
11	CAMP012	*C. jejuni*	0.36 ± 0.14	Z	0.42 ± 0.03	Z	0.37 ± 0.03	Z	0.87 ± 0.09	Z
12	CAMP013	*C. jejuni*	0.35 ± 0.10	Z	0.41 ± 0.05	Z	0.51 ± 0.20	Z	0.90 ± 0.10	Z
13	CAMP014	*C. coli*	2.73 ± 0.66	M	2.12 ± 0.38	M	0.45 ± 0.02	Z	0.55 ± 0.04	Z
14	CAMP015	*C. coli*	1.59 ± 0.12	M	0.95 ± 0.26	W	0.44 ± 0.12	Z	0.57 ± 0.20	Z
15	CAMP016	*C. coli*	1.48 ± 0.20	W	1.71 ± 0.36	M	0.43 ± 0.06	Z	0.84 ± 0.07	Z
16	CAMP017	*C. coli*	1.78 ± 0.31	M	0.95 ± 0.10	W	0.56 ± 0.20	Z	0.72 ± 0.11	Z
17	CAMP018	*C. jejuni*	4.00 ± 0.00	S	3.96 ± 0.05	S	0.51 ± 0.21	Z	0.30 ± 0.03	Z
18	CAMP019	*C. jejuni*	0.20 ± 0.03	Z	0.28 ± 0.04	Z	0.18 ± 0.01	Z	0.31 ± 0.02	Z
19	CAMP020	*C. jejuni*	0.20 ± 0.03	Z	0.25 ± 0.03	Z	0.18 ± 0.02	Z	0.30 ± 0.06	Z

20	CAMP021	*C. coli*	1.44 ± 0.54	W	1.13 ± 0.34	W	0.34 ± 0.04	Z	0.63 ± 0.11	Z
21	CAMP022	*C. jejuni*	0.27 ± 0.06	Z	3.33 ± 0.49	S	0.18 ± 0.01	Z	0.28 ± 0.02	Z
22	CAMP023	*C. jejuni*	3.91 ± 0.13	S	0.48 ± 0.09	Z	0.21 ± 0.01	Z	0.30 ± 0.02	Z
23	CAMP024	*C. jejuni*	1.71 ± 0.16	M	2.38 ± 0.67	M	0.26 ± 0.06	Z	0.39 ± 0.06	Z
24	CAMP025	*C. coli*	1.70 ± 0.29	M	2.75 ± 0.50	M	0.23 ± 0.03	Z	0.36 ± 0.05	Z
25	CAMP026	*C. coli*	2.08 ± 0.22	M	2.69 ± 0.61	M	0.23 ± 0.03	Z	0.41 ± 0.04	Z
26	CAMP027	*C. jejuni*	0.31 ± 0.11	Z	0.36 ± 0.07	Z	0.19 ± 0.01	Z	0.30 ± 0.05	Z
27	CAMP028	*C. jejuni*	0.18 ± 0.02	Z	0.29 ± 0.03	Z	0.18 ± 0.01	Z	0.30 ± 0.05	Z
28	CAMP029	*C. jejuni*	0.20 ± 0.01	Z	0.34 ± 0.03	Z	0.19 ± 0.01	Z	0.32 ± 0.05	Z
29	CAMP030	*C. coli*	0.37 ± 0.13	Z	0.38 ± 0.03	Z	0.26 ± 0.03	Z	0.43 ± 0.11	Z
30	CAMP031	*C. coli*	1.35 ± 0.27	W	1.97 ± 0.29	M	0.21 ± 0.01	Z	0.39 ± 0.10	Z
31	CAMP032	*C. coli*	4.00 ± 0.00	S	4.00 ± 0.00	S	0.24 ± 0.02	Z	0.44 ± 0.13	Z
32	CAMP033	*C. coli*	0.82 ± 0.10	W	2.31 ± 0.37	M	0.27 ± 0.03	Z	0.46 ± 0.05	Z
33	CAMP034	*C. coli*	4.00 ± 0.00	S	4.00 ± 0.00	S	0.36 ± 0.11	Z	0.44 ± 0.06	Z
34	CAMP035	*C. jejuni*	0.30 ± 0.03	Z	0.50 ± 0.12	Z	0.20 ± 0.01	Z	0.29 ± 0.03	Z
35	CAMP036	*C. jejuni*	0.26 ± 0.01	Z	0.49 ± 0.08	Z	0.19 ± 0.01	Z	0.28 ± 0.01	Z
36	CAMP037	*C. jejuni*	0.28 ± 0.05	Z	0.32 ± 0.01	Z	0.21 ± 0.02	Z	0.39 ± 0.06	Z
37	CAMP038	*C. coli*	3.87 ± 0.15	S	4.00 ± 0.00	S	0.42 ± 0.08	Z	0.75 ± 0.12	Z
38	CAMP039	*C. coli*	1.60 ± 0.53	M	2.88 ± 0.43	S	0.37 ± 0.07	Z	0.52 ± 0.04	Z
39	CAMP040	*C. jejuni*	0.37 ± 0.06	Z	0.55 ± 0.20	Z	0.23 ± 0.04	Z	0.41 ± 0.14	Z
40	CAMP041	*C. jejuni*	0.27 ± 0.04	Z	0.44 ± 0.08	Z	0.31 ± 0.02	Z	0.78 ± 0.21	Z
41	CAMP042	*C. coli*	3.75 ± 0.21	S	3.77 ± 0.29	S	0.29 ± 0.06	Z	0.46 ± 0.09	Z
42	CAMP043	*C. jejuni*	4.00 ± 0.00	S	3.26 ± 0.27	S	0.40 ± 0.27	Z	0.63 ± 0.05	Z
43	CAMP044	*C. coli*	4.00 ± 0.00	S	4.00 ± 0.00	S	0.26 ± 0.06	Z	0.41 ± 0.13	Z
44	CAMP045	*C. jejuni*	3.91 ± 0.13	S	1.81 ± 0.43	M	0.33 ± 0.15	Z	0.57 ± 0.16	Z
45	CAMP046	*C. jejuni*	3.70 ± 0.30	S	3.87 ± 0.15	S	0.25 ± 0.06	Z	0.39 ± 0.08	Z
46	CAMP048	*C. jejuni*	3.82 ± 0.29	S	2.65 ± 0.60	M	0.26 ± 0.05	Z	0.39 ± 0.09	Z
47	CAMP049	*C. jejuni*	3.77 ± 0.28	S	3.94 ± 0.16	S	0.29 ± 0.08	Z	0.41 ± 0.07	Z
48	CAMP050	*C. jejuni*	0.25 ± 0.04	Z	0.32 ± 0.05	Z	0.21 ± 0.02	Z	0.33 ± 0.04	Z
49	CAMP051	*C. jejuni*	0.27 ± 0.10	Z	0.33 ± 0.05	Z	0.22 ± 0.03	Z	0.33 ± 0.03	Z
50	CAMP052	*C. jejuni*	4.00 ± 0.00	S	4.00 ± 0.00	S	0.29 ± 0.12	Z	0.36 ± 0.06	Z

51	CAMP053	*C. jejuni*	0.26 ± 0.01	Z	0.34 ± 0.03	Z	0.20 ± 0.02	Z	0.36 ± 0.05	Z
52	CAMP054	*C. jejuni*	0.24 ± 0.05	Z	0.41 ± 0.12	Z	0.21 ± 0.03	Z	0.28 ± 0.02	Z
53	CAMP056	*C. coli*	4.00 ± 0.00	S	4.00 ± 0.00	S	0.40 ± 0.06	Z	0.42 ± 0.12	Z
54	CAMP057	*C. coli*	3.86 ± 0.10	S	3.89 ± 0.25	S	0.27 ± 0.05	Z	0.35 ± 0.06	Z
55	CAMP058	*C. jejuni*	0.33 ± 0.06	Z	0.42 ± 0.10	Z	0.25 ± 0.04	Z	0.34 ± 0.11	Z
56	CAMP059	*C. jejuni*	0.28 ± 0.10	Z	0.36 ± 0.10	Z	0.25 ± 0.05	Z	0.31 ± 0.07	Z
57	CAMP062	*C. jejuni*	0.18 ± 0.01	Z	0.24 ± 0.02	Z	0.19 ± 0.01	Z	0.25 ± 0.02	Z
58	CAMP063	*C. jejuni*	0.18 ± 0.00	Z	0.25 ± 0.05	Z	0.23 ± 0.05	Z	0.25 ± 0.03	Z
59	CAMP064	*C. jejuni*	0.19 ± 0.00	Z	0.26 ± 0.05	Z	0.21 ± 0.01	Z	0.29 ± 0.05	Z
60	CAMP065	*C. jejuni*	0.74 ± 0.25	Z	1.31 ± 0.23	W	0.34 ± 0.07	Z	0.39 ± 0.10	Z
61	CAMP066	*C. jejuni*	0.22 ± 0.02	Z	0.34 ± 0.18	Z	0.22 ± 0.03	Z	0.33 ± 0.05	Z
62	CAMP067	*C. jejuni*	0.26 ± 0.04	Z	0.32 ± 0.06	Z	0.28 ± 0.01	Z	0.42 ± 0.04	Z
63	CAMP068	*C. jejuni*	0.30 ± 0.08	Z	0.31 ± 0.04	Z	0.25 ± 0.01	Z	0.39 ± 0.02	Z
64	CAMP069	*C. jejuni*	0.63 ± 0.20	Z	0.52 ± 0.14	Z	0.25 ± 0.03	Z	0.58 ± 0.14	Z
65	CAMP070	*C. jejuni*	3.96 ± 0.07	S	3.69 ± 0.32	S	0.49 ± 0.18	Z	0.50 ± 0.10	Z
66	CAMP071	*C. coli*	0.60 ± 0.11	Z	0.52 ± 0.12	Z	0.62 ± 0.17	Z	0.68 ± 0.15	Z
67	CAMP072	*C. coli*	0.58 ± 0.06	Z	0.47 ± 0.09	Z	0.70 ± 0.19	Z	0.61 ± 0.08	Z
68	CAMP073	*C. jejuni*	1.67 ± 0.35	M	0.62 ± 0.12	Z	0.80 ± 0.04	Z	0.48 ± 0.11	Z
69	CAMP074	*C. jejuni*	0.25 ± 0.02	Z	0.23 ± 0.02	Z	0.29 ± 0.03	Z	0.29 ± 0.03	Z
70	CAMP075	*C. jejuni*	0.27 ± 0.03	Z	0.26 ± 0.02	Z	0.26 ± 0.01	Z	0.29 ± 0.04	Z
71	CAMP076	*C. jejuni*	3.98 ± 0.03	S	3.56 ± 0.23	S	0.38 ± 0.09	Z	0.35 ± 0.04	Z
72	CAMP077	*C. jejuni*	0.23 ± 0.02	Z	0.27 ± 0.01	Z	0.29 ± 0.08	Z	0.44 ± 0.08	Z
73	CAMP078	*C. jejuni*	0.24 ± 0.02	Z	0.27 ± 0.02	Z	0.23 ± 0.02	Z	0.36 ± 0.04	Z
74	CAMP080	*C. coli*	3.90 ± 0.21	S	4.00 ± 0.00	S	0.56 ± 0.21	Z	0.49 ± 0.18	Z
75	CAMP081	*C. coli*	0.96 ± 0.23	W	0.77 ± 0.19	W	0.32 ± 0.10	Z	0.43 ± 0.09	Z
76	CAMP082	*C. coli*	3.92 ± 0.20	S	3.89 ± 0.23	S	0.49 ± 0.24	Z	0.66 ± 0.14	Z
77	CAMP083	*C. coli*	1.31 ± 0.36	W	1.87 ± 0.23	M	0.34 ± 0.10	Z	0.50 ± 0.13	Z
78	CAMP084	*C. jejuni*	0.44 ± 0.07	Z	0.37 ± 0.13	Z	0.64 ± 0.11	Z	0.48 ± 0.15	Z
79	CAMP085	*C. jejuni*	0.37 ± 0.03	Z	0.46 ± 0.07	Z	0.64 ± 0.06	Z	0.77 ± 0.09	Z
80	CAMP086	*C. jejuni*	1.35 ± 0.23	W	1.56 ± 0.46	M	0.32 ± 0.06	Z	0.38 ± 0.05	Z
81	CAMP087	*C. jejuni*	3.21 ± 0.69	S	3.10 ± 0.50	S	0.35 ± 0.14	Z	0.34 ± 0.03	Z

82	CAMP088	*C. jejuni*	0.51 ± 0.08	Z	0.45 ± 0.05	Z	0.64 ± 0.15	Z	0.66 ± 0.04	Z
83	CAMP089	*C. coli*	4.00 ± 0.00	S	4.00 ± 0.00	S	0.58 ± 0.33	Z	0.44 ± 0.16	Z
84	CAMP090	*C. coli*	1.48 ± 0.10	W	2.34 ± 0.44	M	0.45 ± 0.14	Z	0.59 ± 0.13	Z
85	CAMP091	*C. jejuni*	1.12 ± 0.31	W	0.92 ± 0.20	W	0.64 ± 0.13	Z	0.91 ± 0.19	Z
86	CAMP092	*C. coli*	1.19 ± 0.08	W	3.57 ± 0.44	S	0.55 ± 0.17	Z	0.64 ± 0.16	Z
87	CAMP093	*C. jejuni*	0.66 ± 0.16	Z	0.55 ± 0.26	Z	0.55 ± 0.18	Z	0.39 ± 0.07	Z
88	CAMP094	*C. jejuni*	0.48 ± 0.13	Z	0.45 ± 0.14	Z	0.53 ± 0.06	Z	0.35 ± 0.02	Z
89	CAMP095	*C. coli*	0.50 ± 0.16	Z	0.41 ± 0.09	Z	0.57 ± 0.15	Z	0.54 ± 0.09	Z
90	CAMP096	*C. coli*	4.00 ± 0.00	S	4.00 ± 0.00	S	0.22 ± 0.03	Z	0.48 ± 0.16	Z
91	CAMP097	*C. coli*	0.42 ± 0.03	Z	0.45 ± 0.07	Z	0.44 ± 0.16	Z	0.34 ± 0.04	Z
92	CAMP098	*C. coli*	3.97 ± 0.06	S	1.82 ± 0.35	M	0.48 ± 0.18	Z	0.51 ± 0.03	Z
93	CAMP099	*C. coli*	0.67 ± 0.11	Z	0.46 ± 0.12	Z	0.67 ± 0.04	Z	0.58 ± 0.08	Z
94	CAMP100	*C. jejuni*	4.00 ± 0.00	S	4.00 ± 0.00	S	0.82 ± 0.23	Z	0.59 ± 0.16	Z
95	CAMP102	*C. coli*	0.42 ± 0.05	Z	0.36 ± 0.02	Z	0.26 ± 0.04	Z	0.44 ± 0.11	Z
96	CAMP103	*C. jejuni*	3.75 ± 0.24	S	4.00 ± 0.00	S	0.73 ± 0.23	Z	0.66 ± 0.07	Z
97	CAMP104	*C. jejuni*	0.33 ± 0.16	Z	0.29 ± 0.04	Z	0.28 ± 0.03	Z	0.36 ± 0.03	Z
98	CAMP105	*C. jejuni*	0.29 ± 0.05	Z	0.26 ± 0.02	Z	0.30 ± 0.04	Z	0.38 ± 0.04	Z
99	CAMP106	*C. jejuni*	3.36 ± 0.52	S	3.46 ± 0.26	S	0.35 ± 0.07	Z	0.41 ± 0.06	Z
100	CAMP107	*C. coli*	3.20 ± 0.74	S	3.67 ± 0.24	S	0.27 ± 0.02	Z	0.42 ± 0.12	Z
101	CAMP108	*C. jejuni*	0.86 ± 0.27	W	0.67 ± 0.22	Z	0.36 ± 0.04	Z	0.41 ± 0.07	Z
102	CAMP109	*C. jejuni*	1.08 ± 0.18	W	0.60 ± 0.17	Z	0.57 ± 0.29	Z	0.66 ± 0.19	Z
103	CAMP110	*C. coli*	3.50 ± 0.43	S	2.66 ± 0.32	M	0.42 ± 0.09	Z	0.38 ± 0.03	Z
104	CAMP111	*C. jejuni*	0.36 ± 0.05	Z	0.44 ± 0.11	Z	0.34 ± 0.03	Z	0.47 ± 0.05	Z
105	CAMP112	*C. jejuni*	1.43 ± 0.16	W	0.65 ± 0.12	Z	0.47 ± 0.09	Z	0.48 ± 0.10	Z
106	CAMP113	*C. jejuni*	0.66 ± 0.25	Z	0.37 ± 0.05	Z	0.33 ± 0.06	Z	0.53 ± 0.10	Z
107	CAMP114	*C. jejuni*	0.46 ± 0.13	Z	1.64 ± 0.36	M	0.36 ± 0.06	Z	0.44 ± 0.07	Z
108	CAMP117	*C. jejuni*	0.25 ± 0.02	Z	0.28 ± 0.02	Z	0.29 ± 0.04	Z	0.39 ± 0.05	Z
109	CAMP119	*C. coli*	0.23 ± 0.05	Z	0.49 ± 0.17	Z	0.20 ± 0.01	Z	0.38 ± 0.09	Z
110	CAMP121	*C. coli*	2.76 ± 0.59	M	0.23 ± 0.02	Z	0.49 ± 0.31	Z	0.30 ± 0.06	Z
111	CAMP122	*C. coli*	0.51 ± 0.05	Z	0.54 ± 0.09	Z	0.19 ± 0.02	Z	0.34 ± 0.06	Z
112	CAMP123	*C. jejuni*	3.90 ± 0.22	S	3.99 ± 0.03	S	0.52 ± 0.23	Z	0.74 ± 0.24	Z

113	CAMP124	*C. jejuni*	3.96 ± 0.10	S	4.00 ± 0.00	S	0.63 ± 0.20	Z	0.60 ± 0.20	Z
114	CAMP125	*C. jejuni*	4.00 ± 0.00	S	3.77 ± 0.22	S	0.33 ± 0.04	Z	0.59 ± 0.10	Z
115	CAMP126	*C. jejuni*	0.67 ± 0.09	Z	0.54 ± 0.13	Z	0.42 ± 0.03	Z	0.68 ± 0.12	Z
116	CAMP127	*C. coli*	0.32 ± 0.06	Z	0.44 ± 0.06	Z	0.21 ± 0.04	Z	0.34 ± 0.09	Z
117	CAMP129	*C. jejuni*	0.58 ± 0.18	Z	0.43 ± 0.03	Z	0.36 ± 0.05	Z	0.56 ± 0.08	Z
118	CAMP130	*C. jejuni*	1.11 ± 0.17	W	1.52 ± 0.30	M	0.50 ± 0.22	Z	0.56 ± 0.09	Z
119	CAMP131	*C. jejuni*	3.72 ± 0.37	S	3.85 ± 0.31	S	0.57 ± 0.29	Z	0.50 ± 0.11	Z
120	CAMP132	*C. jejuni*	0.63 ± 0.16	Z	0.57 ± 0.10	Z	0.75 ± 0.37	Z	0.65 ± 0.15	Z

Ac = mean A_{590} of the negative control + 3 SD, determined separately for each medium and atmosphere. Each value is the mean ± SD of at least two independent experiments with two technical replicates each.

A_{590} = 4.00 denotes the upper detection limit of the microplate reader (saturated reading); the true value may be higher. This ceiling does not affect classification as strong biofilm former (A_{590} > 4×Ac), but biomass differences among saturated isolates cannot be resolved.

MH-CJ					
**Ac (Microaerophilic)**	**0.78**				
non-biofilm former	0.78	≥	A_{590}		
weak biofilm former	0.78	<	A_{590}	≤	1.56
moderate biofilm former	1.56	<	A_{590}	≤	3.12
strong biofilm former	3.12	<	A_{590}		
**Ac (Aerobic CO_2-enriched)**	**0.69**				
non-biofilm former	0.69	≥	A_{590}		
weak biofilm former	0.69	<	A_{590}	≤	1.38
moderate biofilm former	1.38	<	A_{590}	≤	2.76
strong biofilm former	2.76	<	A_{590}		
MH-HB					
**Ac (Microaerophilic)**	**0.83**				
non-biofilm former	0.83	≥	A_{590}		
weak biofilm former	0.83	<	A_{590}	≤	1.66

moderate biofilm former	1.66	<	A_{590}	≤	3.32
strong biofilm former	3.32	<	A_{590}		
**Ac (Aerobic CO_2-enriched)**	**0.99**				
non-biofilm former	0.99	≥	A_{590}		
weak biofilm former	0.99	<	A_{590}	≤	1.98
moderate biofilm former	1.98	<	A_{590}	≤	3.96
strong biofilm former	3.96	<	A_{590}		
