# Supplementary material for: Integrated Phenotypic and Genotypic Analysis Reveals Distinct Patterns of Biofilm Formation and Antimicrobial Resistance in Poultry-Derived Campylobacter jejuni and Campylobacter coli
Source: Foods. 2026 Oct 25;15(21):3815. doi: 10.3390/foods15213815 (PMC13763043; doi:10.3390/foods15213815)
Supplement: Supplementary file 1 [file foods-15-03815-s001.zip › Supplementary Table S4.pdf]

Table S4. Heatmap representation of species-specific biofilm-forming phenotypes among the major rep-PCR clusters of *C. jejuni* (up) and *C. coli* (down) isolates under either microaerophilic or aerobic CO_2-enriched conditions after 48 h incubation in MH-CJ medium at 42 °C. Percentages indicate the distribution of non- (N), weak (W), moderate (M), and strong (S) biofilm-forming phenotypes within each rep-PCR group. Minor profiles include rep-PCR clusters represented by fewer than ten isolates.

C. jejuni									
**rep-PCR cluster**		**Microaerophilic conditions**				**Aerobic CO_2-enriched conditions**			
		**N**	**W**	**M**	**S**	**N**	**W**	**M**	**S**
F	n = 11	45.5%	9.1%	9.1%	36.4%	36.4%	9.1%	18.2%	36.4%
H	n = 13	53.8%	0.0%	7.7%	38.5%	53.8%	0.0%	15.4%	30.8%
J	n = 27	63.0%	18.5%	0.0%	18.5%	70.4%	3.7%	7.4%	18.5%
Minor profiles	n = 19	78.9%	0.0%	0.0%	21.1%	84.2%	0.0%	0.0%	15.8%
ND	n = 8	100.0%	0.0%	0.0%	0.0%	100.0%	0.0%	0.0%	0.0%

C. coli									
**rep-PCR cluster**		**Microaerophilic conditions**				**Aerobic CO_2-enriched conditions**			
		**N**	**W**	**M**	**S**	**N**	**W**	**M**	**S**
F	n = 28	17.9%	25.0%	25.0%	32.1%	42.9%	17.9%	25.0%	14.3%
Minor profiles	n = 9	33.3%	11.1%	0.0%	55.6%	33.3%	33.3%	33.3%	0.0%
ND	n = 5	60.0%	20.0%	0.0%	20.0%	20.0%	60.0%	20.0%	0.0%

ND: rep-PCR profile not determined.
