# Supplementary material for: Integrated Phenotypic and Genotypic Analysis Reveals Distinct Patterns of Biofilm Formation and Antimicrobial Resistance in Poultry-Derived Campylobacter jejuni and Campylobacter coli
Source: Foods. 2026 Oct 25;15(21):3815. doi: 10.3390/foods15213815 (PMC13763043; doi:10.3390/foods15213815)
Supplement: Supplementary file 1 [file foods-15-03815-s001.zip › Supplementary Table S5.pdf]

Table S5. Species-adjusted association between rep-PCR cluster and biofilm formation (Firth logistic regression; n = 107 isolates with assigned cluster). Biofilm formation (yes/no; A_{590} > Ac) in MH-CJ medium was modelled separately for each atmospheric condition, with species (*C. coli vs C. jejuni*) and rep-PCR cluster (H, J and minor profiles *vs* F) as predictors. The 13 isolates without an assigned rep-PCR cluster (ND) were excluded.

A. Biofilm formers by rep-PCR cluster and species

	Microaerophilic			Aerobic CO_2-enriched		
**Group**	**Formers**	**Isolates**	**%**	**Formers**	**Isolates**	**%**
**All isolates with assigned cluster**	55	107	51.4	53	107	49.5
F cluster	29	39	74.4	30	39	76.9
H cluster	6	15	40.0	6	15	40.0
J cluster	11	29	37.9	9	29	31.0
Minor profiles	9	24	37.5	8	24	33.3
***C. jejuni* (all)**	26	70	37.1	24	70	34.3
F cluster	6	11	54.5	7	11	63.6
H cluster	6	13	46.2	6	13	46.2
J cluster	10	27	37.0	8	27	29.6
Minor profiles	4	19	21.1	3	19	15.8
***C. coli* (all)**	29	37	78.4	29	37	78.4
F cluster	23	28	82.1	23	28	82.1
H cluster	0	2	0.0	0	2	0.0
J cluster	1	2	50.0	1	2	50.0
Minor profiles	5	5	100.0	5	5	100.0

B. Species-adjusted logistic regression (Firth): odds ratios for biofilm formation

	Microaerophilic				Aerobic CO_2-enriched			
**Predictor (reference)**	**OR**	**95% CI lower**	**95% CI upper**	***p***	**OR**	**95% CI lower**	**95% CI upper**	***p***
C. coli (*vs C. jejuni*), adjusted for cluster	3.70	1.27	10.77	0.017	3.49	1.20	10.19	0.022
Cluster H (*vs* F), adjusted for species	0.48	0.12	1.94	0.307	0.41	0.10	1.65	0.209

Cluster J (*vs* F), adjusted for species	0.48	0.14	1.61	0.234	0.30	0.09	1.02	0.055
Minor profiles (*vs* F), adjusted for species	0.39	0.12	1.29	0.124	0.27	0.08	0.93	0.038
C. coli (*vs C. jejuni*), unadjusted (species-only model)	5.83	2.34	14.51	<0.001	6.59	2.63	16.48	<0.001

C. Likelihood ratio tests

	Microaerophilic			Aerobic CO_2-enriched		
**Test**	**χ^2**	**df**	***p***	**χ^2**	**df**	***p***
Cluster, without adjustment for species	17.07	3	<0.001	22.97	3	<0.001
Cluster, adjusted for species	5.98	3	0.112	8.78	3	0.032
Species, adjusted for cluster	7.53	1	0.006	6.81	1	0.009

D. F cluster compared with all other clusters

	Microaerophilic				Aerobic CO_2-enriched			
**Comparison**	**OR**	**95% CI lower**	**95% CI upper**	***p***	**OR**	**95% CI lower**	**95% CI upper**	***p***
Unadjusted (Fisher's exact test; sample OR)	4.68			<0.001	6.52			<0.001
Adjusted for species (Mantel-Haenszel OR; χ^2 test)	2.33	0.83	6.53	0.180	3.40	1.20	9.66	0.037
Within *C. jejuni*: formers in F *vs* other clusters (Fisher's exact test)	6/11 (54.5%)		20/59 (33.9%)	0.308	7/11 (63.6%)		17/59 (28.8%)	0.038
Within *C. coli*: formers in F *vs* other clusters (Fisher's exact test)	23/28 (82.1%)		6/9 (66.7%)	0.373	23/28 (82.1%)		6/9 (66.7%)	0.373

OR, odds ratio; CI, confidence interval. OR > 1 indicates higher odds of biofilm formation. Reference categories: C. jejuni (species) and the F cluster. Minor profiles are rep-PCR clusters represented by fewer than ten isolates.

Biofilm formation was defined as A_{590} > Ac (weak, moderate or strong) in MH-CJ medium, with Ac = 0.78 (microaerophilic) and 0.69 (aerobic CO_2-enriched) (Table S3).

Coefficients were estimated by Firth penalized-likelihood logistic regression (to handle small cells, e.g., two C. coli isolates each in clusters H and J). 95% CIs and p-values for individual coefficients are Wald-type, based on the penalized covariance matrix; cluster and species effects were also tested by likelihood ratio tests (penalized likelihood).

In Section D, the Mantel-Haenszel OR combines the species-specific 2×2 tables (F vs all other clusters); its 95% CI uses the Robins-Breslow-Greenland variance and its p-value the continuity-corrected Mantel-Haenszel χ^2 test.

Section D, within-species rows: formers/isolates (%) in the F cluster (first value) and in all other clusters (second value), compared by Fisher's exact test.

All analyses are exploratory and were not adjusted for multiple comparisons. The unadjusted OR for species differs slightly from that in Section 3.1 (5.64 and 6.34; n = 120, from 2×2 tables with Wald CIs) because it is estimated by Firth regression in the 107 isolates with an assigned cluster.
