# Supplementary material for: Integrated Phenotypic and Genotypic Analysis Reveals Distinct Patterns of Biofilm Formation and Antimicrobial Resistance in Poultry-Derived Campylobacter jejuni and Campylobacter coli
Source: Foods. 2026 Oct 25;15(21):3815. doi: 10.3390/foods15213815 (PMC13763043; doi:10.3390/foods15213815)
Supplement: Supplementary file 1 [file foods-15-03815-s001.zip › Supplementary Table S6.pdf]

Table S6. Inhibition zone diameters (mm) for the 10 antibiotics (E, TE, GEN, SXT, AMP, CIP, NOR, MEM, CTX, CAZ) against the 120 *Campylobacter* isolates, as determined by the disk diffusion method. The inhibition zone diameters against the reference strain *C. jejuni* ATCC 33291, along with the summary of inhibition zone diameters per antibiotic and species, are also presented.

			Inhibition zone diameters (mm)									
**s/n**	**Isolation ID**	**Species**	**E**	**TE**	**GEN**	**SXT**	**AMP**	**CIP**	**NOR**	**MEM**	**CTX**	**CAZ**
C	ATCC 33291	*C. jejuni*	30	40	26	17	18	33	30	40	18	12
1	POUL007/CAMP001	*C. jejuni*	42	13	33	18	1	10	1	50	11	21
2	POUL010/CAMP003	*C. jejuni*	30	31	28	1	22	1	1	52	1	11
3	POUL007/CAMP004	*C. coli*	30	1	30	27	26	1	1	49	1	20
4	POUL007/CAMP005	*C. coli*	25	1	25	23	22.5	1	1	40	1	10
5	POUL008/CAMP006	*C. jejuni*	37	11	29.5	17	1	11	1	46	15.5	19
6	POUL009/CAMP007	*C. jejuni*	37	1	27.5	1	1	1	1	33	1	15
7	POUL009/CAMP008	*C. jejuni*	35	9	29	1	1	1	1	36.5	10	1
8	POUL010/CAMP009	*C. jejuni*	35	35	32	1	23	1	1	50	10	10
9	POUL010/CAMP010	*C. coli*	27	1	24	25	20	35.5	34	38	10	15
10	POUL012/CAMP011	*C. jejuni*	32.5	1	30	20	1	12	1	45	9	11
11	POUL012/CAMP012	*C. jejuni*	33.5	1	29	22	1	10	1	45	10	15.5
12	POUL012/CAMP013	*C. jejuni*	30	1	27	15	1	9	1	37.5	12	20
13	POUL015/CAMP014	*C. coli*	29	13.5	25	13	20.5	38.5	39	34.4	12.5	29
14	POUL015/CAMP015	*C. coli*	29	12	25.5	22	21	40	39	45	15	17
15	POUL015/CAMP016	*C. coli*	26	9	24.5	18	23	35	36	41	23.5	30
16	POUL015/CAMP017	*C. coli*	28	11	29	24	26	35	38	45	23	30
17	POUL011/CAMP018	*C. jejuni*	29	1	28	1	18	1	1	48	1	1
18	POUL012/CAMP019	*C. jejuni*	27	1	22	19	1	1	1	40.5	11	20
19	POUL014/CAMP020	*C. jejuni*	40	12	30	20	10	11.5	1	40.5	16	17
20	POUL015/CAMP021	*C. coli*	35	13	35	18	15	45	40	47	11.5	16.5
21	POUL011/CAMP022	*C. jejuni*	29.5	1	24	1	19	1	1	45	1	1
22	POUL012/CAMP023	*C. jejuni*	40	1	35	1	1	12	1	42.5	1	14
23	POUL015/CAMP024	*C. jejuni*	39	16.5	32	1	1	10	1	43	19	22
24	POUL016/CAMP025	*C. coli*	19	32	10	21.5	1	1	1	31	1	1
25	POUL016/CAMP026	*C. coli*	28.5	41	28	27	1	1	1	34	9	10
26	POUL016/CAMP027	*C. jejuni*	29	18.5	31	1	24	1	1	54	11	1
27	POUL016/CAMP028	*C. jejuni*	30	16.5	28	13	25.5	1	1	51	14	14
28	POUL016/CAMP029	*C. jejuni*	32	19	31	1	1	1	1	43	14	14
29	POUL019/CAMP030	*C. coli*	32	12	29	1	21	1	1	50	9	12
30	POUL016/CAMP031	*C. coli*	29	44	29	29	1	1	1	36	1	1
31	POUL017/CAMP032	*C. coli*	37.5	50	30	29	29	17	1	46	13	18
32	POUL017/CAMP033	*C. coli*	31	43	27.5	25	24	1	1	46	1	12
33	POUL017/CAMP034	*C. coli*	41	11	33	30	28	17.5	1	44.5	12	15
34	POUL019/CAMP035	*C. jejuni*	36	12.5	30.5	16.5	26.5	12.5	1	52	10	13
35	POUL019/CAMP036	*C. jejuni*	35.5	13	31	1	21	1	1	55	11	13
36	POUL025/CAMP037	*C. jejuni*	33	13	36	18	25	13	1	59	1	12
37	POUL021/CAMP038	*C. coli*	35	13	28	27	18	13.5	1	48	11	17
38	POUL022/CAMP039	*C. coli*	38.5	43.5	29	25	25	16	1	48	11	16.5
39	POUL023/CAMP040	*C. jejuni*	26	34	27	1	1	1	1	39	1	1
40	POUL024/CAMP041	*C. jejuni*	26	34	27	1	1	1	1	39	1	1
41	POUL025/CAMP042	*C. coli*	29	1	29	23	23	1	1	48	1	11

42	POUL026/CAMP043	*C. jejuni*	29	10	25	22	26	40.5	45	57	22	22
43	POUL025/CAMP044	*C. coli*	38.5	14	30	30	1	15	1	44	19	19
44	POUL026/CAMP045	*C. jejuni*	32.5	1	28	16	21.5	41.5	44	63	18	18
45	POUL028/CAMP046	*C. jejuni*	34.5	43	26	18	1	1	1	60	12	12
46	POUL027/CAMP048	*C. jejuni*	35	44	27	1	31	40	44	51	16	18
47	POUL028/CAMP049	*C. jejuni*	34	43.5	28	1	1	1	1	53	1	13
48	POUL029/CAMP050	*C. jejuni*	32	40	27	1	31	41	48	60	12	12
49	POUL030/CAMP051	*C. jejuni*	28	38	28	1	26	1	1	57	11	11
50	POUL028/CAMP052	*C. jejuni*	32	42	29	1	1	1	1	57	10	11
51	POUL029/CAMP053	*C. jejuni*	31	44	31	1	31	47	50	59	11	12
52	POUL031/CAMP054	*C. jejuni*	34	40	31	17	1	1	1	59	1	1
53	POUL032/CAMP056	*C. coli*	40	44	27	1	17.5	15	1	49	17.5	17
54	POUL032/CAMP057	*C. coli*	37.5	44	26	1	18	15.5	1	56	16	16
55	POUL032/CAMP058	*C. jejuni*	40	14	34	1	22.5	13	1	64	10	10
56	POUL033/CAMP059	*C. jejuni*	34	22	27	1	1	1	1	41	12	1
57	POUL033/CAMP062	*C. jejuni*	36	22	26	1	1	1	1	41	12	1
58	POUL036/CAMP063	*C. jejuni*	38	1	29	1	1	1	1	44	12	1
59	POUL037/CAMP064	*C. jejuni*	38	1	30	1	31	1	1	50	1	18
60	POUL037/CAMP065	*C. jejuni*	37	1	31	16	30	1	1	51	14	19
61	POUL038/CAMP066	*C. jejuni*	36	14	27.5	1	31	1	1	51	15	20
62	POUL038/CAMP067	*C. jejuni*	37	18.5	29	1	32	1	1	51	18	19
63	POUL041/CAMP068	*C. jejuni*	40	49	29	1	25	1	1	51	15	19
64	POUL037/CAMP069	*C. jejuni*	29	1	32	1	30	1	1	59	1	13.5
65	POUL040/CAMP070	*C. jejuni*	25	37	25	1	18	26	26	44	15	1
66	POUL041/CAMP071	*C. coli*	27	32	23	23.5	26.5	29	31	36	13	15
67	POUL037/CAMP072	*C. coli*	34	1	30	1	30	1	1	50	1	10
68	POUL037/CAMP073	*C. jejuni*	32.5	1	26	1	44	11	17	66	23	30
69	POUL038/CAMP074	*C. jejuni*	31	17	26	1	43	1	1	50.5	29	24
70	POUL038/CAMP075	*C. jejuni*	31	16	28	1	46	1	1	70	23	24
71	POUL040/CAMP076	*C. jejuni*	24	33	28	1	17	28	36	71	15.5	22
72	POUL041/CAMP077	*C. jejuni*	28	37	27	1	24.5	1	1	73	20	26
73	POUL041/CAMP078	*C. jejuni*	30	37	24	12	17	35	56	67	13	13
74	POUL045/CAMP080	*C. coli*	27	13	25.5	26.5	1	13	1	31	10	27
75	POUL045/CAMP081	*C. coli*	30	40	28	16.5	18	1	1	55	15	21
76	POUL044/CAMP082	*C. coli*	36	41	27	1	16	15	1	58	21.5	24.5
77	POUL044/CAMP083	*C. coli*	33	37	24	1	18	27	40	48	15	15
78	POUL042/CAMP084	*C. jejuni*	27	35	24	1	25	1	1	60	15	18
79	POUL042/CAMP085	*C. jejuni*	23	35	23	1	24	1	1	47	15	1
80	POUL043/CAMP086	*C. jejuni*	25	26	23	1	30	26	28	38	15	13.5
81	POUL043/CAMP087	*C. jejuni*	23	14	24	1	1	1	1	42	1	1
82	POUL044/CAMP088	*C. jejuni*	30	17	23	1	22	12	1	51	16	18
83	POUL044/CAMP089	*C. coli*	29	36	23	15	16	1	1	43	10	20
84	POUL044/CAMP090	*C. coli*	25	34	24	1	16	32.5	32	42	15	1
85	POUL045/CAMP091	*C. jejuni*	18.5	24	33	23	29	33	32	42	32	24
86	POUL045/CAMP092	*C. coli*	27	34	23	16	13.5	1	1	40	1	15
87	POUL046/CAMP093	*C. jejuni*	23	1	24	1	1	1	1	35	11	1
88	POUL046/CAMP094	*C. jejuni*	45	1	40	1	1	1	1	40	1	12
89	POUL047/CAMP095	*C. coli*	34	26	1	20.5	19	1	1	40	10.5	1

90	POUL048/CAMP096	*C. coli*	46	13.5	36	24	20	13.5	1	39	13	9
91	POUL047/CAMP097	*C. coli*	1	12	46	25	1	1	1	41	1	18
92	POUL048/CAMP098	*C. coli*	33	34.5	24	14	22	36	33	40	24	16
93	POUL048/CAMP099	*C. coli*	33	11	28.5	22	23.5	1	1	38	12.5	25
94	POUL049/CAMP100	*C. jejuni*	24	37	24	1	17	30	23.5	41.5	9	17
95	POUL048/CAMP102	*C. coli*	29	12	26	19	23	1	1	42	14	9
96	POUL048/CAMP103	*C. jejuni*	27	37	25	1	18	27	29	56	11	17
97	POUL048/CAMP104	*C. jejuni*	29	32	24	1	1	1	1	34	10	11
98	POUL048/CAMP105	*C. jejuni*	29.5	11.5	28	1	1	1	1	50	1	11
99	POUL048/CAMP106	*C. jejuni*	33.5	39	25	1	1	1	1	49	11	1
100	POUL053/CAMP107	*C. coli*	36	12.5	28.5	17	22	13	1	52	12	11
101	POUL055/CAMP108	*C. jejuni*	28	1	27	1	1	1	1	66	11	15
102	POUL056/CAMP109	*C. jejuni*	29	36	25	1	21	1	1	55	1	13
103	POUL053/CAMP110	*C. coli*	31	39.5	24	20	20	1	1	45	14	15
104	POUL055/CAMP111	*C. jejuni*	26	1	28	1	1	1	1	39	12	14
105	POUL056/CAMP112	*C. jejuni*	32.5	38	25	1	19	10	1	45	13	19
106	POUL056/CAMP113	*C. jejuni*	31.5	41	24	1	19	1	1	57	16	16.5
107	POUL055/CAMP114	*C. jejuni*	35	1	28	16	1	1	1	39	11	19.5
108	POUL053/CAMP117	*C. jejuni*	32	34	20	21	16	1	1	44	1	11
109	POUL057/CAMP119	*C. coli*	31	41	23	24	18	1	1	41	14	1
110	POUL057/CAMP121	*C. coli*	24	11	24	22	19	1	1	40	10	13
111	POUL058/CAMP122	*C. coli*	29	9	26.5	21	1	1	1	35	11	10
112	POUL060/CAMP123	*C. jejuni*	27	35	24	1	20	31.5	27	45	11	11
113	POUL060/CAMP124	*C. jejuni*	29	38	25	1	17	29	27	46	1	1
114	POUL061/CAMP125	*C. jejuni*	26	37	24	1	20	27	26	46	1	1
115	POUL062/CAMP126	*C. jejuni*	31	21	26	1	23	1	1	42	12	12
116	POUL063/CAMP127	*C. coli*	31	37	26	10.5	15	10	1	41	1	21
117	POUL062/CAMP129	*C. jejuni*	29	17	26.5	1	23	1	1	40	15	14
118	POUL064/CAMP130	*C. jejuni*	25	19	20	26.5	26	25	23	29	18	17
119	POUL061/CAMP131	*C. jejuni*	27	38	25	13	18.5	25	26	46	1	1
120	POUL062/CAMP132	*C. jejuni*	9	31	33	1	23	29.5	29	41	13	13

A value of 1 mm indicates no visible inhibition zone around the disc (disc diameter 6 mm); it is not a measured zone diameter.

Summary of inhibition zone diameters (mm) per antibiotic and species

Statistic	Group	E	TE	GEN	SXT	AMP	CIP	NOR	MEM	CTX	CAZ
Isolates tested (n)	**All isolates**	120	120	120	120	120	120	120	120	120	120
No visible inhibition (n)	All isolates	1	24	1	64	36	66	91	0	30	23
No visible inhibition (%)	All isolates	0.8%	20.0%	0.8%	53.3%	30.0%	55.0%	75.8%	0.0%	25.0%	19.2%
Inhibited isolates (n)	All isolates	119	96	119	56	84	54	29	120	90	97
Median zone, inhibited isolates	All isolates	31.0	32.0	27.0	20.8	22.3	25.0	33.0	45.5	13.0	15.5
Minimum zone, inhibited isolates	All isolates	9.0	9.0	10.0	10.5	10.0	9.0	17.0	29.0	9.0	9.0
Maximum zone, inhibited isolates	All isolates	46.0	50.0	46.0	30.0	46.0	47.0	56.0	73.0	32.0	30.0
Isolates tested (n)	***C. jejuni***	78	78	78	78	78	78	78	78	78	78
No visible inhibition (n)	*C. jejuni*	0	19	0	57	29	46	59	0	20	18
No visible inhibition (%)	*C. jejuni*	0.0%	24.4%	0.0%	73.1%	37.2%	59.0%	75.6%	0.0%	25.6%	23.1%
Inhibited isolates (n)	*C. jejuni*	78	59	78	21	49	32	19	78	58	60
Median zone, inhibited isolates	*C. jejuni*	31.0	32.0	27.5	18.0	23.0	25.5	29.0	48.5	12.5	15.0
Minimum zone, inhibited isolates	*C. jejuni*	9.0	9.0	20.0	12.0	10.0	9.0	17.0	29.0	9.0	10.0

Maximum zone, inhibited isolates	*C. jejuni*	45.0	49.0	40.0	26.5	46.0	47.0	56.0	73.0	32.0	30.0
Isolates tested (n)	***C. coli***	42	42	42	42	42	42	42	42	42	42
No visible inhibition (n)	*C. coli*	1	5	1	7	7	20	32	0	10	5
No visible inhibition (%)	*C. coli*	2.4%	11.9%	2.4%	16.7%	16.7%	47.6%	76.2%	0.0%	23.8%	11.9%
Inhibited isolates (n)	*C. coli*	41	37	41	35	35	22	10	42	32	37
Median zone, inhibited isolates	*C. coli*	31.0	32.0	27.0	23.0	20.5	17.3	37.0	42.5	13.0	16.0
Minimum zone, inhibited isolates	*C. coli*	19.0	9.0	10.0	10.5	13.5	10.0	31.0	31.0	9.0	9.0
Maximum zone, inhibited isolates	*C. coli*	46.0	50.0	46.0	30.0	30.0	45.0	40.0	58.0	24.0	30.0

No visible inhibition: recorded diameter 1 mm. Inhibited isolates: any measurable inhibition zone.

Median, minimum, and maximum are calculated for inhibited isolates only.

The reference strain *C. jejuni* ATCC 33291 is excluded from all summary statistics
