# Supplementary material for: Integrated Phenotypic and Genotypic Analysis Reveals Distinct Patterns of Biofilm Formation and Antimicrobial Resistance in Poultry-Derived Campylobacter jejuni and Campylobacter coli
Source: Foods. 2026 Oct 25;15(21):3815. doi: 10.3390/foods15213815 (PMC13763043; doi:10.3390/foods15213815)
Supplement: Supplementary file 1 [file foods-15-03815-s001.zip › Supplementary Table S7.pdf]

Table S7. Heatmap-based visualization of combined no-inhibition phenotypes among *C. jejuni* and *C. coli* isolates for antibiotics lacking established EUCAST clinical breakpoints (GEN, AMP, SXT, NOR, MEM, CAZ, and CTX). No-inhibition phenotypes describe the absence of a visible inhibition zone and are not resistance categories. Phenotypes were grouped by shared antibiotic combinations and overall distribution trends. The left heatmap (Prevalences within species) shows the frequency (%) of each no-inhibition phenotype relative to the total number of isolates within each species (*C. jejuni*, n = 78; *C. coli*, n = 42) and within the total isolate collection (n = 120). The right heatmap (Species contribution within phenotype) shows the proportional contribution (%) of each species within a given no-inhibition phenotype. The column "n" indicates the total number of isolates assigned to each phenotype. Absence of visible inhibition was recorded as an inhibition zone diameter of 1 mm.

Phenotype category	No-inhibition phenotype	n	Prevalence within species			Species contribution within phenotype	
			C. jejuni	*C. coli*	All isolates	*C. jejuni*	*C. coli*
Simple phenotype	none	13	6.4%	19.0%	10.8%	38.5%	61.5%
	NOR	16	5.1%	28.6%	13.3%	25.0%	75.0%
	SXT	11	12.8%	2.4%	9.2%	90.9%	9.1%
	AMP+ NOR	12	10.3%	9.5%	10.0%	66.7%	33.3%
	NOR+ CTX	8	2.6%	14.3%	6.7%	25.0%	75.0%
SXT+ NOR-associated phenotype	SXT+ NOR	20	20.5%	9.5%	16.7%	80.0%	20.0%
	SXT+ NOR+ CTX	5	5.1%	2.4%	4.2%	80.0%	20.0%
	SXT+ NOR+ CAZ	2	2.6%	0.0%	1.7%	100.0%	0.0%
	SXT+ NOR+ CTX+ CAZ	2	2.6%	0.0%	1.7%	100.0%	0.0%
Expanded SXT+ AMP+ NOR phenotype	SXT+ AMP+ NOR	6	7.7%	0.0%	5.0%	100.0%	0.0%
	SXT+ AMP+ NOR+ CAZ	6	7.7%	0.0%	5.0%	100.0%	0.0%
	SXT+ AMP+ NOR+ CTX	5	6.4%	0.0%	4.2%	100.0%	0.0%
	SXT+ AMP+ NOR+ CTX+ CAZ	3	3.8%	0.0%	2.5%	100.0%	0.0%
Low-frequency phenotype	NOR+ CAZ	1	0.0%	2.4%	0.8%	0.0%	100.0%
	SXT+ CAZ	2	1.3%	2.4%	1.7%	50.0%	50.0%
	AMP+ NOR+ CTX	1	0.0%	2.4%	0.8%	0.0%	100.0%
	AMP+ NOR+ CTX+ CAZ	3	1.3%	4.8%	2.5%	33.3%	66.7%
	SXT+ CTX+ CAZ	2	2.6%	0.0%	1.7%	100.0%	0.0%
	CTX+ CAZ	1	1.3%	0.0%	0.8%	100.0%	0.0%
	GEN+ NOR+ CAZ	1	0.0%	2.4%	0.8%	0.0%	100.0%
