# Supplementary material for: Integrated Phenotypic and Genotypic Analysis Reveals Distinct Patterns of Biofilm Formation and Antimicrobial Resistance in Poultry-Derived Campylobacter jejuni and Campylobacter coli
Source: Foods. 2026 Oct 25;15(21):3815. doi: 10.3390/foods15213815 (PMC13763043; doi:10.3390/foods15213815)
Supplement: Supplementary file 1 [file foods-15-03815-s001.zip › Supplementary Table S8.pdf]

Table S8. Heatmap representation of AMR phenotypes across the major rep-PCR clusters of the total *Campylobacter* collection (top), *C. jejuni* (middle), and *C. coli* (bottom). Heatmap values represent the distribution (%) of rep-PCR clusters within each AMR phenotype. The rightmost column indicates the overall prevalence (%) of each rep-PCR cluster in the corresponding isolate collection. Minor profiles include rep-PCR clusters represented by fewer than ten isolates.

**rep-PCR cluster**	***Campylobacter* spp. - Resistance to antibiotics**							**Prevalence among *Campylobacter* spp. isolates (n = 120)**
	**susc (n = 15)**	**E (n = 1)**	**TE (n = 9)**	**CIP (n = 35)**	**E+TE (n = 1)**	**TE+CIP (n = 58)**	**E+TE+CIP (n = 1)**	
F (n = 39)	26.7%	0.0%	77.8%	37.1%	0.0%	24.1%	100.0%	32.5%
H (n = 15)	13.3%	0.0%	22.2%	5.7%	0.0%	15.5%	0.0%	12.5%
J (n = 29)	46.7%	100.0%	0.0%	17.1%	100.0%	24.1%	0.0%	24.2%
ND (n = 13)	6.7%	0.0%	0.0%	17.1%	0.0%	10.3%	0.0%	10.8%
Minor profiles (n = 24)	6.7%	0.0%	0.0%	22.9%	0.0%	25.9%	0.0%	20.0%

	***C. jejuni* - Resistance to antibiotics**						**Prevalence among *C. jejuni* isolates (n = 78)**
	**susc (n = 11)**	**E (n = 1)**	**TE (n = 3)**	**CIP (n = 20)**	**E+TE (n = 1)**	**TE+CIP (n = 42)**	
F (n = 11)	27.3%	0.0%	33.3%	10.0%	0.0%	11.9%	14.1%
H (n = 13)	18.2%	0.0%	66.7%	10.0%	0.0%	16.7%	16.7%
J (n = 27)	54.5%	100.0%	0.0%	30.0%	100.0%	31.0%	34.6%
Minor & ND profiles (n = 27)	0.0%	0.0%	0.0%	50.0%	0.0%	40.5%	34.6%

	***C. coli* - Resistance to antibiotics**					**Prevalence among *C. coli* isolates (n = 42)**
	**susc (n = 4)**	**TE (n = 6)**	**CIP (n = 15)**	**TE+CIP (n = 16)**	**E+TE+CIP (n = 1)**	
F (n = 28)	25.0%	100.0%	73.3%	56.3%	100.0%	66.7%
Minor & ND profiles (n = 14)	75.0%	0.0%	26.7%	43.8%	0.0%	33.3%

ND: rep-PCR profile not detected.

susc: susceptible
