# Supplementary material for: Integrated Phenotypic and Genotypic Analysis Reveals Distinct Patterns of Biofilm Formation and Antimicrobial Resistance in Poultry-Derived Campylobacter jejuni and Campylobacter coli
Source: Foods. 2026 Oct 25;15(21):3815. doi: 10.3390/foods15213815 (PMC13763043; doi:10.3390/foods15213815)
Supplement: Supplementary file 1 [file foods-15-03815-s001.zip › Supplementary Table S9.pdf]

Table S9. Heatmap representation of combined no-inhibition phenotypes across the major rep-PCR clusters of the total *Campylobacter* collection (top), *C. jejuni* (middle), and *C. coli* (bottom). No-inhibition phenotypes describe the absence of a visible inhibition zone and are not resistance categories. Heatmap values represent the distribution (%) of each no-inhibition phenotype within the corresponding rep-PCR cluster. The rightmost column indicates the overall prevalence (%) of each phenotype in the respective isolate collection. Minor profiles include rep-PCR clusters represented by fewer than ten isolates. Absence of visible inhibition was recorded as an inhibition zone diameter of 1 mm.

**No-inhibition phenotype for *Campylobacter* spp. isolates**	**rep-PCR cluster**					**Prevalence among *Campylobacter* spp. isolates (n = 120)**
	**F (n = 39)**	**H (n = 15)**	**J (n = 29)**	**ND (n = 13)**	**Minor profiles (n = 24)**	
SXT+ NOR	20.0%	10.0%	45.0%	10.0%	15.0%	16.7%
NOR	50.0%	0.0%	6.3%	6.3%	37.5%	13.3%
none	69.2%	15.4%	7.7%	0.0%	7.7%	10.8%
AMP+ NOR	33.3%	0.0%	25.0%	16.7%	25.0%	10.0%
SXT	18.2%	18.2%	63.6%	0.0%	0.0%	9.2%
NOR+ CTX	75.0%	0.0%	0.0%	12.5%	12.5%	6.7%
SXT+ AMP+ NOR+ CAZ	16.7%	0.0%	33.3%	33.3%	16.7%	5.0%
SXT+ AMP+ NOR	16.7%	33.3%	16.7%	16.7%	16.7%	5.0%
SXT+ NOR+ CTX	0.0%	40.0%	60.0%	0.0%	0.0%	4.2%
SXT+ AMP+ NOR+ CTX	0.0%	20.0%	0.0%	0.0%	80.0%	4.2%
AMP+ NOR+ CTX+ CAZ	66.7%	0.0%	0.0%	0.0%	33.3%	2.5%
SXT+ AMP+ NOR+ CTX+ CAZ	0.0%	0.0%	33.3%	0.0%	66.7%	2.5%
SXT+ NOR+ CTX+ CAZ	0.0%	100.0%	0.0%	0.0%	0.0%	1.7%
SXT+ NOR+ CAZ	0.0%	0.0%	0.0%	50.0%	50.0%	1.7%
SXT+ CTX+ CAZ	0.0%	50.0%	50.0%	0.0%	0.0%	1.7%
SXT+ CAZ	50.0%	0.0%	0.0%	50.0%	0.0%	1.7%
AMP+ NOR+ CTX	100.0%	0.0%	0.0%	0.0%	0.0%	0.8%
CTX+ CAZ	0.0%	100.0%	0.0%	0.0%	0.0%	0.8%
GEN+ NOR+ CAZ	0.0%	0.0%	0.0%	100.0%	0.0%	0.8%
NOR+ CAZ	0.0%	0.0%	0.0%	100.0%	0.0%	0.8%

No-inhibition phenotype for *C. jejuni* isolates	rep-PCR cluster F (n = 11)	H (n = 13)	J (n = 27)	Minor & ND profiles (n = 27)	Prevalence among *C. jejuni* isolates (n = 78)
SXT+ NOR	6.3%	6.3%	56.3%	31.3%	20.5%
SXT	20.0%	20.0%	60.0%	0.0%	12.8%
AMP+ NOR	12.5%	0.0%	37.5%	50.0%	10.3%
SXT+ AMP+ NOR	16.7%	33.3%	16.7%	33.3%	7.7%
SXT+ AMP+ NOR+ CAZ	16.7%	0.0%	33.3%	50.0%	7.7%
none	40.0%	40.0%	20.0%	0.0%	6.4%
SXT+ AMP+ NOR+ CTX	0.0%	20.0%	0.0%	80.0%	6.4%
NOR	50.0%	0.0%	0.0%	50.0%	5.1%
SXT+ NOR+ CTX	0.0%	25.0%	75.0%	0.0%	5.1%
SXT+ AMP+ NOR+ CTX+ CAZ	0.0%	0.0%	33.3%	66.7%	3.8%
NOR+ CTX	0.0%	0.0%	0.0%	100.0%	2.6%
SXT+ CTX+ CAZ	0.0%	50.0%	50.0%	0.0%	2.6%
SXT+ NOR+ CAZ	0.0%	0.0%	0.0%	100.0%	2.6%
SXT+ NOR+ CTX+ CAZ	0.0%	100.0%	0.0%	0.0%	2.6%
AMP+ NOR+ CTX+ CAZ	0.0%	0.0%	0.0%	100.0%	1.3%
CTX+ CAZ	0.0%	100.0%	0.0%	0.0%	1.3%
SXT+ CAZ	100.0%	0.0%	0.0%	0.0%	1.3%

No-inhibition phenotype for *C. coli* isolates	rep-PCR cluster F (n = 28)	Minor & ND profiles (n = 14)	Prevalence among *C. coli* isolates (n = 42)
NOR	50.0%	50.0%	28.6%
none	87.5%	12.5%	19.0%
NOR+ CTX	100.0%	0.0%	14.3%
AMP+ NOR	75.0%	25.0%	9.5%
SXT+ NOR	75.0%	25.0%	9.5%

AMP+ NOR+ CTX+ CAZ	100.0%		0.0%	4.8%
AMP+ NOR+ CTX	100.0%		0.0%	2.4%
GEN+ NOR+ CAZ	0.0%		100.0%	2.4%
NOR+ CAZ	0.0%		100.0%	2.4%
SXT	0.0%		100.0%	2.4%
SXT+ CAZ	0.0%		100.0%	2.4%
SXT+ NOR+ CTX	0.0%		100.0%	2.4%

ND: rep-PCR profile not detected.
